# Distinct molecular subgroups in pediatric and young-onset meningiomas require age-adapted risk stratification

**DOI:** 10.1038/s41467-026-75357-2

**Published:** 2026-07-14

**Authors:** Natalie Berghaus, Arnault Tauziède-Espariat, Thomas Hielscher, Dilan Savran, Daniel Schrimpf, Kirsten Göbel, Eric Stutheit-Zhao, Lukas Friedrich, Felix Keller, Fuat Kaan Aras, Filippo Nozzoli, Dominik Sturm, Christine L. White, Simone Schmid, Christian Mawrin, Julia E. Neumann, Till Acker, Rudi Beschorner, Christian Hartmann, Irem Saribiyik, Arda Inan, Ayça Erşen-Danyeli, Mariëtte E. G. Kranendonk, Sybren L. N. Maas, Eelke M. Bos, Eleonora Aronica, Saskia M. Peerdeman, Nikki B. Thuijs, Angelika Mühlebner, Benno Kusters, Wilfred F. A. den Dunnen, Cinzia E. Lavarino, Stéphanie Puget, Jason Chiang, Sonika Dahiya, Melike Pekmezci, Arie Perry, Oluwadamilola Akanji, Miriam Ratliff, Christel Herold-Mende, Sandro M. Krieg, Wolfgang Wick, Stefan M. Pfister, Pieter Wesseling, Andreas von Deimling, Pascale Varlet, Felix Sahm, Philipp Sievers

**Affiliations:** 1 Department of Neuropathology, Institute of Pathology, University Hospital Heidelberg, Heidelberg, Germany; 2 Clinical Cooperation Unit Neuropathology, German Consortium for Translational Cancer Research (DKTK), German Cancer Research Center (DKFZ), Heidelberg, Germany; 3 Department of Neuropathology, GHU Paris, Psychiatry and Neurosciences, Sainte-Anne Hospital, Paris, France; 4 Université Paris Cité, Institute of Psychiatry and Neuroscience of Paris (IPNP), Paris, France; 5 Department of Biostatistics, German Cancer Research Center (DKFZ), Heidelberg, Germany; 6 Princess Margaret Cancer Centre, University Health Network, Toronto, Ontario, Canada; 7 Section of Anatomic Pathology, Department of Health Sciences, University of Florence, Florence, Italy; 8 Histopathology and Molecular Diagnostics, Careggi University Hospital, Florence, Italy; 9 Hopp Children’s Cancer Center Heidelberg (KiTZ), Heidelberg, Germany; 10 Division of Pediatric Glioma Research, German Cancer Research Center (DKFZ), Heidelberg, Germany; 11 Department of Pediatric Oncology, Hematology, Immunology and Pulmonology, University Hospital Heidelberg, Heidelberg, Germany; 12 National Center for Tumor Diseases (NCT), NCT Heidelberg, a partnership between DKFZ and Heidelberg University Hospital, Heidelberg, Germany; 13 Genetics and Molecular Pathology Laboratory, Hudson Institute of Medical Research, Clayton, VIC Australia; 14 Department of Molecular and Translational Science, Monash University, Melbourne, VIC Australia; 15 Victorian Clinical Genetics Services, Parkville, VIC Australia; 16 Department of Neuropathology, Charité-Universitätsmedizin Berlin, Corporate Member of Freie Universität Berlin and Humboldt-Universität zu Berlin, Berlin, Germany; 17 German Cancer Consortium (DKTK), Partner Site Berlin, German Cancer Research Center (DKFZ), Heidelberg, Germany; 18 Department of Neuropathology, Otto-von-Guericke-University, Magdeburg, Germany; 19 Institute of Neuropathology, University Medical Center Hamburg-Eppendorf, Hamburg, Germany; 20 Center for Molecular Neurobiology Hamburg, University Medical Center Hamburg-Eppendorf, Hamburg, Germany; 21 Institute of Neuropathology, Justus-Liebig University Giessen, Giessen, Germany; 22 Center for Neuro-Oncology, Comprehensive Cancer Center Tübingen-Stuttgart, University Hospital Tübingen, Eberhard-Karls-University Tübingen, Tübingen, Germany; 23 Department of Neuropathology, University of Tübingen, Eberhard-Karls-University Tübingen, Tübingen, Germany; 24 Department of Neuropathology, Institute of Pathology, Hannover Medical School (MHH), Hannover, Germany; 25 Department of Pathology, Gazi University, Ankara, Turkey; 26 Department of Pathology, Acibadem Universitesi Tip Fakultesi, Istanbul, Turkey; 27 Princess Máxima Center for Pediatric Oncology, Utrecht, The Netherlands; 28 Department of Pathology, Brain Tumor Center, Erasmus MC Cancer Institute, University Medical Center Rotterdam, Rotterdam, The Netherlands; 29 Department of Pathology, Leiden University Medical Center, Leiden, The Netherlands; 30 Department of Neurosurgery, Brain Tumor Center, Erasmus MC Cancer Institute, Erasmus University Medical Center, Rotterdam, The Netherlands; 31 Department of (Neuro) Pathology, Amsterdam Neuroscience, Amsterdam UMC Location University of Amsterdam, Amsterdam, The Netherlands; 32 Department of Neurosurgery, Amsterdam University Medical Center, Amsterdam UMC Location VU University, Amsterdam, The Netherlands; 33 Department of Pathology, Amsterdam UMC Location Vrije Universiteit Amsterdam, Amsterdam, The Netherlands; 34 Cancer Center Amsterdam, Imaging and Biomarkers, Amsterdam, The Netherlands; 35 Department of Pathology, University Medical Center Utrecht, Utrecht, The Netherlands; 36 Department of Pathology, Radboudumc, Nijmegen, The Netherlands; 37 Department of Pathology and Medical Biology, University Medical Centre Groningen, University of Groningen, Groningen, The Netherlands; 38 Laboratory of Molecular Oncology, Pediatric Cancer Center Barcelona, Hospital Sant Joan de Déu, Barcelona, Spain; 39 Department of Neurosurgery, CHU Martinique, Fort-de-France, France; 40 Department of Pathology, St. Jude Children’s Research Hospital, Memphis, TN USA; 41 Department of Pathology and Immunology, Washington University School of Medicine, St Louis, MO USA; 42 Department of Pathology, University of California San Francisco (UCSF), San Francisco, CA USA; 43 Department of Neurological Surgery, University of California San Francisco (UCSF), San Francisco, CA USA; 44 Department of Neurosurgery, University Hospital Mannheim, Medical Faculty Mannheim, University of Heidelberg, Mannheim, Germany; 45 Clinical Cooperation Unit Neurooncology, German Consortium for Translational Cancer Research (DKTK), German Cancer Research Center (DKFZ), Heidelberg, Germany; 46 Department of Neurosurgery, Heidelberg University Hospital, Heidelberg, Germany; 47 Department of Neurology and Neurooncology Program, European Center for Neurooncology (EZN), Heidelberg University Hospital, Heidelberg University, Heidelberg, Germany; 48 Division of Pediatric Neurooncology, German Consortium for Translational Cancer Research (DKTK), German Cancer Research Center (DKFZ), Heidelberg, Germany; 49 Department of Pathology, Amsterdam University Medical Centers, Location VUmc and Brain Tumor Center Amsterdam, Amsterdam, The Netherlands

**Keywords:** Paediatric research, Prognostic markers, Molecular medicine, CNS cancer

## Abstract

Meningiomas in pediatric and adolescent/young adult patients are poorly characterized biologically and clinically, and risk stratification is largely extrapolated from adult tumors. We analyze 293 tumors from patients aged 0–39 years using integrated histopathological and molecular profiling. Youth-onset meningiomas are enriched for *NF2* and *SMARCE1* alterations and exhibit a gain-dominated copy-number landscape, including recurrent chr17q gain, whereas canonical adult high-risk features, such as chr1p loss, lack prognostic significance. Adult-derived prognostic frameworks, including WHO grade, methylation-based stratification and integrated risk scores, fail to predict progression in patients ≤21 years of age. Tumors segregate into age-enriched epigenetic clusters defined by *SMARCE1*, *NF2* and *BAP1* alterations. Among *NF2*-altered tumors, patterns of Merlin inactivation, shaped by germline status and co-occurring copy-number variations, delineate biologically divergent subsets. In patients ≤21 years, extent of resection is the dominant predictor of outcome, while molecular features further refine risk assessment. These findings define pediatric and young adult meningiomas as a distinct molecular entity and support age-adapted risk refinement that integrates molecular features with strong clinical determinants.

## Abstract (web summary)

Pediatric and adolescent/young adult meningiomas remain poorly defined biologically and clinically, and risk stratification is still largely based on adult tumors. Here, the authors profile 293 tumors from patients aged 0–39 years and show that especially meningiomas arising in patients aged ≤21 years represent a particularly distinct molecular and clinical subgroup requiring age-adapted risk stratification.

## Introduction

Meningiomas, the most common primary intracranial tumors in adults, also arise across childhood, adolescence and young adulthood, spanning an age range in which incidence is low in early life and increases steadily toward adulthood^1^. This continuous developmental window, encompassing the full spectrum from early life to young adulthood (0–39 years), suggests that differences in meningeal maturation, cellular composition and microenvironmental cues may influence tumor molecular drivers and pathological behavior, and may therefore not fully conform to adult-based diagnostic and prognostic criteria^2–5^.

In adults, several stratification frameworks have been developed and validated, integrating histopathology, molecular alterations and DNA methylation profiling to classify meningiomas into subgroups that correlate with recurrence risk and clinical outcomes^5,6^. These systems extend beyond classical WHO grading, incorporating molecular features such as DNA methylation class, and have stratified tumors into categories including benign, intermediate and malignant, or into Merlin-intact, immune-enriched and hypermitotic subtypes^7–10^. Despite differences in nomenclature and the number of subgroups, they consistently identify high-risk *NF2*-mutant tumors, lower-risk *NF2*-mutant tumors and *NF2* wild-type tumors^7–10^. Emerging evidence indicates that the tumor microenvironment is a key determinant distinguishing high- and low-risk groups and likely modulates progression along the malignancy spectrum^8,11,12^. However, the applicability of these frameworks to pediatric and young-onset meningiomas remains largely unexplored, as comprehensive methylation and molecular profiling data are limited for tumors from younger patients^4,5^.

Pediatric and young-onset meningiomas are frequently associated with germline tumor-predisposition syndromes, including *NF2*-related meningiomas^13^, *SMARCE1*-associated clear-cell meningiomas^14,15^ and *BAP1* tumor-predisposition syndrome^16,17^, highlighting the importance of comprehensive genetic and radiologic evaluation^4^. Molecularly, youth-onset tumors are dominated by alterations in *NF2*^3,18,19^ and *SMARCE1*^3,20^, with rarer oncogenic *YAP1* fusions (including *YAP1::MAML2*, *YAP1::PYGO1*, and *YAP1::LMO1*) that have been proposed as alternative oncogenic drivers to NF2 inactivation^21,22^. In contrast, canonical adult driver mutations, including *AKT1*, *KLF4*, *TRAF7* and *SMO*, are largely absent^3,19^.

Adult studies have shown that integrating WHO grade, methylation class and high-risk copy-number variations (CNVs), including chromosome (chr) 1p, 6q and 14q losses, accurately predicts recurrence, with chr1p loss representing an early high-risk event^5,23^. Whether these markers hold prognostic significance in younger patients remains unclear and it is unknown whether youth-onset meningiomas represent a continuum of adult biology or a distinct molecular entity.

Despite emerging investigations into age-associated molecular features, comprehensive analyses integrating histopathology, genomics, epigenomics and clinical outcomes across the pediatric-to-young adulthood continuum remain scarce. Systematic characterization of this population provides an opportunity to define features shared with adult disease and those unique to youth-onset tumors, advancing understanding of meningioma formation and progression.

Here, we define the histopathologic, molecular and clinical landscape of meningiomas in 293 patients aged 0–39 years, including *n* = 212 0–21-year-old patients and *n* = 81 22–39-year-old patients. By integrating morphology, methylation class, copy-number alterations and targeted sequencing, we map age-associated patterns to delineate the continuum of meningioma biology from early life through young adulthood.

## Results

### Age-associated differences in clinical and histopathological features of meningiomas

**Fig. 1:**
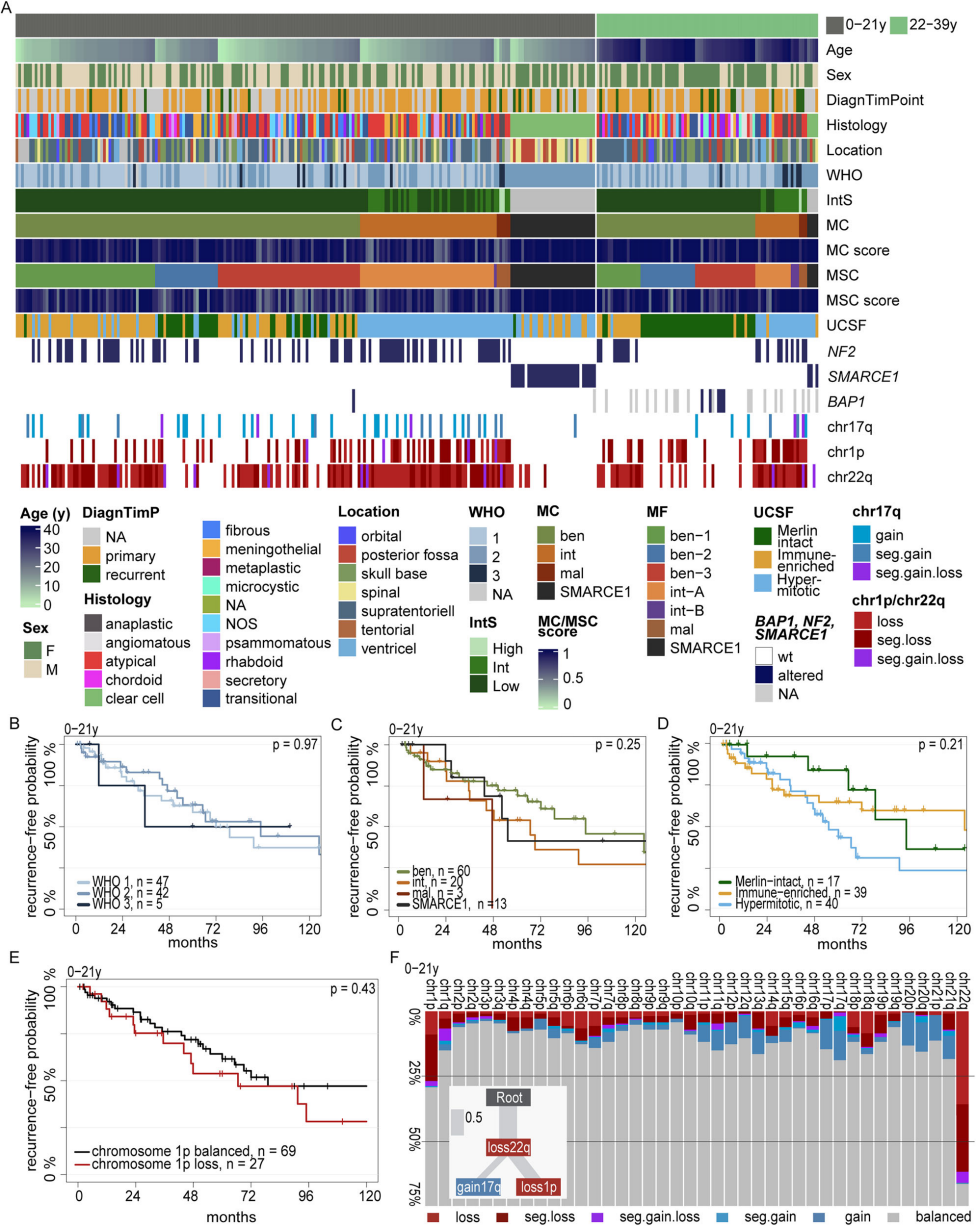
Clinicopathological and molecular landscape of pediatric and young-onset meningiomas. **A** Oncoprint representation of 293 meningioma samples from patients 0–39 years of age. y year, DiagnTimePoint Timepoint of Diagnosis, IntS Integrated Risk Score system proposed by Maas et al.^23^, MC methylation class by Sill et al.^26^, MSC methylation subclass, F female, M male, NA not available, NOS not otherwise specified, ben benign, int intermediate, mal malignant, UCSF meningioma classification system proposed by Choudhury et al.^8^, chr chromosome, seg segmental, wt wildtype. **B** Kaplan-Meier analysis of risk of progression of the subgroup of patients 0–21 years of age, stratified according to WHO grade (WHO grade 1, *n* = 47; WHO grade 2, *n* = 42; WHO grade 3, *n* = 5), **C** DNA methylation class (ben, *n* = 60; int, *n* = 20; mal, *n* = 3; SMARCE1, *n* = 13), **D** and UCSF class (Merlin-intact, *n* = 17; Immune-enriched, *n* = 39; hypermitotic, *n* = 40). **E** Kaplan-Meier analysis of risk of progression for patient 0–21 years of age, stratified according to the presence (*n* = 27) or absence (*n* = 69) of a chromosome 1p loss. The p-value was calculated using the Log-rank test. y years. **F** Overview of chromosomal alterations in the 0–21 years of age group (*n* = 212), plotted across individual chromosomal arms. For the oncogenetic trees, only gains/losses with at least 10% prevalence were used and only the first two nodes are shown. seg. segmental, chr chromosome, y years. Source data are provided as a Source Data file.

The cohort comprised 293 meningioma samples from individual patients younger than 40 years of age (Fig. 1A, Supplementary Table 1). For subgroup analyses, a cut-off was set at 21 years, as commonly used in the literature (0–21 years: *n* = 212, mean age: 12.8 years, SD: 5.0 years; 22–39 years: *n* = 81, mean age: 33.3 years, SD: 4.7 years). Analyses were performed across the full cohort with stratified evaluation of both age groups to capture potential biological differences and their transition toward adult-type disease. In the adult comparison cohort, consisting of already published data from patients >39 years (*n* = 747)^23^, the median age was 60.8 years (SD: 11.3 years).

As previously described^2,24,25^, age-related differences in the female-to-male ratios were observed: the 0﻿–21-year-old group showed a female-to-male ratio of 0.9, which increased to 3.1 in the 22–39-year-old cohort and to 2.1 in the adult comparison cohort (0–21 years vs. 22–39 years, *p* = 0.006; 0–21 years vs. >39 years, *p* < 0.001).

Concerning the histological subtypes, meningiomas from patients ≤39 years showed a higher percentage of the clear cell subtype than their adult counterparts (0–21 years: 15.2%; >39 years: 1.4%, *p* < 0.001; 22–39 years: 4.9%, *p* = 0.016). Further, the 0﻿–21-year-old group showed the anaplastic subtype less frequently than meningiomas from adults (0–21 years: 3.4%; 22–39 years: 6.2%; >39 years: 9.6%, *p* = 0.004).

Regarding tumor location, 0﻿–21-year-old patients showed a higher frequency of spinal meningiomas than 22﻿–39-year-old patients (13.4% vs. 2.6%, *p* = 0.01). Both patient groups (0﻿–21 and 22–39 years) showed a notable frequency of intraventricular (0﻿–21 years: 6.4%; 22–39 years: 5.3%) and orbital (0–21 years: 4.7%; 22﻿–39 years: 3.9%) meningiomas, consistent with prior reports^2,24^ (Fig. 1A, Supplementary Table 1).

Immunohistochemistry (IHC) for progesterone (PR) and estrogen (ER) receptors was performed for 52 meningiomas from patients aged 0﻿–21 years. ER status was consistently negative or not evaluable. For PR, a significantly higher H-score was detected for meningiomas from females than from males (Supplementary Fig 1A).

### Age-dependent shifts in WHO grades and DNA methylation-based meningioma classes

No significant differences in WHO grading were observed between tumors from 0﻿–21 (WHO 1: 50.5%, WHO 2: 45.1%, WHO 3: 4.4%) and 22–39-year-old patients (WHO 1: 49.4%, WHO 2: 43.2%, WHO 3: 7.4%). However, meningiomas from 0﻿–21-year-old patients were less frequently classified as WHO grade 3 than meningiomas from adults ( >39 years: 8.5%, *p* = 0.053) (Fig. 1A, Supplementary Table 1).

As an additional, more granular classification system, the distribution of Heidelberg DNA methylation classes (MC) and subclasses (MSC) was analyzed (Fig. 1A, Supplementary Table 1)^7,26^. Regarding MCs, only the SMARCE1 class showed a significant difference, with a higher prevalence among meningiomas from 0﻿–21-year-old patients (0﻿–21 years: 14.5%; 22﻿–39 years: 4.9%, *p* = 0.026; >39 years: 1.1%, *p* < 0.001). Concerning MSCs, the proportion of ben-2-classified tumors increased with age (0–21 years: 10.8%; 22–39 years: 24.7%; >39 years: 27.0%; 0﻿–21 vs. 22–39 years, *p* = 0.005; 0–21 years vs. >39 years, *p* < 0.001), whereas the proportion of ben-3-classified tumors decreased (0–21 years: 24.5%; 22﻿–39 years: 27.2%; >39 years: 14.9%; 0–21 years vs. >39 years, *p* < 0.001).

Further, the distribution within the classification system proposed by Choudhury et al. (UCSF classes)^8^ was evaluated (Fig. 1A, Supplementary Table 1). Here, meningiomas from 0﻿–21-year-old patients were more frequently classified as hypermitotic (0﻿–21 years: 43.9%; 22–39 years: 27.2%, *p* = 0.006; >39 years: 33.1%, *p* = 0.004) and immune enriched (0–21 years: 38.2%; 22–39 years: 21.0%, *p* = 0.006; >39 years: 24.4%, *p* < 0.001) and less frequently as Merlin-intact (0–21 years: 17.9%; 22–39 years: 51.9%, *p* < 0.001; >39 years: 42.5%, *p* < 0.001).

### Established adult risk stratification systems lack prognostic value in younger patients

Next, the classification systems were evaluated for their ability to provide prognostic stratification. For 165 of 293 patients (0﻿–39 years), outcome data was available. For the 0–21-year-old group, no significant differences in progression-free survival (PFS) were detected for either WHO grades (Fig. 1B), MC (Fig. 1C) or UCSF classes (Fig. 1D), and hazard ratios were considerably smaller (Supplementary Fig. 4). In contrast, in the 22–39-year-old group, all systems provided a significant stratification (Supplementary Fig. 1B), with WHO grade being the best discriminating (highest c-index) risk factor (Supplementary Fig. 4B). Although only minor differences in malignancy grading exist between the age groups, its relevance is limited, as the grading systems appear to have limited prognostic value in the patient group from 0﻿–21 years.

Beyond DNA methylation classification, CNVs have gained increasing importance for risk stratification in meningiomas^5,23,27–29^. In particular, loss of chr1p is recognized as an early event with a significant impact on tumor malignancy^5,28^, and 28.8% of meningiomas from 0﻿–21-year-old patients and 40.7% of meningiomas from 22﻿–39-year-old patients exhibited this CNV (*p* = 0.068). However, it was not associated with adverse prognostic outcome (Fig. 1E, Supplementary Fig. 1C). Interestingly, the meningioma integrated risk score (IntS) did not achieve significant prognostic stratification in the patient groups 0﻿–21 and 22–39 years of age (Supplementary Fig. 1D).

In contrast to meningiomas from adults, the CNV profiles of meningiomas from younger patients were predominantly characterized by chromosomal gains (Fig. 1F and Supplementary Fig. 1E). This pattern is also reflected in the oncogenetic trees: While in the adult comparison cohort the loss of chr22q was followed by loss of chr1p, the oncogenetic tree for the 0﻿–21 years of age group showed an additional gain of chr17q at this position (18.4%, >39 years: 9.8%, *p* < 0.001), for the 22–39 years of age group a loss of chr8p or chr8q was indicated (Supplementary Fig. 2A–C).

### Patient age influences DNA methylation clustering of meningiomas and cellular composition

**Fig. 2:**
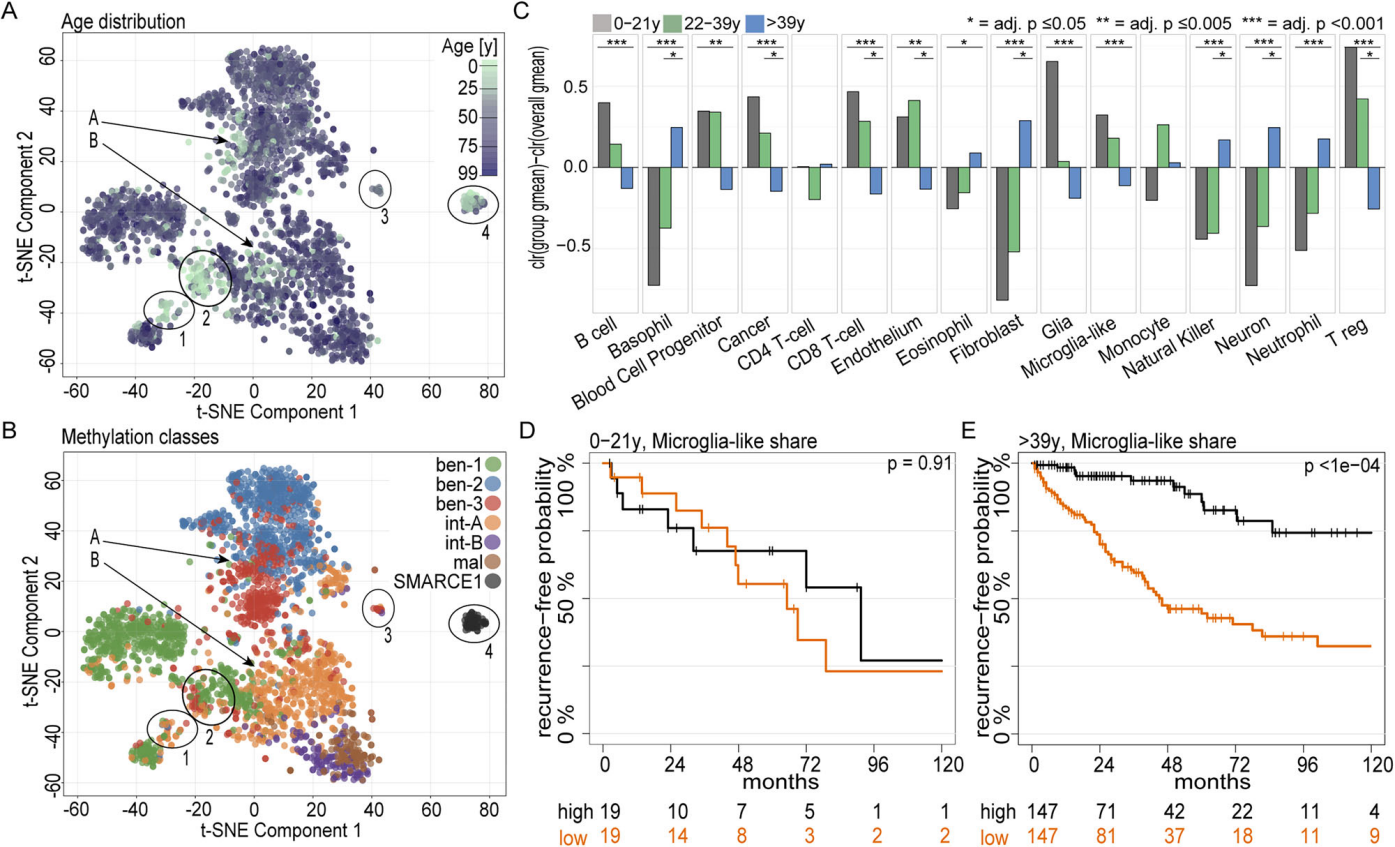
Patient age shapes the DNA methylation landscape and cellular composition of meningiomas. **A** t-SNE projection of DNA methylation profiles (20 perplexity, 1000 iterations, distance metric: weighted Pearson) including the cases of this study together with 2197 meningiomas retrieved from the database of the Neuropathology Department in Heidelberg with available information on age and sex of the patients and a DNA methylation family score for meningioma >0.9. Plots were colored based on the age of the patient (5-year steps were used for coloring) and **B** the DNA methylation subclasses. The pediatric and young-onset clusters are circled in black and termed 1–4. Two regions enriched for tumors from younger patients are indicated with a black arrow and termed (A) and (B). **C** Compositional data analysis plot based on bulk methylation deconvolution data for meningiomas from patients 0–21 years (*n* = 212), 22–39 years (*n* = 81) and adults ( >39 years, *n* = 747). The ‘adj p’ gives the p-value adjusted for multiple testing controlling the false discovery rate. Groups are compared with a t-test using the first Pivot log-ratio coordinate of components. clr centered log ratio. Exact p-values are provided in Supplementary Table 2. **D** Kaplan-Meier analysis of risk of progression of the subgroup of patients 0–21 years and **E** over 39 years of age stratified into top 20% and bottom 20% groups based on microglia-like cell proportion (0–21 years, *n* = 19 per group; >39 years, *n* = 147 per group). y years. The p-values were calculated using the Log-rank test. Source data are provided as a Source Data file.

Results from histology, DNA methylation-based classification and CNV analysis indicate that meningiomas arising in younger patients, especially up to 21 years, represent a distinct molecular entity. This was also supported by visualizing DNA methylation profiles of tumors from patients 0–39 years of age together with ~2200 meningiomas by *t*-SNE analysis. Here, tumors from patients 0–39 years of age, along with younger comparison samples, formed four separate clusters, including the known *SMARCE1*-associated cluster (cluster 4)^20^ and were enriched in two distinct areas (termed A and B). However, a small subset of pediatric and young-onset tumors clustered together with adult tumors (Fig. 2A, B).

In adult meningiomas, the tumor microenvironment (TME) is a major determinant of methylation-based clustering, and in particular the proportion of microglia-like cells within the tumor is associated with differences in PFS^8,11,30^. To determine whether TME composition differs across age groups, bulk DNA methylation data were deconvoluted. In direct comparison between the 0﻿–21-year-old and 22–39-year-old groups, no significant differences were detected for any of the individual cellular components across the complete cohorts and even when comparing age groups within the MC (Fig. 2C, Supplementary Table 2, 3). Also, when fitting a multivariable linear model for each TME component and including age and MC or driver status as covariates, there was no significant difference in the comparison between the 0﻿–21 and 22–39-years-of-age groups (Supplementary Fig. 3A). This finding was exemplarily validated for CD68-positive cells, including macrophages and microglia, using IHC, and likewise no significant difference was detected (Supplementary Fig. 3B). In contrast, both groups showed pronounced differences in their cellular composition when compared with adult meningiomas.

Tumors from younger patients showed a significantly higher share of tumor cells than meningiomas from adults (0﻿–21 years: *p* < 0.001; 22﻿–39 years: *p =* 0.028). The most prominent differences observed in both groups concerned regulatory T cells, which were present at a substantially higher proportion in tumors from younger patients (0–21 years: *p* < 0.001; 22–39 years: *p* = 0.03) and fibroblasts, which showed a higher share in meningiomas from adults (0–21 years: *p* < 0.001; 22–39 years: *p* = 0.02).

For microglia-like cells, tumors from younger patients showed a higher proportion compared with adult meningiomas; however, this difference reached statistical significance only for the 0–21 years of age group (*p* < 0.001). Finally, stratifying tumors from 0﻿–21-year-old patients into the top and bottom 20% based on microglia-like cell proportions, estimated by deconvolution, did not reveal differences in PFS (Fig. 2D, 22–39 years Supplementary Fig. 3C). This contrasts with the adult comparison cohort cases, in which high versus low microglia-like cell content shows clear associations with clinical outcomes (Fig. 2E)^12^.

### Driver alterations influence the epigenetic clustering

Known high-risk alterations such as homozygous *CDKN2A/B* deletions (*n* = 1, age: 36 years) and *TERT* promotor mutations (n = 2, age: 33 and 38 years*)* were absent in meningiomas from 0﻿–21-year-old patients. In contrast, *SMARCE1* alterations were particularly enriched in meningiomas from younger patients (0–21 years: 13.7%; 22–39 years: 3.7%, *p* = 0.012). Meningiomas from 22﻿–39-year-old patients showed a higher frequency of *BAP1* alterations (8.1%; 0–21 years: 0.6%, *p* = 0.005), which is consistent with the previously reported median age at diagnosis of 44 years for patients with *BAP1*-altered meningiomas^31^. *YAP1*-fusions have also been described as a potential driver for pediatric meningiomas^21^; of the five cases with detectable *YAP1*-fusion, all fell within the 0–21 years of age group. *NF2* alterations, including mutations, fusions, and homozygous deletions detected in the sequencing data, were frequently observed across the cohorts, with prevalence rates of 37.3% (0–21 years), 23.5% (22–39 years) and 33.5% (>39 years). When including cases with a chr22q loss, the percentages increased to 67% in the 0﻿–21 years and 53.1% in the 22–39 years of age groups.

**Fig. 3:**
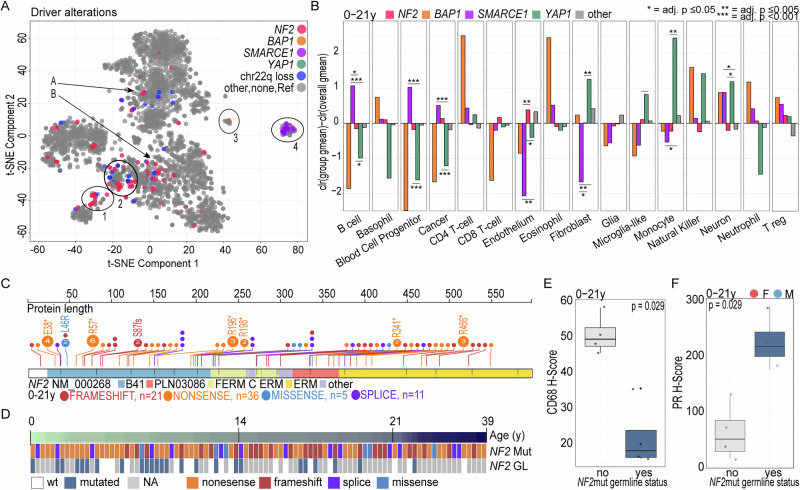
Driver alterations define epigenetic clustering and tumor composition in pediatric and young-onset meningiomas. **A** Methylation clustering displayed as a t-SNE projection (20 perplexity, 100 iterations, distance metric: weighted Pearson) of the cases of this study with 2,197 meningiomas retrieved from the database of the Neuropathology Department in Heidelberg with available information on age and sex of the patients and a methylation family score for meningioma >0.9. Cases were colored based on detected alterations in *NF2*, *SMARCE1*, *BAP1* or *YAP1*-fusion and a loss of chromosome 22q. The pediatric and young-onset clusters are circled in black and termed 1–4. Two regions enriched for tumors from younger patients are indicated with an arrow and termed (A) and (B). **B** Compositional data analysis plot based on bulk methylation deconvolution data for meningiomas from patients 0﻿–21 years, stratified according to their driver mutation. The “other” subgroup also contains meningiomas with unknown *BAP1* status. The ‘adj p’ gives the p-value adjusted for multiple testing, controlling the false discovery rate. Groups are compared with a t-test using the first Pivot log-ratio coordinate of components. clr: centered log ratio. Exact p-values are provided in Supplementary Table 4. *NF2*-altered group, *n* = 79; *BAP1*-altered group, *n* = 1; *SMARCE1*-altered group, *n* = 29; *YAP1*-fused group, *n* = 5; other group, *n* = 99. **C** Visualization of the *NF2* mutation profile in the cohort of patients 0﻿–21 years of age (*n* = 73). The plot was created using the online tool ProteinPaint (available at https://proteinpaint.stjude.org). **D** Overview of the type of *NF2* mutation and germline status across patients aged 0–39 years. Mut mutation, GL germline, wt wildtype, na no information available. **E** Boxplot comparing MIKAYA-derived CD68 H-scores between patients 0﻿–21 years of age presenting with an *NF2*-mutated meningioma and stratified according to the presence of a *NF2* germline mutation (no: *n* = 4, yes: *n* = 4). The p-value was calculated using the Wilcoxon rank sum test (two-sided). Horizontal lines of the box indicate Q1, median (bold) and Q3 of the distribution; whiskers extend to the most extreme data point, which is no more than 1.5*IQR from the box. *NF2*mut *NF2* mutation. **F** Boxplot comparing CD68 and progesterone receptor (PR) H-scores between patients 0–21 years of age, presenting with a *NF2-*mutated meningioma, stratified according to the presence of a *NF2* germline mutation (no: *n* = 4, yes: *n* = 3). *NF2*mut *NF2* mutation, PR progesterone receptor, F female, M male. The p-value was calculated using the Wilcoxon rank sum test (two-sided). Horizontal lines of the box indicate Q1, median (bold) and Q3 of the distribution; whiskers extend to the most extreme data point, which is no more than 1.5*IQR from the box. Source data are provided as a Source Data file.

To examine whether the driver alterations represent an additional factor influencing the DNA methylation clustering beyond patient age itself, tumors were re-annotated according to mutation status (Fig. 3A): Cluster 4 consisted of *SMARCE1*-altered meningiomas and cluster 3 contained the *BAP1*-altered meningiomas. Both clusters have already been described and were found to be significantly associated with younger patients^20,31^.

### Driver alterations shape tumor composition

Driver alterations shaped the cellular composition of meningiomas from patients 0﻿–21 years of age: Deconvolution analysis revealed that *SMARCE1-* and *NF2-*altered tumors harbored a significantly higher fraction of tumor cells compared with *YAP1-*fusion–positive meningiomas (both, *p* < 0.001). Overall, tumors with *SMARCE1* and *YAP1* alterations, as well as *SMARCE1-* versus *NF2-*altered tumors, demonstrated significant differences in their cellular composition (both, *p* < 0.001, Fig. 3B). This also holds true when fitting a multivariable model (Supplementary Fig. 3E) and when comparing adult cases with either a *NF2* or *SMARCE1* alteration (*p* < 0.001) - indicating a driver-specific rather than an age-specific effect (Supplementary Table 4 and 5).

However, a significant difference in microglia-like cell content was observed only in the comparison between *SMARCE1-* and *YAP1-*altered tumors (*p* = 0.004). Although deconvolution did not detect differences between *NF2-* and *SMARCE1-*altered tumors, these were indicated by the CD68 IHC stainings (Supplementary Table 4, Supplementary Fig. 3E). Interestingly, *NF2*-altered meningiomas also exhibited higher PR H-scores compared to *SMARCE1-*altered tumors (Supplementary Fig. 3F).

Given the high prevalence of *NF2*-altered meningiomas in younger patients, we examined this subgroup in detail. Several mutation hotspots, such as p.R57*, were detected across all age groups and the overall mutation spectrum did not differ between the patient groups 0–21 and 22–39 years of age. Further, *NF2*-mutated versus *NF2*-wild-type tumors (excluding *SMARCE1-* and *BAP1*-altered tumors) did not show significant differences in PFS (Supplementary Fig. 3G). Likewise, among *NF2*-mutated tumors, the presence or absence of a germline *NF2* mutation had no significant impact on PFS (Supplementary Fig. 3H). The *NF2-*germline-mutated tumors also did not cluster together in the t-SNE projection (Supplementary Fig. 3I). Despite this, differences in the IHC were observed: *NF2* germline-mutated tumors showed significantly lower CD68 H-scores than non-germline *NF2*-mutated tumors, but demonstrated a significantly higher H-score in the PR receptor staining (Fig. 3F, G). However, due to the limited number of cases, separate sex-stratified statistical analyses could not be performed.

### Distinct prognostic patterns in *NF2*-mutated versus other meningioma subgroups

**Fig. 4:**
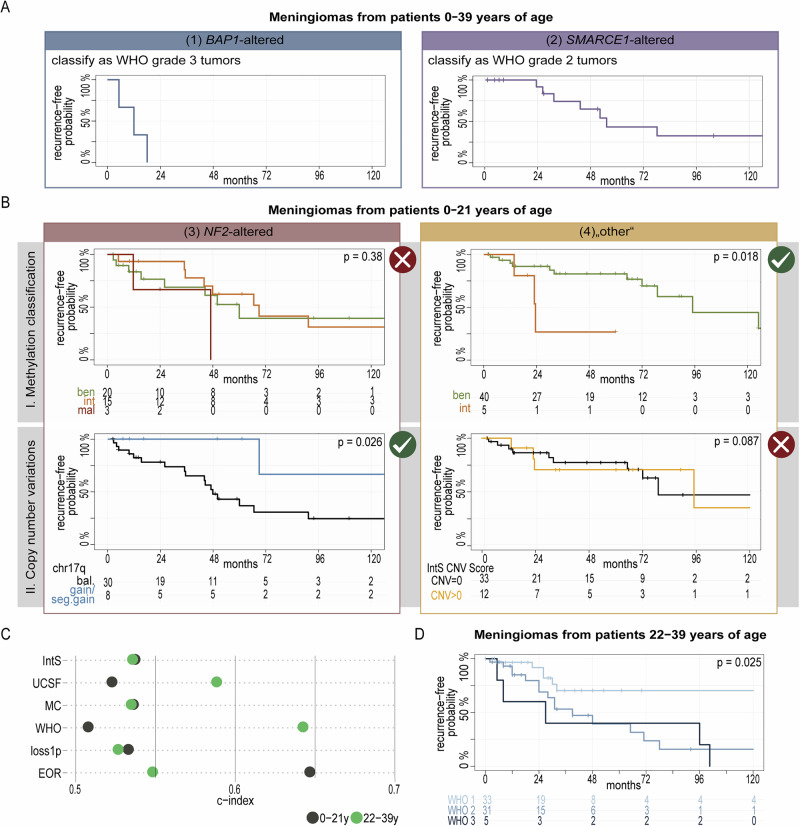
Prognostic stratification identifies distinct molecular subgroups in pediatric and young-onset meningiomas. **A** (1) *BAP1*-altered meningiomas showed a highly aggressive clinical behavior and should be classified as WHO grade 3 (Sievers et al.^31^). The Kaplan-Meier analysis included all cases from 0–39 years (*n* = 3). (2) *SMARCE1*-altered meningiomas, represented a distinct molecular entity and should be classified as WHO grade 2 (*n* = 15; Sievers et al.^20^). The Kaplan–Meier analysis included patients from 0–39 years. **B** Patient group 0﻿–21 years of age: (3) *NF2-*altered meningiomas; and (4) tumors not falling into these categories, classified as “other”. The “other” subgroup also contained meningiomas with unknown *BAP1* status. The Kaplan-Meier analyses of the progression risk for the *NF2-*altered and “other” subgroups of patients 0–21 years of age, stratified by I. methylation class, ben benign, int intermediate, mal malignant (NF2-altered: ben, *n* = 20; int, *n* = 15; mal, *n* = 3; other: ben, *n* = 40; int, *n* = 5), II. by copy-number alterations: chromosome 17q gain for the *NF2-*altered subgroup (balanced *n* = 30; gain/seg.gain, *n* = 8) and the presence of high-risk copy number variations as defined by the Integrated Risk Score for meningiomas for the “other” subgroup (Score 0, *n* = 33; Score >0, *n* = 12). No adjustment for multiple testing was applied. chr chromosome, bal. balanced, seg.gain segmental gain, IntS Integrated risk score, CNV copy number variation. The p-values were calculated using the Log-rank test. **C** Comparison of the c-index among the patient groups 0–21 years of age and 22–39 years of age across different stratification systems. IntS Integrated meningioma risk score by Maas et al.^23^ 0–21 years: low, *n* = 57; int, *n* = 19; high, *n* = 5; 22–39 years: low, *n* = 46; int, *n* = 13; high, *n* = 7; UCSF UCSF methylation classes by Choudhury et al.^8^ 0–21 years: Merlin-intact, *n* = 17; Immune-enriched, *n* = 39; Hypermitotic, *n* = 40; 22–39 years: Merlin-intact, *n* = 36; Immune-enriched, *n* = 15; Hypermitotic, *n* = 18; MC Heidelberg methylation classes by Sill et al.^26^ 0–21 years: ben, *n* = 60; int, *n* = 20; mal, *n* = 3; SMARCE1, *n* = 13; 22﻿–39 years: ben, *n* = 49; int, *n* = 15; mal, *n* = 2; SMARCE1, *n* = 3; WHO WHO grades 0–21 years: WHO grade 1, *n* = 47; WHO grade 2, *n* = 42; WHO grade 3, *n* = 5; 22–39 years: WHO grade 1, *n* = 33; WHO grade 2, *n* = 31; WHO grade 3, *n* = 5; loss1p loss of chromosome 1p; 0–21 years: 1p balanced, *n* = 69; 1p loss, *n* = 27; 22–39 years: 1p balanced, *n* = 42; 1p loss, *n* = 27; EOR Extent of resection based on Simpson grading 0–21 years: grade 1–3, *n* = 50; grade 4–5, *n* = 24; 22–39 years: grade 1–3, *n* = 47; grade 4–5, *n* = 11. **D** Kaplan-Meier analysis of risk of progression of the subgroup of patients 22–39 years, stratified according to WHO grade (WHO grade 1, *n* = 33; WHO grade 2, *n* = 31; WHO grade 3, *n* = 5). The p-values were calculated using the Log-rank test. Source data are provided as a Source Data file.

*SMARCE1*- and *BAP1*-altered meningiomas are well-established as distinct subtypes across patients aged 0–39 years^20,31^ (Fig. 4A); however, especially in the age group of 0–21 years, two additional distinct molecular subgroups emerged (Fig. 4B). In a new stratification approach, the prognostic relevance of various markers was analyzed between the *NF2*-mutated group and non-*NF2*, non-*BAP1* and non-*SMARCE1* altered meningiomas (“other”) in the 0–21 years of age group (Fig. 4B). Tumors showing a chr22q loss without a *NF2*-alteration (*n* = 10) were assigned to the “other” subgroup. All of these tumors were classified as MC benign, and statistical analyses showed that the effect of CNVss is limited to cases from the *NF2-*altered group (Supplementary Fig. 4D).

In this analysis, “other” meningiomas could be stratified according to their DNA methylation class and showed a significant difference in PFS between cases classified as benign and intermediate. In contrast, within the *NF2*-mutant group, the DNA methylation-based classification did not allow for prognostic differentiation. In both groups, chr1p status was not indicative of poor survival. However, in the *NF2*-altered cohort, a gain of chr17q was associated with better survival. For the “other” group, no known high-risk CNVs and alterations of chr17q significantly affected PFS.

### Surgical extent as primary prognostic factor within an age-adapted stratification framework

**Fig. 5:**
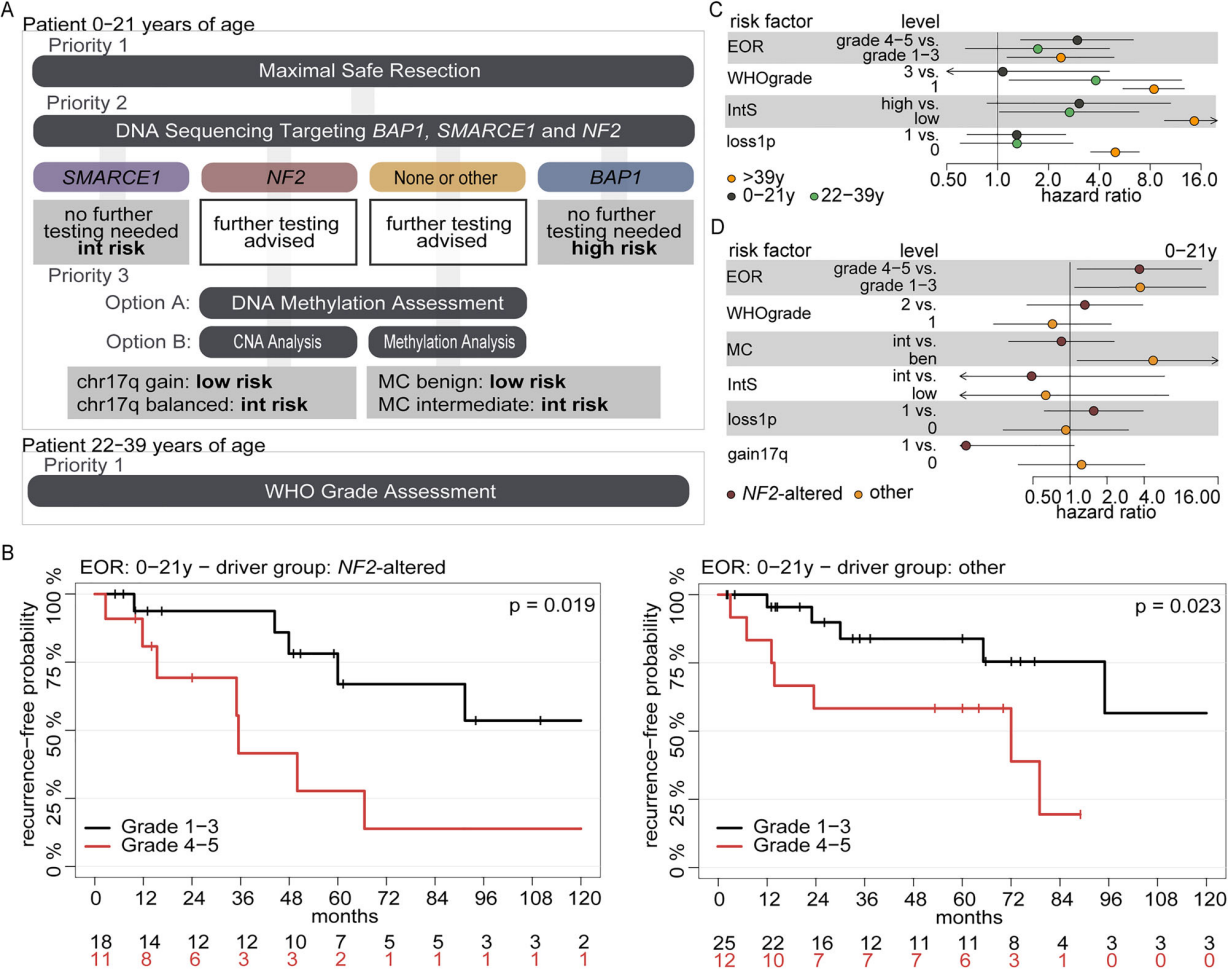
Hierachical age-adapted risk stratification for pediatric and young-onset meningiomas. **A** Workflow for efficient meningioma evaluation in patients aged 0–21 years in diagnostic routine. Maximal safe resection remains the highest priority for reducing recurrence risk and detailed documentation of the extent of resection is essential for accurately assessing and defining the individual risk of recurrence. As a second priority, DNA sequencing should be performed to detect alterations in *SMARCE1, BAP1* and *NF2*. Meningiomas harboring *SMARCE1* or *BAP1* alterations do not require additional molecular testing and can be stratified as intermediate-risk and high-risk for recurrence, respectively. For *NF2*-altered meningiomas and meningiomas without alterations in *SMARCE1, BAP1* or *NF2*, further molecular workup can follow two alternative paths: Option A (feasible for both subgroups): Methylation data can be generated using, for example, nanopore sequencing or methylation array profiling, allowing the simultaneous assessment of copy-number variation and methylation class. Option B (subgroup-specific): For *NF2*-altered cases, targeted analysis of chromosome 17q copy-number status (e.g., via FISH) can be performed, while for *NF2*-wild-type / “other” cases, methylation profiling alone is sufficient to enable subsequent stratification into low- and intermediate-risk groups. CNA copy number alteration, in intermediate, MC methylation class, chr chromosome. For patients aged 22–39 years, the WHO grading has the highest priority. **B** Kaplan-Meier analysis for the patient group 0–21 years of age for the driver groups “*NF2*-altered” and “other”, stratified for extent of resection Simpson grades 13 and 4–5. EOR extent of resection, y years, The p-values were calculated using the Log-rank test. **C** Summary of risk factors and their corresponding hazard ratios for the age groups 0–21 years, 22–39 years and >39 years. Horizontal lines indicate the 95% confidence interval of the hazard ratio. EOR extent of resection based on Simpson grading (0–21 years: grade 4–5, *n* = 24; grade 1–3, *n* = 50; 21–39 years: grade 4–5, *n* = 11; grade 1–3, *n* = 43; >39 years: grade 4–5, *n* = 23; grade 1–3, *n* = 335), IntS Integrated meningioma risk score by Maas et al.^23^ (0–21 years: high, *n* = 4; low, *n* = 59; 21–39 years: high, *n* = 7; low, *n* = 46; >39 years: low, *n* = 392; high, *n* = 112), WHO grade: 0–21 years: grade 3, *n* = 5; grade 1, *n* = 47; 21–39 years: grade 1, *n* = 33; grade 3, *n* = 5; >39 years: grade 1, *n* = 394; grade 3, *n* = 61; loss1p: 0–21 years; balanced, *n* = 69; loss/seg.loss, *n* = 27; 21–39 years; balanced, *n* = 42; loss/seg.loss, *n* = 27; >39 years; balanced, *n* = 440; loss/seg.loss, *n* = 290) y years. **D** Summary of risk factors and their corresponding hazard ratios in *NF2-*altered meningiomas and “other” meningioma (0–21 years). The “other” subgroup also contains meningiomas with unknown *BAP1* status. Horizontal lines indicate the 95% confidence interval of the hazard ratio. EOR extent of resection based on Simpson grading, MC methylation class, ben benign, int intermediate, mal malignant, IntS Integrated meningioma risk score, y years. *NF2*-altered group: WHO grade 2, *n* = 12; WHO grade 1, *n* = 21; EOR grade 4–5, *n* = 11; grade 1–3, *n* = 18; MC int, *n* = 18; ben, *n* = 20; IntS int, *n* = 8; low, *n* = 24; loss1p balanced, *n* = 23; loss/seg.loss, *n* = 15; chr17q balanced *n* = 30, gain/seg.gain *n* = 8. “Other” group: WHO grade 2 *n* = 17; WHO grade 1, *n* = 26; EOR grade 4–5, *n* = 12; grade 1–3, *n* = 25; MC int, *n* = 5; ben, *n* = 40; IntS int, *n* = 10; low *n* = 35; loss1p balanced, *n* = 33; loss/seg.loss, *n* = 12; chr17q balanced, *n* = 36; gain/seg.gain, *n* = 9. Source data are provided as a Source Data file.

Integrating clinical, genomic and epigenetic features demonstrated that meningiomas from younger patients constitute biologically distinct subgroups. Especially patients 0–21 years of age benefit from a hierarchical, age-adapted stratification approach rather than uniform application of adult-derived risk schemes (Fig. 5A).

For patients 0–21 years of age, the extent of resection (EOR) emerged as the strongest and most consistent predictor of outcome. EOR displayed the largest hazard effect across all molecular subgroups and had the overall highest c-index, reinforcing prior pediatric observations and underscoring its central role in determining recurrence risk in this age group, while complementary molecular features refine prognostic resolution within the driver-defined subgroups (Fig. 4C, Fig. 5B–D and Supplementary Fig. 4B–D).

Besides the EOR, radiotherapy is an important prognostic factor; however, detailed information on its timing and intent was inconsistently available (supplementary table 6). Patients receiving adjuvant radiotherapy more frequently had unfavorable tumor characteristics, including higher WHO grade and Simpson grade, indicating confounding by indication. After adjustment for these factors and diagnosis timepoint, adjuvant radiotherapy remained associated with a non-significant increase in risk in both multivariable and propensity score–weighted analyses (Supplementary Fig. 4A). This likely reflects residual confounding and selection bias rather than a causal effect.

### Integrated, hierarchical risk stratification reveals distinct prognostic subgroups in pediatric and AYA meningiomas

Molecular drivers provide the most informative second tier of refinement. Four principal driver-defined groups (*NF2*-altered, *SMARCE1*-altered, *BAP1*-altered and driver negative or other driver events) displayed clear biological separation. *SMARCE1*-altered tumors, consistent with previous reports^20^, aligned with an intermediate risk profile, whereas *BAP1*-altered tumors demonstrated uniformly aggressive behavior corresponding to a high-risk category. These two entities behaved independently from both *NF2-*driven tumors and “driver-negative” tumors, supporting their early and separate allocation within a young adult-specific risk model.

Within the large *NF2*-altered subgroup, additional refinement was necessary to account for its underlying genomic diversity. Integration of selective CNV markers, particularly the presence or absence of chr17q gain, proved informative. These features identified biological subdivisions with differential behavior that were not captured by driver mutation status alone, highlighting the relevance of *NF2* context-specific genomic architecture in younger patients. Conversely, tumors lacking *NF2, SMARCE1, or BAP1* alterations formed a heterogeneous group that, in the presence of a chr22q loss, benefited from epigenomic stratification. In this subgroup, DNA methylation-based subclassification remained prognostically meaningful despite the underlying genomic diversity, providing a consistent framework for distinguishing lower from higher risk disease. When applying the risk stratification displayed in Fig. 5A to the presented cohort of 0–21 years of age patients, only low (*n* = 48) and intermediate (*n* = 48) cases can be compared since there are no *BAP1*-altered cases in this group. In a multivariable Cox PH model including risk stratification, EOR, WHO grade and sex, only intermediate risk (HR: 2.5, 95%CI 1.2-5.2, *p* = 0.02) and EOR (Simpson grade 4–5, HR: 2.9, 95%CI 1.3-6.1, *p* = 0.001) remained significant risk factors (Supplementary Table 7).

Taken together, these findings support a hierarchical, subgroup-specific risk stratification strategy for meningiomas from patients 0–21 years of age. This framework begins with EOR, followed by driver mutation status, and is selectively refined by DNA methylation and copy-number data where appropriate. Such an approach reflects the unique biological architecture of this age group and addresses the limitations inherent in applying adult-derived risk models to pediatric and young adult patients.

## Discussion

In this study, we characterize the molecular and clinical landscape of meningiomas arising in patients aged 0–39 years, revealing characteristic biological features that distinguish these tumors from adult disease and identify molecular markers relevant for risk stratification. Through integrated histopathologic, genomic, epigenomic and clinical profiling, we show that pediatric and young-onset meningiomas represent a disease setting in which adult-derived diagnostic and prognostic frameworks have limited applicability. At the same time, our analyses confirm that classical clinical determinants, most notably extent of resection in tumors arising ≤21 years, remain central to patient outcome – underscoring the importance of interpreting molecular findings in conjunction with surgical management.

Adult meningioma risk stratification relies heavily on WHO grading, DNA methylation-based classification and specific high-risk alterations^5^, yet none of these metrics demonstrated prognostic value in patients up to 21 years of age. Hallmark adult driver events such as homozygous *CDKN2A/B* deletion or *TERT* promoter mutations^32–34^ were essentially absent, and DNA methylation-based prediction (without further stratification) failed to resolve clinically meaningful prognostic subgroups. These observations argue that the current adult classification architecture does not capture the dominant biological axes governing tumor behavior in younger patients.

Copy-number profiling further reinforces this divergence. Whereas adult tumors are characterized by recurrent losses (notably 1p and 22q)^23,32^, pediatric and young-onset meningiomas show a gain-dominated landscape, particularly involving chr17q. Canonical adult high-risk CNVs did not associate with outcome^5^, underscoring that prognostic features in early-onset disease arise from a distinct genomic context rather than from incomplete performance of adult frameworks. These findings parallel observations in other CNS tumor types in which pediatric disease forms biologically distinct molecular groups relative to adult counterparts^33,34^.

We could identify an age-dependent molecular gradient across the 0–39-year spectrum. Tumors arising up to approximately 21 years predominantly exhibit the pediatric-associated profile, marked by limited prognostic relevance of adult risk systems and a characteristic gain-centric CNV pattern, whereas tumors diagnosed thereafter progressively converge toward the adult molecular phenotype. Due to the low incidence of meningiomas up to 39 years of age, it is challenging to define in a truly data-driven manner at which point the underlying biological or clinical systems reliably apply and when they cease to do so. This issue is further enhanced given the slow growth and long latency typical of meningiomas. This also represents a limitation of our study, but at the same time, there is a lack of large multicenter datasets that would adequately address this question.

Here, we introduce a model that demonstrates improved performance compared to previously applied approaches from adults. However, despite these advances, a degree of uncertainty remains, particularly for patients in their mid-twenties, regarding accurate classification and clinical interpretation.

### Within this new framework, driver-defined subgroups emerge as biologically coherent entities

The mutational landscape of pediatric and young-onset meningiomas is generally distinct from adult tumors. *NF2* and *SMARCE1* mutations predominate, complemented by alterations in *BAP1* and *YAP1*. Although germline assessment was not systematic, the distribution and nature of these alterations suggest that hereditary predisposition may play a more prominent role than in adult disease. While the lack of systematically available information on germline status represents a limitation of this study, it in fact reflects current clinical reality. *SMARCE1*- and *BAP1*-altered meningiomas represent distinct molecular subsets^20,31^ that are typically associated with higher-grade histology (WHO grade 2/3) and appear biologically separate from *NF2*-driven and “other” tumors.

However, within the pediatric-associated subgroup (0–21 years), the extent of resection remained the strongest predictor of progression across molecular categories. This observation emphasizes that classical clinical factors, particularly extent of resection, remain the dominant determinants of outcome in this cohort and must be considered when interpreting molecular associations. The role of adjuvant radiotherapy in this retrospective cohort is more difficult to disentangle because treatment decisions are inherently influenced by tumor grade, residual disease and clinical judgment, introducing potential confounding by indication. Within *NF2*-mutated tumors, additional refinement by co-occurring genomic features such as chr17q gain may further improve biological resolution. In contrast, within the “other” subgroup, DNA methylation-based classification retains partial prognostic utility, suggesting that the relevance of epigenetic class is context-dependent. Together, these observations support a hierarchical interpretation of molecular features in which driver alterations define primary biological groups and additional genomic features refine risk within those contexts.

Although the integration of multiple molecular layers supports the robustness of these observations, outcome data and germline information were not uniformly available across the cohort, limiting statistical power in some subgroup analyses. Further, low case numbers for *BAP1*-altered and *YAP1*-fusion tumors restrict definitive prognostic conclusions. In addition, the retrospective design and variability in clinical management - including surgical approach and use of radiotherapy - introduce potential confounding that cannot be fully addressed without prospective validation, particularly given incomplete data on radiotherapy timing and intent and the limited event numbers available for multivariable modeling. Independent cohorts and prospective studies will therefore be important to confirm the prognostic relevance of the molecular patterns identified here.

In summary, pediatric and young-onset meningiomas occupy a molecular and clinical landscape that differs substantially from adult disease, characterized by distinct driver alterations, age-associated epigenetic signatures and a gain-centric copy-number architecture. While the extent of resection remains a principal determinant of outcome in tumors arising in younger patients, our findings suggest that molecular stratification can provide additional biological context that refines risk assessment in younger patients. These results provide a framework for age-adapted diagnostic and prognostic strategies tailored to pediatric and young-onset meningioma.

## Methods

### Patient samples and ethical approval

We retrospectively obtained clinical data and archived formalin-fixed, paraffin-embedded (FFPE) tumor samples from multiple collaborating centers, which were subsequently consolidated and analyzed at the Department of Neuropathology, University Hospital Heidelberg (Germany). Contributing centers were located in Germany (Heidelberg, Mannheim, Berlin, Hannover, Hamburg, Giessen, Magdeburg and Tübingen), the Netherlands (Nijmegen, Rotterdam, Amsterdam, Utrecht, and Groningen), France (Paris), Italy (Florence), Turkey (Ankara and Istanbul), Spain (Barcelona) and the United States (St. Jude Children’s Research Hospital, Memphis, TN; Washington University School of Medicine, St. Louis, MO; and University of California, San Francisco, CA). A small subset of data was generated within the framework of the Molecular Neuropathology 2.0 study^35^.

Sample selection was based on an age cut-off of ≤39 years, documentation of patient sex (based on self-report) and the availability of DNA sequencing and DNA methylation data with a meningioma score of ≥0.8 in the Heidelberg DNA methylation classifier (V12.8)^26,36^. Extent of resection (EOR) was defined using the surgeon-reported Simpson grading system, with gross total resection (GTR) classified as Simpson grades I–III and subtotal resection (STR) as Simpson grades IV–V. Radiotherapy was defined as adjuvant treatment administered before documented progression following index surgery, based on available clinical documentation. Owing to the retrospective multicenter design, systematic early postoperative MRI data for independent verification of EOR, as well as detailed information on radiotherapy timing (e.g., fixed postoperative intervals) and salvage treatment, were not consistently available. The study was conducted in accordance with the ethics approval of the University of Heidelberg (S-318/2022; S-224/2024). Due to the retrospective design of this study, informed consent was waived and all patient data were pseudonymized before analysis.

### DNA methylation array processing and copy-number profiling

Genome-wide DNA methylation profiling was performed using the Infinium MethylationEPIC (EPIC and EPICv2) or HumanMethylation450 (450k) BeadChip arrays (Illumina) following the manufacturer’s protocol, as previously described^36^. Duplicates were identified by pairwise correlation of genotyping probes to rule out samples that were used more than once. Computational analyses were performed in R (version 4.6.1). Copy-number variation analysis was conducted using the conumee Bioconductor package (version 1.12.0). Unsupervised non-linear dimension reduction was performed using probes that remained after standard filtering^36^. Variance was calculated for all remaining probes and the 10,000 most variable probes were selected. These probes were used to compute the 1–variance-weighted Pearson correlation between samples. t-SNE analysis was carried out using the Python openTSNE package (v1.0.2) with non-default parameters (max_iter = 1000, perplexity = 20, learning_rate = “auto”, and variance-weighted Pearson correlation as the distance metric).

### DNA methylation class prediction UCSF classifier

For the UCSF meningioma methylation-based classification^8^, the published classifier was applied to the EPICv1 cases. EPICv2 cases were classified using a modified version of the classifier restricted to the CpG sites represented on the EPICv2 array; when applied to EPICv1 data, this reduced classifier achieved a 94% concordance with the original classification. For the prediction of the 450k cases, the CpG sites were selected accordingly, and a new classifier was trained on this subset; this classifier achieved an accuracy of 95%.

### Deconvolution of bulk methylation data

We obtained publicly available DNA methylation data from the Gene Expression Omnibus generated using EPIC and EPICv2 or 450k BeadChip arrays (Illumina) applied to purified cells. Specifically, we downloaded methylation datasets pertaining to B cell, CD4 T cell, CD8 T cell, monocyte and natural killer from GSE184269 (ref. ^37^); CD4 T cell and CD4 T cell from GSE56581 (ref. ^38^); CD4 naive T cell, CD4 memory T cell, basophil, B memory cell, B naive cell, T reg, CD8 effector memory T cell, CD8 naive T cell, eosinophil, natural killer, neutrophil and monocyte from GSE167998 (ref. ^39^); endothelium from GSE140295 (ref. ^40^); endothelium from GSE82234 (ref. ^41^); endothelium from GSE84395 (ref. ^42^); fibroblast and endothelium from GSE74877 (ref. ^43^); glia from GSE166207 (ref. ^44^); hematopoietic stem cell, multipotent progenitor, lymphoid-primed multipotent progenitors, common myeloid progenitor, granulocyte/macrophage progenitor and megakaryocyte/erythroid progenitors from GSE63409 (ref. ^45^); microglia from GSE191200 (ref. ^46^); neuron and endothelium from GSE122126 (ref. ^47^); neuron and glia from GSE50798 (ref. ^48^); neuron from GSE112179 (ref. ^49^); neutrophil, CD4 T cell, CD8 T cell, natural killer, B cell and monocyte from GSE88824 (ref. ^50^); neutrophil, natural killer, B cell, CD4 T cell, CD8 T cell and monocyte from GSE110554 (ref. ^39^); neutrophil, natural killer, B cell, CD4 T cell, CD8 T cell and monocyte from GSE110555 (ref. ^51^). A methylation profile for a CNS tumor-specific cancer signal was derived from the 500 most pure tumors within the Heidelberg methylation classifier data, as determined by RF_purify^52^.

We used the Bioconductor package minfi to obtain raw signal intensities from IDAT files. We normalized each sample individually by performing a background correction (shifting of the 5th percentile of negative control probe intensities to 0) and a dye-bias correction (scaling of the mean of normalization control probe intensities to 10,000) for both color channels. We used MethylCibersort^53^ to construct a cell-type reference matrix with 1,574 CpG sites and employed non-negative least squares for cell-type deconvolution from bulk methylation array data.

### Comparison of deconvolution data

Deconvolution values are normalized to sum to 1 and transformed to avoid 0 or 1 entries using the DR_data function in the R package DirichletReg. Pivot logratio (PLR) transformation of components is done, which is a special case of isometric logratio (ILR) transformation^54^. A multivariate ANOVA based on logratios is used to test globally for a difference in cell compositions between groups^55^. Each individual component z is compared with a t-test between groups. For that, the first Pivot logratio coordinate (PLR1) is computed with the corresponding component z as pivot variable^56^. This logratio can be interpreted as the relative dominance of component z in the composition compared to the average of the other components. Note that t-test results are numerically equivalent to using centered logratio transformed (CLR) components. For multivariable analysis, a linear model with outcome PLR1 and predictors age group and driver mutations or MC was fitted. P-values across components are adjusted for multiple testing controlling the false discovery rate. Geometric mean barplots from the R package coda.plot are used to plot relative compositions for different groups on a CLR scale.

### Immunohistochemistry and evaluation

To assess CD68, progesterone receptor (PR) and estrogen receptor (ER) expression, automated immunohistochemistry (IHC) was performed using the Ventana Benchmark ULTRA system (Ventana Medical Systems Inc.) on 4 μm FFPE tissue sections. Staining was carried out using a CD68 antibody (Dako M0876, clone PG-M1), PR antibody (Biogenex Laboratories, clone PR88) and ER antibody (Thermo Scientific, clone SP1). Following deparaffinization and heat-induced antigen retrieval with CC1 solution (#950-500, Ventana), samples were incubated with the primary antibody for 36 min. Detection was carried out using the OV or UV detection system, followed by counterstaining with Hematoxylin II for 4 min and bluing reagent for 20 min. The slides were digitized using an Aperio AT2 scanner (Leica). Quantification of immunohistochemistry CD68 signals was performed using MIKAYA image analysis software.

For the evaluation of nuclear progesterone receptor (PR) expression, a semi-automated custom cell-detection workflow was employed. Briefly, color deconvolution followed by the Positive Cell Detection command with customized parameters was applied to PR-stained meningioma specimens in QuPath (version 0.5.0). Nuclear DAB intensity was quantified using three predefined intensity thresholds to derive the H-score. Finally, a cell classifier was trained and implemented to exclude detections corresponding to erythrocytes and other artifacts.

### Targeted DNA and RNA sequencing

Genomic DNA was extracted from FFPE tumor tissue using the Maxwell 16 FFPE Plus LEV DNA Purification Kit (Promega). Targeted next-generation DNA sequencing was performed at the Department of Neuropathology, University Hospital Heidelberg, using custom-designed, enrichment-based panels covering the coding regions (all exons ±25 bp) and selected intronic and promoter regions of CNS tumor–related genes^57^. Coverage of specific genes, including *BAP1*, was not uniform across all samples; however, tumors lacking detectable *BAP1* alterations by sequencing did not cluster within the *BAP1*-associated DNA methylation class, supporting their classification as *BAP1*-wildtype^31^. The panel was designed to detect single-nucleotide variants, small insertions and deletions, copy-number alterations, exonic rearrangements and recurrent fusion events. Sequencing was carried out on Illumina NextSeq 500, NovaSeq 6000, or NovaSeq X platforms using paired-end reads. Sequence data were aligned to the human reference genome GRCh37 (hg19) using the Burrows–Wheeler Aligner (BWA). Variants with an allele frequency below 5% were excluded, and pathogenic or likely pathogenic variants were classified according to American College of Medical Genetics and Genomics (ACMG) criteria. Systematic germline testing was not available for this retrospective cohort; therefore, germline status could not be comprehensively assessed across all cases.

In a subset of tumors, targeted RNA sequencing was performed as previously described^31,57^ to enable detection of gene fusions. Briefly, 200 ng of total RNA was processed using the Agilent SureSelect XT HS2 RNA workflow, followed by hybrid-capture enrichment with SureSelect Human All Exon V8 probes (Agilent). Dual-indexed libraries were amplified, quality-controlled using an Agilent 4150 TapeStation, pooled equimolarly and sequenced on an Illumina NovaSeq 6000 platform using 2 × 100-bp paired-end reads. Demultiplexed FASTQ files were generated using Illumina bclconvert (v3.10.5) and reads were aligned to the Gencode GRCh37 primary assembly using STAR (v2.7.7a). Gene fusion events were identified using Arriba (v2.4.0), focusing on high-confidence fusion calls.

### Statistics

No statistical method was used to predetermine sample size. No data were excluded from the analyses. The study was not randomized and investigators were not blinded during data collection and outcome assessment. Fisher’s exact test was used to compare categorical variables, Wilcoxon test for continuous variables. All tests were two-sided. Progression-free survival times were estimated by the Kaplan–Meier method and compared between risk groups with the log-rank test and univariable Cox proportional hazard regression models. Subgroup effects were displayed in forest plots. Oncogenetic trees were estimated using the R package Oncotree. For the newly established risk model (Fig. 5A) for patients 0-21 years of age, a multivariable Cox PH model with Firth correction was fitted to address the potential bias due to the low events per variable ratio. With 5 parameters to be estimated and 39 events, the event per variable (EPV) ratio of 7.8 was low but acceptable. Missing value imputation was done using MICE^58^, based on 50 imputation runs. Proportional hazard assumptions were verified based on the scaled Schoenfeld residual approach^59^. The c-index was used to assess prognostic discrimination of different risk stratifications. Propensity score-weighted Cox regression based on multiply imputed data sets was performed using R packages MatchThem, cobalt and Weightit. The propensity score weights were estimated with a logistic regression model using age, sex, EOR, WHO grade and diagnosis timepoint as predictors. P-values below 0.05 were considered statistically significant. Analyses were performed with R 4.5.1.

### Reporting summary

Further information on research design is available in the Nature Portfolio Reporting Summary linked to this article.

## Supplementary information

Supplementary Information

Reporting Summary

Transparent Peer Review file

## Source data

Source Data

## Data Availability

DNA methylation data generated in this study have been deposited in the NCBI Gene Expression Omnibus (GEO) under accession code GSE317578. Public DNA methylation datasets used for deconvolution analyses were obtained from GEO under accession codes GSE50798 (ref. ^48^), GSE56581 (ref. ^38^), GSE63409 (ref. ^45^), GSE74877 (ref. ^43^), GSE82234 (ref. ^41^), GSE84395 (ref. ^42^), GSE88824 (ref. ^50^), GSE110554 (ref. ^39^), GSE110555 (ref. ^51^), GSE112179 (ref. ^49^), GSE122126 (ref. ^47^), GSE140295 (ref. ^40^), GSE166207 (ref. ^44^), GSE167998 (ref. ^39^), GSE184269 (ref. ^37^) and GSE191200 (ref. ^46^). Raw targeted DNA and RNA sequencing data contain potentially identifiable human genetic information and are therefore subject to the conditions of the ethics approvals granted by the Ethics Committee of the Medical Faculty Heidelberg, Heidelberg University (S-318/2022 and S-224/2024), as well as the requirements of the European General Data Protection Regulation (GDPR). The informed consent framework for this retrospective study does not permit unrestricted public sharing of individual-level genomic data. Consequently, raw sequencing data are not publicly available. Filtered sequencing data will be made available for non-commercial research purposes upon approval of a data access request and completion of a data transfer agreement with the corresponding author. These data will remain available for at least 10 years following publication. Requests will be acknowledged within 14 days. Source data are provided with this paper. All other data supporting the findings of this study are available within the Article and Supplementary Information. Source data are provided with this paper.
