# Supplementary material for: Distinct molecular subgroups in pediatric and young-onset meningiomas require age-adapted risk stratification
Source: Nat Commun. 2026 Jul 14;17:6188. doi: 10.1038/s41467-026-75357-2 (PMC13369989; doi:10.1038/s41467-026-75357-2)
Supplement: Supplementary file 1 — Supplementary Information [file 41467_2026_75357_MOESM1_ESM.pdf]

Supplementary Information

Distinct molecular subgroups in pediatric and young-onset meningiomas require age-adapted risk stratification

Natalie Berghaus*, Arnault Tauziède-Espariat*, Thomas Hielscher*, Dilan Savran, Daniel Schrimpf, Kirsten Göbel, Eric Stutheit-Zhao, Lukas Friedrich, Felix Keller, Fuat Kaan Aras, Filippo Nozzoli, Dominik Sturm, Christine L. White, Simone Schmid, Christian Mawrin, Julia E. Neumann, Till Acker, Rudi Beschorner, Christian Hartmann, Irem Saribiyik, Arda Inan, Ayça Erşen-Danyeli, Mariëtte E. G. Kranendonk, Sybren L. N. Maas, Eelke M. Bos, Eleonora Aronica, Saskia M. Peerdeman, Nikki B. Thuijs, Angelika Mühlebner, Benno Kusters, Wilfred F. A. den Dunnen, Cinzia E. Lavarino, Stéphanie Puget, Jason Chiang, Sonika Dahiya, Melike Pekmezci, Arie Perry, Oluwadamilola Akanji, Miriam Ratliff, Christel Herold-Mende, Sandro M. Krieg, Wolfgang Wick, Stefan M. Pfister, Pieter Wesseling, Andreas von Deimling, Pascale Varlet#, Felix Sahm#, Philipp Sievers#

* These authors contributed equally
# These authors jointly supervised this work

Corresponding author
Philipp Sievers, MD
Department of Neuropathology
Institute of Pathology
University Hospital Heidelberg
and
Clinical Cooperation Unit Neuropathology,
German Consortium for Translational Cancer Research (DKTK)
German Cancer Research Center (DKFZ)
Im Neuenheimer Feld
Heidelberg, Germany
Fon: +49-6221 40 1474; Fax: +49-6221 56 4566
philipp.sievers@med.uni-heidelberg.de

SI Figures

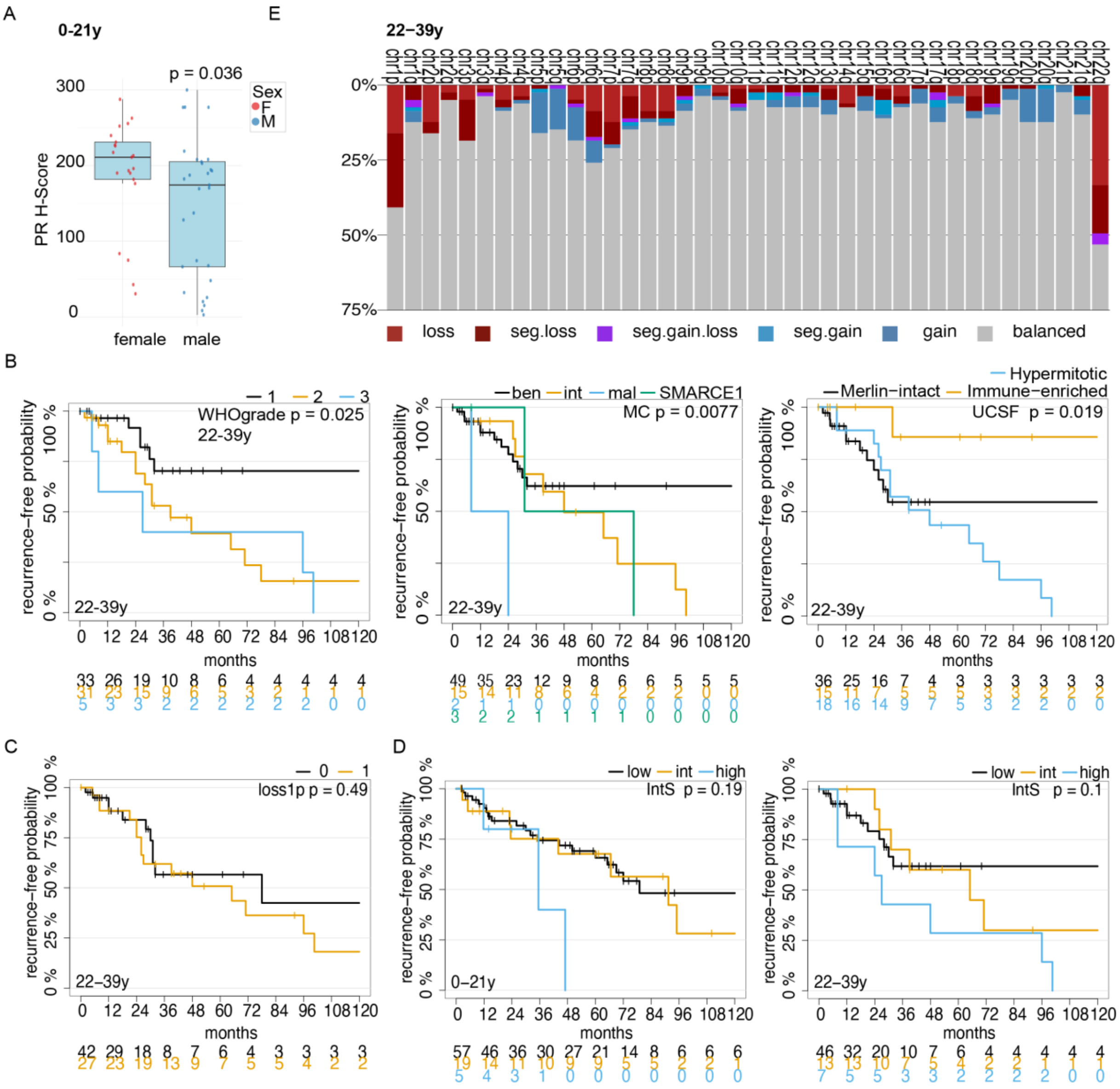

Supplementary figure 1. Progesterone staining results, copy number plot and additional survival analysis. (A) Boxplot comparing progesterone receptor staining H-scores between meningiomas from female (n = 22) and male (n = 29) patients 0-21 years of age. The p-values were calculated using the Wilcoxon rank sum test. Horizontal lines of the box indicate Q1, median (bold) and Q3 of the distribution, whiskers extend to the most extreme data point which is no more than 1.5*IQR from the box away. PR = progesterone receptor, F = female, M = male, y = years. (B) Kaplan-Meier analysis of risk of progression of the subgroup of patients 22-39 years of age stratified according to WHO grade (WHO grade 1 n = 33, WHO grade 2 n = 31, WHO grade 3 n = 5), methylation class[7] (ben n = 49, int n = 15, mal n = 2, SMARCE1 n = 3) and UCSF class[8] (Hypermitotic n = 18, Merlin-intact n = 36, Immune-enriched n = 15). The p-values were calculated using the Log-rank test. MC = methylation class, Ben = benign, int = intermediate, mal = malignant, y = years (C) Kaplan-Meier analysis of risk of progression of the subgroup of patients 22-39 years of age, stratified according to the presence (n = 27) or absence (n = 42) of a loss of chromosome 1p. The p-values were calculated using the Log-rank test. y = years (D) Kaplan-Meier analysis of risk of progression of the subgroup of patients 0-21 and 22-39 years of age, stratified according to integrated risk score[23] (0-21 years: low n = 57, int n = 19, high n = 5; 22-39 years low n = 46, int n = 13, high n = 7). The p-values were calculated using the Log-rank test. (E) Overview of chromosomal alterations in meningiomas from 22-39-year-old patients. seg. = segmental, chr = chromosome, y = years. Source data are provided as a Source Data file.

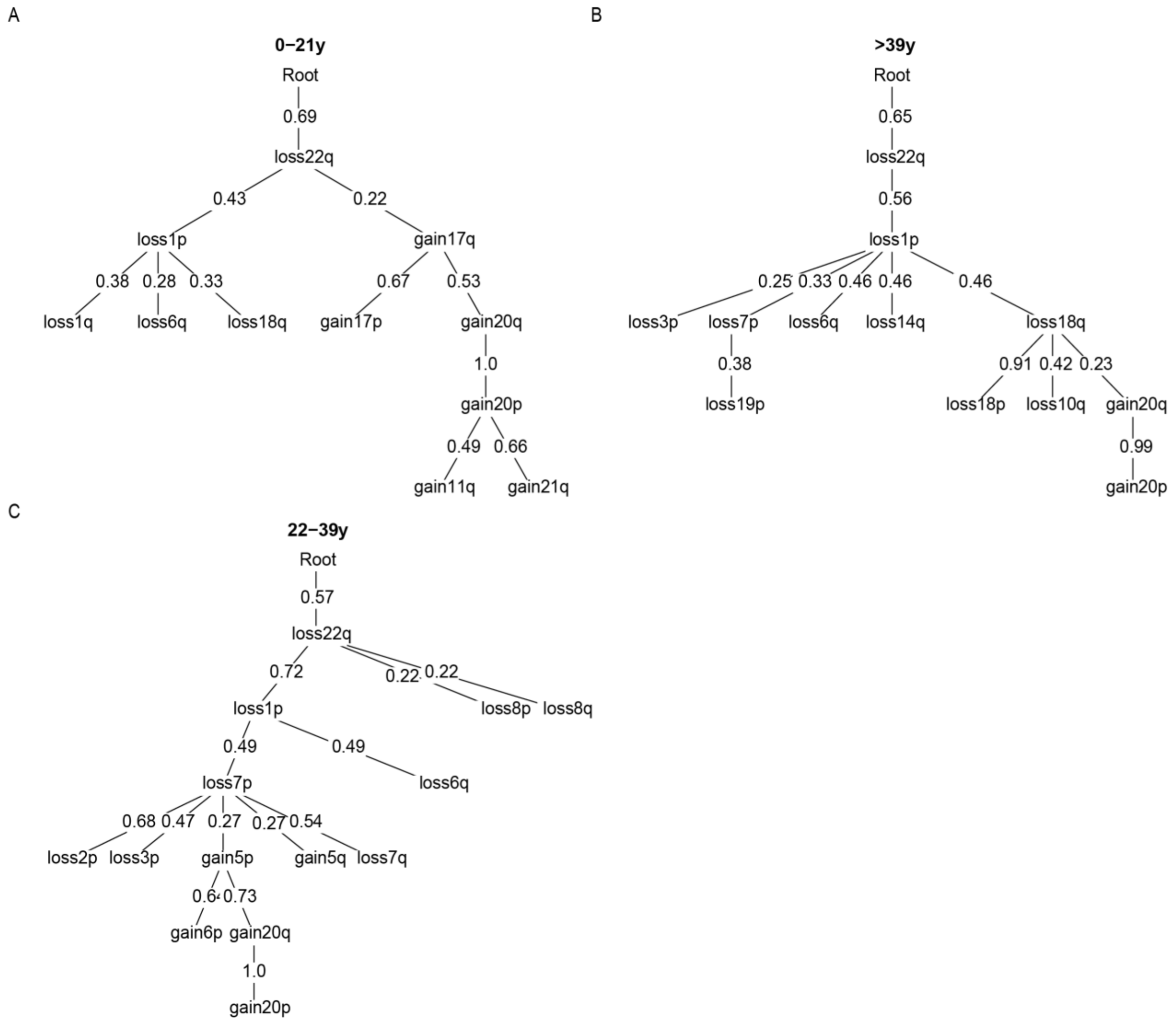

Supplementary figure 2. Oncogenetic trees for meningiomas in the age groups (A) 0-21 years (n = 212), (B) 22-39 years (n = 81) and (D) >39 years (n = 747). Only gains/losses with at least 10% prevalence within the respective age cohort were used. y = years. Source data are provided as a Source Data file.

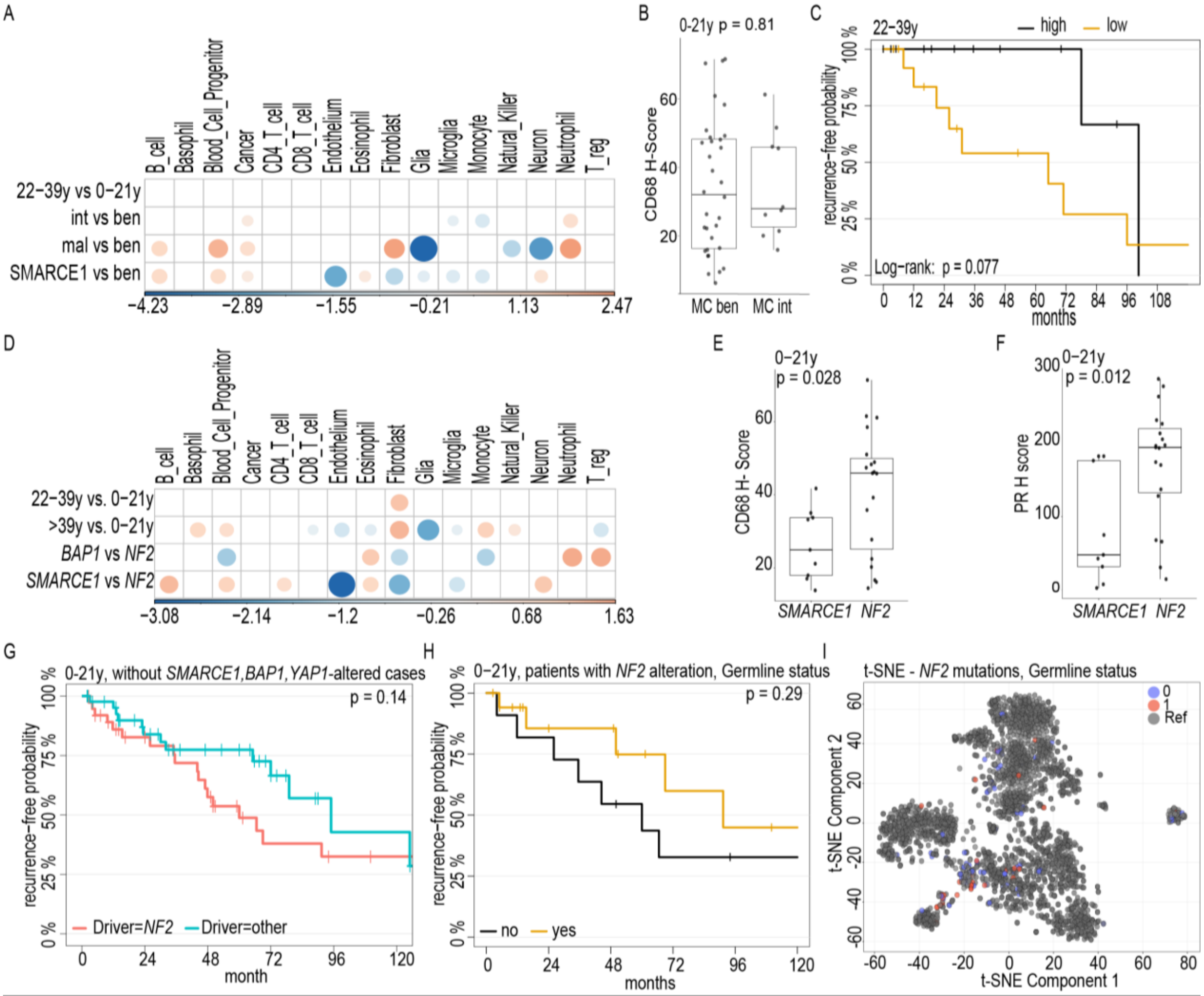

Supplementary figure 3. Additional analyses of the impact of the tumor microenvironment and mutational status. (A) multivariable analysis for tumor microenvironment for age cohort and methylation classes, patients 0-39 years: For each individual tumor microenvironment component, a multivariable linear model with the tumor microenvironment component (using PLR1 transformation) as outcome is fitted. I.e., each column in the plot gives estimates from one multivariable model. Only significant effects are displayed. P-values are adjusted across tumor microenvironment components. Red/blue indicates a relative increase/decrease of a tumor microenvironment component associated with a certain factor. MC = methylation class. (B) Boxplots comparing CD68 H-scores between meningiomas from patients 0-21 years with meningiomas classified as intermediate (n = 10) and benign (n =32). The p-values were calculated using the Wilcoxon rank sum test. Horizontal lines of the box indicate Q1, median (bold) and Q3 of the distribution, whiskers extend to the most extreme data point which is no more than 1.5*IQR from the box away. MC = methylation class, ben = benign, int = intermediate (C) Kaplan-Meier analysis of risk of progression of the subgroup of patients 22–39 years stratified for the top (n = 13) and bottom 20% (n = 13) of microglia share based on the bulk methylation deconvolution data. The p-values were calculated using the Log-rank test. (D) multivariable analysis for tumor microenvironment for age cohort and driver group, all patients excluding driver group other (0-39 years, n = 267): For each individual tumor microenvironment component, a multivariable linear model with the tumor microenvironment component (using PLR1 transformation) as outcome is fitted. I.e., each column in the plot gives estimates from one multivariable model. Only significant effects are displayed. P-values are adjusted across tumor microenvironment components. Red/blue indicates a relative increase/decrease of a tumor microenvironment component associated with a certain factor. MC = methylation class. (E) Boxplot comparing CD68 H-scores between *SMARCE1* (n = 9) and *NF2*-altered (n = 19) meningiomas. The p-values were calculated using the Wilcoxon rank sum test. Horizontal lines of the box indicate Q1, median (bold) and Q3 of the distribution, whiskers extend to the most extreme data point which is no more than 1.5*IQR from the box away. (F) Boxplot comparing progesterone receptor H-scores between *SMARCE1* (n = 10) and *NF2*-altered (n = 20) meningiomas. The p-values were calculated using the Wilcoxon rank sum test. PR = progesterone receptor. Horizontal lines of the box indicate Q1, median (bold) and Q3 of the distribution, whiskers extend to the most extreme data point which is no more than 1.5*IQR from the box away. (G) Kaplan-

Meier analysis of risk of progression of the subgroup of patients 0-21 years of age, presenting with meningiomas without a *BAP1* and *SMARCE1* alteration, stratified for the presence of an *NF2* alteration (*NF2* n = 38, other n = 44). The p-value was calculated using the Log-rank test. (H) Kaplan-Meier analysis of risk of progression of the subgroup of patients 0-21 years of age, presenting with *NF2*-altered meningiomas stratified for the presence of a germline *NF2*-mutation (yes n = 18, no n = 10). The p-value was calculated using the Log-rank test. (**I**) Methylation Clustering displayed as a t-SNE projection (20 perplexity, 1000 iterations, distance metric: weighted Pearson) of the pediatric and young-onset tumors of this study with 2,197 meningiomas retrieved from the database of the neuropathology department in Heidelberg with available information on age and sex of the patients and a methylation family score for meningioma >0.9. Cases were colored based on information on *NF2* mutation and respective germline status. Source data are provided as a Source Data file.

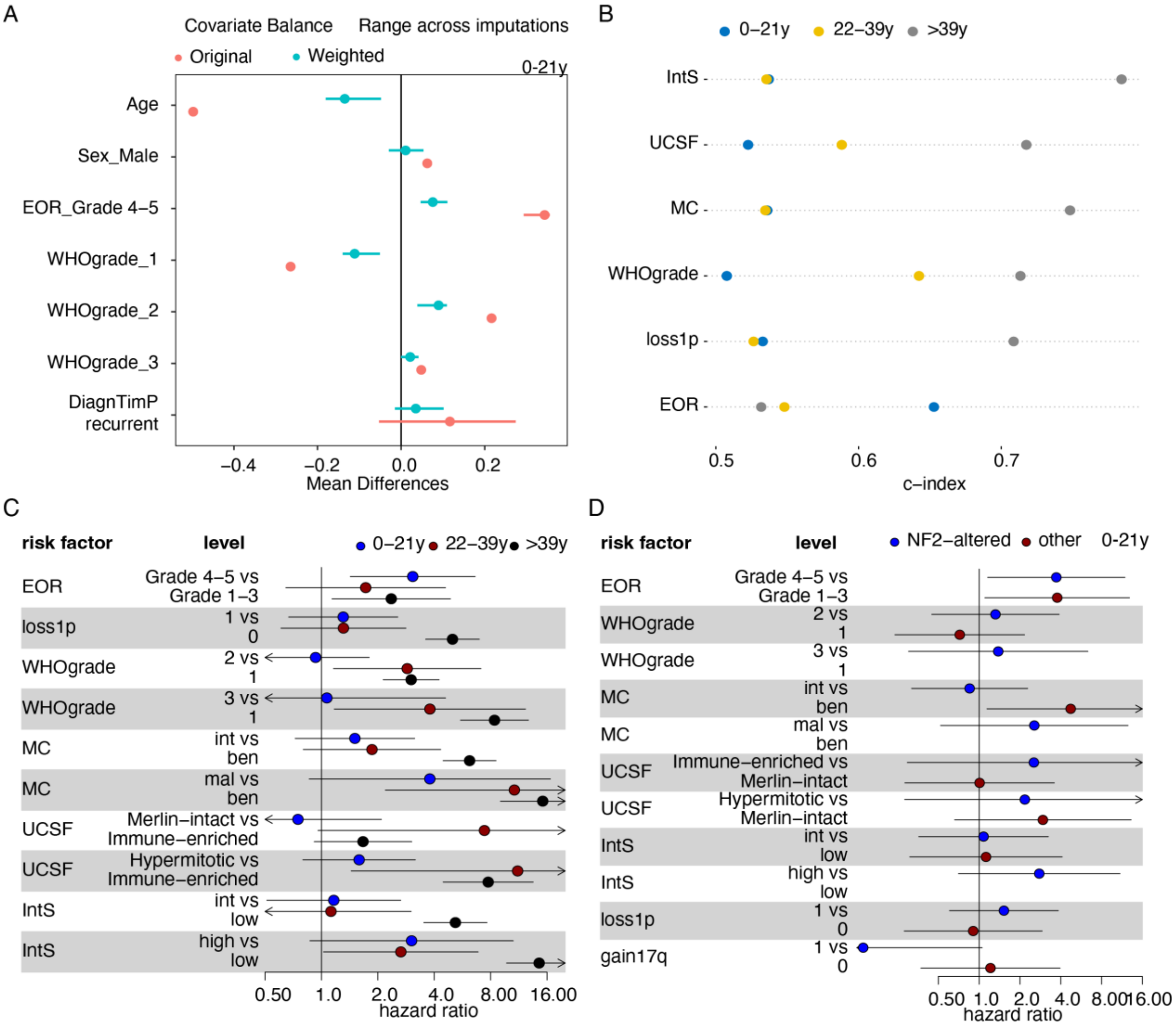

Supplementary figure 4. Additional analyses investigating the influence of different factors on patient risk (A) ‘love’ plot showing the covariate balance between patients receiving/not receiving radiotherapy before and after propensity score (PS) weighting across imputed data sets in patients 0-21years. EOR = extend of resection based on Simpson grading. DiagnTimP = Timepoint of Diagnosis. Sex male n = 22, EOR grade 4-5 n = 15, WHO grade 1 n = 20, WHO grade 2 n = 17, WHO grade 3 n = 4, Diagnose timepoint recurrent n = 2 (B) Comparison of the c-index among the patient groups 0-21-years-ols and 22-39-years-old and patients > 39 years of age across different stratification systems. IntS = Integrated meningioma risk score3 0-21 years low n = 57 int n = 19 high n = 5, 22-39 years low n = 46 int n = 13 high n = 7, >39 years low n = 396, int n = 224, high n = 112; UCSF = UCSF methylation classes2 0-21 years: Merlin-intact n = 17 Immune-enriched n = 39 Hypermitotic n = 40, 22-39 years: Merlin-intact n = 36 Immune-enriched n = 15 Hypermitotic n = 18, >39 years Merlin-intact n = 263 Immune-enriched n = 153 Hypermitotic n = 201; MC = Heidelberg methylation classes1 0-21 years: ben n = 60, int n = 20 mal n = 3 SMARCE n = 13, 22-39 years ben n = 49 int n = 15, mal n = 2 SMARCE n = 3, WHO = WHO grades 0-21 years: WHO grade 1 n = 47 WHO grade 2 n = 42 WHO grade 3 n = 5, 22-39 years: WHO grade 1 n = 33 WHO grade 2 n = 31 WHO grade 3 n = 5, >39 years: WHO grade 1 n = 397 WHO grade 2 n = 280 WHO grade 3 n = 63; loss1p = loss of chromosome 1p 1p 0-21 years: 1p balanced n = 69 1p loss n = 27, 22-39 years 1p balanced n = 42 1p loss n = 27, >39 years 1p balanced n = 470 1p loss n = 277; EOR = Extend of resection based on Simpson Grading 0-21 years grade1-3 n = 50 grade 4-5 n = 24, 22-39 years: grade 1-3 n = 47 grade 4-5 n = 11, >39 years: grade 1-3 n = 335 grade 4-5 n = 23. (C) Summary of risk factors and their corresponding hazard ratios between patients 0-21, 22-39 and >39 years of age. EOR = extend of resection based on Simpson Grading (0-21 years grade 4-5 n = 24, grade 1-3 n = 50; 21-39 years grade 4-5 n = 11, grade 1-3 n = 43; >39 years grade 4-5 n = 23, grade1-3 n = 335), IntS = Integrated meningioma risk score by Maas et al. (2021) (0-21 years high n = 4, int n = 20, low n = 59; 21-39 years high n = 7, int n = 13, low n = 46; >39 years low n = 392, int n = 222, high n = 112), WHO: 0-21 years grade 3 n = 5, grade 2 n = 42, grade 1 n = 47; 21-39 years grade 1 n = 33, grade 2 n = 31, grade 3 n = 5; >39 years

grade 1 n = 394, WHO grade 2 n = 275, grade 3 n = 61; loss1p: 0-21 years balanced n = 69, loss/seg.loss n = 27; 21-39 years balanced n = 42, loss/seg.loss n = 27; >39 years balanced n = 440, loss/seg.loss n = 290) y = years; MC = methylation class, ben = benign, int = intermediate, mal = malignant (0-21 years ben n = 60, int n = 20, mal n = 3; 22-39 years ben n = 49, int n = 15, mal n = 2; >39 years ben n = 504, int n = 196, mal n = 28), UCSF 0-21 years Hypermitotic n = 18, Immune-enriched n = 15, Merlin-intact n = 17; 22-39 years Hypermitotic n = 40, Immune-enriched n = 39, Merlin-intact n = 36; >39 years Hypermitotic n = 40, Immune-enriched n = 39, Merlin-intact n = 17. Horizontal lines indicate the 95% confidence interval of the hazard ratio. GTR = gross total resection, STR = subtotal resection, CNA = copy number alterations, IntS = Integrated meningioma risk score. (D) Summary of risk factors and their corresponding hazard ratios between meningiomas from patients 0-21 years of age with NF2 alterations and the “other” subgroup, including or excluding cases presenting solely a loss of chromosome 22q. Horizontal lines indicate the 95% confidence interval of the hazard ratio. NF2-altered group: WHO grade 2 n = 12, WHO grade 1 n = 21, WHO grade 3 n = 7; EOR grade 4-5 n = 11, grade 1-3 n = 18; MC int n = 18, ben n = 20, mal n = 3; IntS int n = 8, low n = 24, high n = 4; loss1p balanced n = 23, loss/seg.loss n = 15; chr17q balanced n = 30, gain/seg.gain n = 8; UCSF: hypermitotic n = 49, immune-enriched n = 23, merlin-intact n = 7. “other” group: WHO grade 2 n = 17, WHO grade 1 n = 26, WHO grade 3 n = 1; EOR grade 4-5 n = 12, grade 1-3 n = 25; MC int n = 5, ben n = 40; IntS int n = 10, low n = 35, high n = 2; loss1p balanced n = 33, loss/seg.loss n = 12; chr17q balanced n = 36, gain/seg.gain n = 9; UCSF merlin-intact n = 33, immune-enriched n = 6, hypermitotic n = 8. EOR = extend of resection, MC = methylation class, int = intermediate, mal = malignant, CNA = copy number alterations, IntS = Integrated meningioma risk score. Source data are provided as a Source Data file.

SI Tables

Supplementary table 1. Overview and comparison of cohort characteristics across different age groups. MC = Heidelberg methylation class and subclass (MSC) proposed by Sahm et al. (2017)[7] and Sill et al. (2026)[26]. Ben = benign, Int = intermediate, Mal = malignant, UCSF = meningioma classification system proposed by Choudhoury et al. (2022)[8], IntS = Integrated risk score for meningiomas, proposed by Maas et al. (2021)[23]. Chr = chromosome, F = frameshift, M = missense, L = Splice, N = nonsense, y = year.

	0-21y, n = 212	22-39y, n = 81	>39y, n = 747
Female	102 (48.1%)	54 (66.7%)	509 (68.1%)
Male	110 (51.9%)	27 (33.3%)	238 (31.9%)
Simpson Grade 1-3	69 (69.0%)	46 (80.7%)	335 (93.6%)
Simpson Grade 4-5	31 (31.0%)	11 (19.3%)	23 (6.4%)
Simpson Grade Missing	112	24	389
WHO grade 1	103 (50.5%)	40 (49.4%)	397 (53.6%)
WHO grade 2	92 (45.1%)	35 (43.2%)	280 (37.8%)
WHO grade 3	9 (4.4%)	6 (7.4%)	63 (8.5%)
MC ben	126 (59.4%)	58 (71.6%)	511 (68.4%)
MC int	50 (23.6%)	16 (19.8%)	200 (26.8%)
MC mal	5 (2.4%)	3 (3.7%)	28 (3.7%)
MC SMARCE1	31 (14.6%)	4 (4.9%)	8 (1.1%)
MCS ben-1	51 (24.1%)	16 (19.8%)	197 (26.4%)
MCS ben-2	23 (10.8%)	20 (24.7%)	202 (27.0%)
MCS ben-3	52 (24.5%)	22 (27.2%)	111 (14.9%)
MCS int-A	49 (23.1%)	13 (16.0%)	160 (21.4%)
MCS int-B	1 (0.5%)	3 (3.7%)	41 (5.5%)
MCS mal	5 (2.4%)	3 (3.7%)	28 (3.7%)
MCS SMARCE1	31 (14.6%)	4 (4.9%)	8 (1.1%)
UCSF Merlin-intact	38 (17.9%)	42 (51.9%)	315 (42.5%)
UCSF Immune-enriched	81 (38.2%)	17 (21.0%)	181 (24.4%)
UCSF Hypermitotic	93 (43.9%)	22 (27.2%)	245 (33.1%)
IntS low	27 (38.6%)	53 (68.8%)	87 (26.0%)
IntS int	36 (51.4%)	16 (20.8%)	155 (46.3%)
IntS high	(10.0%)	8 (10.4%)	93 (27.8%)
loss chr1p - no	151 (71.2%)	48 (59.3%)	462 (61.8%)
loss chr1p - yes	61 (28.8%)	33 (40.7%)	285 (38.2%)
gain chr17q - no	173 (81.6%)	73 (90.1%)	674 (90.2%)
gain chr17q - yes	39 (18.4%)	8 (9.9%)	73 (9.8%)
clearcell Histology - no	173 (84.8%)	77 (95.1%)	555 (98.6%)
clearcell Histology - yes	31 (15.2%)	4 (4.9%)	8 (1.4%)
Location - orbital	8 (4.7%)	3 (3.9%)	-
Location - posterior	23 (13.4%)	8 (10.5%)	-
Location - skull base	30 (17.4%)	20 (26.3%)	-
Location - spinal	23 (13.4%)	2 (2.6%)	-

Location - supratentorial	73 (42.4%)	39 (51.3%)	-
driver.group - *BAP1*-altered	1 (0.5%)	5 (6.2%)	-
driver.group - *SMARCE1*-altered	29 (13.7%)	3 (3.7%)	-
driver.group - *NF2*-altered	79 (37.3%)	19 (23.5%)	-
driver.group - other	103 (48.6%)	54 (66.7%)	-
BAP1 alteration - no	176 (99.4%)	57 (91.9%)	-
BAP1 alteration - yes	1 (0.6%)	5 (8.1%)	-
SMARCE1 alteration - no	183 (86.3%)	78 (96.3%)	-
SMARCE1 alteration - yes	29 (13.7%)	3 (3.7%)	-
NF2 mutation - no	139 (65.6%)	62 (76.5%)	-
NF2 mutation - yes	73 (34.4%)	19 (23.5%)	-
NF2 mutation type - F	21 (28.8%)	5 (26.3%)	-
NF2 mutation type - L	11 (15.1%)	3 (15.8%)	-
NF2 mutation type - M	5 (6.8%)	1 (5.3%)	-
NF2 mutation type - N	36 (49.3%)	10 (52.6%)	-
NF2 and chr22q alteration - no	70 (33.0%)	38 (46.9%)	-
NF2 and chr22q alteration - yes	142 (67.0%)	43 (53.1%)	-

Supplementary table 2. Evaluation of deconvolution data with tests on individual log ratios between age groups. The 'adj p' gives the p-value adjusted for multiple testing controlling the false discovery rate. Groups are compared with a t-test using the first Pivot log-ratio coordinate of components. Adj.p. = adjusted p value, est = estimate, T reg = T regulator cell. 0-21 years n = 212, 22-39 years n = 81, >39 years n = 747. Source data are provided as a Source Data file.

	0-21 vs >39y	0-21 vs >39y	0-21 vs 22-39y	0-21 vs 22-39y	22-39 vs >39y	22-39 vs >39y
	est.	adj.p.	est.	adj.p.	est.	adj.p.
B cell	0.55	<0.001	0.26	0.541	0.28	0.120
Basophil	-1.00	<0.001	-0.36	0.541	-0.64	0.028
Blood Cell Progenitor	0.50	0.005	0.01	0.985	0.49	0.120
Cancer	0.60	<0.001	0.23	0.541	0.37	0.028
CD4 T-cell	-0.01	0.924	0.21	0.591	-0.22	0.360
CD8 T-cell	0.65	<0.001	0.19	0.541	0.46	0.020
Endothelium	0.46	0.004	-0.10	0.801	0.56	0.023
Eosinophil	-0.35	0.020	-0.10	0.801	-0.25	0.360
Fibroblast	-1.14	<0.001	-0.31	0.541	-0.83	0.020
Glia	0.87	<0.001	0.64	0.485	0.23	0.395
Microglia	0.45	<0.001	0.15	0.591	0.30	0.067
Monocyte	-0.24	0.244	-0.48	0.541	0.24	0.374
Natural Killer	-0.63	<0.001	-0.04	0.949	-0.59	0.033
Neuron	-1.01	<0.001	-0.38	0.541	-0.63	0.028
Neutrophil	-0.71	<0.001	-0.24	0.591	-0.47	0.120
T reg	1.03	<0.001	0.33	0.541	0.70	0.030

Supplementary table 3. Evaluation of deconvolution data with tests on individual log ratios between age groups and within methylation classes. The ‘adj p’ gives the p-value adjusted for multiple testing controlling the false discovery rate. Groups are compared with a t-test using the first Pivot log-ratio coordinate of components. Adj.p. = adjusted p value, est = estimate, T reg = T regulator cell. 0-21 years ben n = 58, int n = 16, mal n = 3; 22-39 years ben n = 58, int n = 16, mal n = 3. Source data are provided as a Source Data file.

MC	ben	ben	int	int	mal	mal
	0-21y vs 22-39y	0-21y vs 22-39y	0-21y vs 22-39y	0-21y vs 22-39y	0-21y vs 22-39y	0-21y vs 22-39y
	est.	adj.p.	est.	adj.p.	est.	adj.p.
B cell	0.19	0.696	0.30	0.569	-2.63	0.577
Basophil	-0.25	0.696	-0.96	0.569	1.39	0.603
Blood Cell Progenitor	0.24	0.696	-0.69	0.571	-4.00	0.465
Cancer	0.10	0.736	-0.09	0.937	0.58	0.577
CD4 T-cell	0.17	0.736	0.65	0.569	0.53	0.577
CD8 T-cell	0.35	0.696	0.06	0.937	-0.69	0.682
Endothelium	-0.07	0.844	0.17	0.932	0.55	0.577
Eosinophil	-0.28	0.696	-0.01	0.992	1.05	0.465
Fibroblast	0.07	0.861	-0.59	0.569	0.56	0.577
Glia	0.32	0.696	1.55	0.569	3.08	0.465
Microglia	0.31	0.696	0.10	0.937	-0.38	0.682
Monocyte	-0.10	0.844	-1.13	0.569	-1.56	0.577
Natural Killer	-0.23	0.696	0.77	0.569	0.53	0.577
Neuron	-0.70	0.506	-0.57	0.569	2.87	0.465
Neutrophil	-0.46	0.696	0.10	0.937	-0.48	0.682
T reg	0.35	0.696	0.37	0.932	-1.42	0.577

Supplementary table 4. Evaluation of deconvolution data with tests on individual log ratios between driver groups in the age group of 0-21-year-old patients. The ‘adj p’ gives the p-value adjusted for multiple testing controlling the false discovery rate. Groups are compared with a t-test using the first Pivot log-ratio coordinate of components. Adj.p. = adjusted p value, Est = estimate, T reg = T regulator cell. *NF2*-n = 79, *YAP1* n = 5, *SMARCE1* n = 29. Source data are provided as a Source Data file.

	NF2 vs *YAP1*	*NF2* vs *YAP1*	*SMARCE1* vs *YAP1*	*SMARCE1* vs *YAP1*	*SMARCE1* vs *NF2*	*SMARCE1* vs *NF2*
	est.	adj.p.	est.	adj.p.	est.	adj.p.
B cell	0.86	0.021	2.12	<0.001	1.26	0.016
Basophil	1.72	0.339	1.74	0.412	0.02	0.972
Blood Cell Progenitor	1.48	<0.001	2.73	<0.001	1.24	0.099
Cancer	1.40	<0.001	1.78	<0.001	0.38	0.380
CD4 T-cell	-0.30	0.976	0.20	0.907	0.49	0.380
CD8 T-cell	0.28	0.976	-0.10	0.907	-0.38	0.434
Endothelium	0.81	0.039	-1.72	0.027	-2.53	0.006
Eosinophil	0.11	0.980	0.74	0.690	0.63	0.255
Fibroblast	-1.28	0.009	-3.02	<0.001	-1.74	0.016
Glia	-0.06	0.976	-0.56	0.412	-0.50	0.434
Microglia	-0.74	0.052	-1.50	0.004	-0.76	0.191
Monocyte	-2.74	0.002	-3.06	<0.001	-0.32	0.668
Natural Killer	-1.71	0.328	-1.32	0.427	0.39	0.477
Neuron	-1.43	0.018	-0.32	0.702	1.12	0.099
Neutrophil	1.57	0.339	1.93	0.294	0.36	0.576
T reg	0.03	0.983	0.36	0.907	0.33	0.667

Supplementary table 5. Evaluation of deconvolution data with tests on individual log ratios between driver groups in patients >39 years of age. The ‘adj p’ gives the p-value adjusted for multiple testing controlling the false discovery rate. Groups are compared with a t-test using the first Pivot log-ratio coordinate of components. Adj.p. = adjusted p value, Est = estimate, T reg = T regulator cell. *NF2* n = 107, *SMARCE1* n = 8. Source data are provided as a Source Data file.

	SMARCE1 vs *NF2*	*SMARCE1* vs *NF2*
	est.	adj.p.
B cell	2.02	0.072
Basophil	-0.20	0.917
Blood Cell Progenitor	0.71	0.670
Cancer	0.25	0.668
CD4 T-cell	1.12	0.417
CD8 T-cell	-0.34	0.525
Endothelium	-4.39	0.050
Eosinophil	1.91	0.106
Fibroblast	-2.11	0.081
Glia	-1.20	0.304
Microglia	-1.70	0.106
Monocyte	-0.76	0.365
Natural Killer	1.60	0.106
Neuron	1.84	0.034
Neutrophil	1.25	0.106
T reg	-0.01	0.991

Supplementary table 7. Information on available radiotherapy data in the age groups: 0-21 years and 22-39 years. EOR = extend of resection according to Simpson grading. Adj. = adjuvant, RT = radiotherapy.

	0-21 years	0-21 years	22-39 years	22-39 years
	No (n=26)	Adj. RT (n=16)	No (n=30)	Adj. RT (n=11)
EOR				
Grade1-3	19 (73.1%)	6 (40.0%)	28 (93.3%)	4 (36.4%)
Grade4-5	7 (26.9%)	9 (60.0%)	2 (6.7%)	7 (63.6%)
Missing	0	1	0	0
WHO grade				
1	15 (57.7%)	5 (31.3%)	21 (70.0%)	3 (27.3%)
2	9 (34.6%)	9 (56.3%)	9 (30.0%)	7 (63.6%)
3	2 (7.7%)	2 (12.5%)	0	1 (9.1%)

Supplementary table 8. multivariable Cox PH regression model for the age group 0-21 years. HR = hazard ratio, LCL = Lower Confidence Limit, UCL = Upper Confidence Limit, EOR = Extent of resection based on Simpson Grading, int = intermediate. Source data are provided as a Source Data file. Sex: male n = 48, WHO grade 1 n = 47, WHO grade 2 n = 42, WHO grade 3 n = 5; EOR grade 4-5 n = 24, Risk group int n = 48

	HR	LCL95	UCL95	p-value
Sex Male	1.72	0.85	3.48	0.115
WHO grade 2	0.86	0.44	1.70	0.642
WHO grade 3	1.15	0.30	4.42	0.827
EOR grade 4-5	2.86	1.33	6.15	0.001
Risk group int	2.51	1.23	5.15	0.023

Supplementary References

1 Price, M. *et al.* CBTRUS Statistical Report: Primary Brain and Other Central Nervous System Tumors Diagnosed in the United States in 2018-2022. *Neuro Oncol* **27**, iv1-iv66 (2025). https://doi.org/10.1093/neuonc/noaf194

2 Tauziede-Espariat, A., Pfister, S. M., Mawrin, C. & Sahm, F. Pediatric meningiomas: A literature review and diagnostic update. *Neurooncol Adv* **5**, i105-i111 (2023). https://doi.org/10.1093/noajnl/vdac165

3 Kirches, E. *et al.* Molecular profiling of pediatric meningiomas shows tumor characteristics distinct from adult meningiomas. *Acta Neuropathol* **142**, 873-886 (2021). https://doi.org/10.1007/s00401-021-02351-x

4 Wang, J. Z. *et al.* Meningioma: International Consortium on Meningiomas consensus review on scientific advances and treatment paradigms for clinicians, researchers, and patients. *Neuro Oncol* **26**, 1742-1780 (2024). https://doi.org/10.1093/neuonc/noae082

5 Sahm, F. *et al.* cIMPACT-NOW update 8: Clarifications on molecular risk parameters and recommendations for WHO grading of meningiomas. *Neuro Oncol* **27**, 319-330 (2025). https://doi.org/10.1093/neuonc/noae170

6 Suppiah, S. *et al.* Molecular and translational advances in meningiomas. *Neuro Oncol* **21**, i4-i17 (2019). https://doi.org/10.1093/neuonc/noy178

7 Sahm, F. *et al.* DNA methylation-based classification and grading system for meningioma: a multicentre, retrospective analysis. *Lancet Oncol* **18**, 682-694 (2017). https://doi.org/10.1016/S1470-2045(17)30155-9

8 Choudhury, A. *et al.* Meningioma DNA methylation groups identify biological drivers and therapeutic vulnerabilities. *Nat Genet* **54**, 649-659 (2022). https://doi.org/10.1038/s41588-022-01061-8

9 Nassiri, F. *et al.* A clinically applicable integrative molecular classification of meningiomas. *Nature* **597**, 119-125 (2021). https://doi.org/10.1038/s41586-021-03850-3
10 Bayley, J. C. t. *et al.* Multiple approaches converge on three biological subtypes of meningioma and extract new insights from published studies. *Sci Adv* **8**, eabm6247 (2022). https://doi.org/10.1126/sciadv.abm6247
11 Lotsch, C. *et al.* Tumor-associated macrophages in meningiomas: a novel biomarker for poor survival outperforming the benefits of T cells. *Acta Neuropathol* **150**, 41 (2025). https://doi.org/10.1007/s00401-025-02948-6
12 Maas, S. L. N. *et al.* A microenvironment-determined risk continuum refines subtyping in meningioma and reveals determinants of machine learning-based tumor classification. *Nat Genet* **58**, 341-354 (2026). https://doi.org/10.1038/s41588-025-02475-w
13 Kotecha, R. S. *et al.* Meningiomas in children and adolescents: a meta-analysis of individual patient data. *Lancet Oncol* **12**, 1229-1239 (2011). https://doi.org/10.1016/S1470-2045(11)70275-3
14 Smith, M. J. *et al.* Loss-of-function mutations in SMARCE1 cause an inherited disorder of multiple spinal meningiomas. *Nat Genet* **45**, 295-298 (2013). https://doi.org/10.1038/ng.2552
15 Smith, M. J. *et al.* Germline SMARCE1 mutations predispose to both spinal and cranial clear cell meningiomas. *J Pathol* **234**, 436-440 (2014). https://doi.org/10.1002/path.4427
16 Shankar, G. M. *et al.* Germline and somatic BAP1 mutations in high-grade rhabdoid meningiomas. *Neuro Oncol* **19**, 535-545 (2017). https://doi.org/10.1093/neuonc/now235
17 Shankar, G. M. & Santagata, S. BAP1 mutations in high-grade meningioma: implications for patient care. *Neuro Oncol* **19**, 1447-1456 (2017). https://doi.org/10.1093/neuonc/nox094
18 Toland, A. *et al.* Pediatric meningioma: a clinicopathologic and molecular study with potential grading implications. *Brain Pathol* **30**, 1134-1143 (2020). https://doi.org/10.1111/bpa.12884
19 Battu, S. *et al.* Clinicopathological and molecular characteristics of pediatric meningiomas. *Neuropathology* **38**, 22-33 (2018). https://doi.org/10.1111/neup.12426
20 Sievers, P. *et al.* Clear cell meningiomas are defined by a highly distinct DNA methylation profile and mutations in SMARCE1. *Acta Neuropathol* **141**, 281-290 (2021). https://doi.org/10.1007/s00401-020-02247-2
21 Sievers, P. *et al.* YAP1-fusions in pediatric NF2-wildtype meningioma. *Acta Neuropathol* **139**, 215-218 (2020). https://doi.org/10.1007/s00401-019-02095-9
22 Szulzewsky, F. *et al.* Both YAP1-MAML2 and constitutively active YAP1 drive the formation of tumors that resemble NF2 mutant meningiomas in mice. *Genes Dev* **36**, 857-870 (2022). https://doi.org/10.1101/gad.349876.122
23 Maas, S. L. N. *et al.* Integrated Molecular-Morphologic Meningioma Classification: A Multicenter Retrospective Analysis, Retrospectively and Prospectively Validated. *J Clin Oncol* **39**, 3839-3852 (2021). https://doi.org/10.1200/JCO.21.00784
24 Dudley, R. W. R. *et al.* Pediatric versus adult meningioma: comparison of epidemiology, treatments, and outcomes using the Surveillance, Epidemiology, and End Results database. *J Neurooncol* **137**, 621-629 (2018). https://doi.org/10.1007/s11060-018-2756-1
25 Thuijs, N. B. *et al.* Pediatric meningiomas in The Netherlands 1974-2010: a descriptive epidemiological case study. *Childs Nerv Syst* **28**, 1009-1015 (2012). https://doi.org/10.1007/s00381-012-1759-z
26 Sill, M. *et al.* Advancing CNS tumor diagnostics with expanded DNA methylation-based classification. *Cancer Cell* (2025). https://doi.org/10.1016/j.ccell.2025.11.002
27 Berghaus, N. *et al.* Meningiomas: Sex-specific differences and prognostic implications of a chromosome X loss. *Neuro Oncol* **27**, 1019-1028 (2025). https://doi.org/10.1093/neuonc/noae239
28 Maas, S. L. N. *et al.* Loss over 5% of chromosome 1p is a clinically relevant and applicable cut-off for increased risk of recurrence in meningioma. *Acta Neuropathol* **148**, 17 (2024). https://doi.org/10.1007/s00401-024-02777-z
29 Driver, J. *et al.* A molecularly integrated grade for meningioma. *Neuro Oncol* **24**, 796-808 (2022). https://doi.org/10.1093/neuonc/noab213

30 Zhang, T. *et al.* Tumour-associated macrophage infiltration differs in meningioma genotypes, and is important in tumour dynamics. *J Exp Clin Cancer Res* **44**, 162 (2025). https://doi.org/10.1186/s13046-025-03419-2
31 Sievers, P. *et al.* Molecular signatures define BAP1-altered meningioma as a distinct CNS tumor with deregulation of Polycomb repressive complex target genes. *Neuro Oncol* **27**, 2326-2340 (2025). https://doi.org/10.1093/neuonc/noaf105
32 Landry, A. P. *et al.* Chromosome 1p Loss and 1q Gain for Grading of Meningioma. *JAMA Oncol* **11**, 644-649 (2025). https://doi.org/10.1001/jamaoncol.2025.0329
33 Pfister, S. M. *et al.* A Summary of the Inaugural WHO Classification of Pediatric Tumors: Transitioning from the Optical into the Molecular Era. *Cancer Discov* **12**, 331-355 (2022). https://doi.org/10.1158/2159-8290.CD-21-1094
34 Louis, D. N. *et al.* The 2021 WHO Classification of Tumors of the Central Nervous System: a summary. *Neuro Oncol* **23**, 1231-1251 (2021). https://doi.org/10.1093/neuonc/noab106
35 Sturm, D. *et al.* Multiomic neuropathology improves diagnostic accuracy in pediatric neuro-oncology. *Nat Med* **29**, 917-926 (2023). https://doi.org/10.1038/s41591-023-02255-1
36 Capper, D. *et al.* DNA methylation-based classification of central nervous system tumours. *Nature* **555**, 469-474 (2018). https://doi.org/10.1038/nature26000
37 Roy, R. *et al.* DNA methylation signatures reveal that distinct combinations of transcription factors specify human immune cell epigenetic identity. *Immunity* **54**, 2465-2480 e2465 (2021). https://doi.org/10.1016/j.immuni.2021.10.001
38 Reynolds, L. M. *et al.* Age-related variations in the methylome associated with gene expression in human monocytes and T cells. *Nat Commun* **5**, 5366 (2014). https://doi.org/10.1038/ncomms6366
39 Salas, L. A. *et al.* Enhanced cell deconvolution of peripheral blood using DNA methylation for high-resolution immune profiling. *Nat Commun* **13**, 761 (2022). https://doi.org/10.1038/s41467-021-27864-7
40 Jamil, M. A. *et al.* Molecular Analysis of Fetal and Adult Primary Human Liver Sinusoidal Endothelial Cells: A Comparison to Other Endothelial Cells. *Int J Mol Sci* **21** (2020). https://doi.org/10.3390/ijms21207776
41 Franzen, J. *et al.* Senescence-associated DNA methylation is stochastically acquired in subpopulations of mesenchymal stem cells. *Aging Cell* **16**, 183-191 (2017). https://doi.org/10.1111/acel.12544
42 Hautefort, A. *et al.* Pulmonary endothelial cell DNA methylation signature in pulmonary arterial hypertension. *Oncotarget* **8**, 52995-53016 (2017). https://doi.org/10.18632/oncotarget.18031
43 Holm, K. *et al.* An integrated genomics analysis of epigenetic subtypes in human breast tumors links DNA methylation patterns to chromatin states in normal mammary cells. *Breast Cancer Res* **18**, 27 (2016). https://doi.org/10.1186/s13058-016-0685-5
44 Kular, L. *et al.* DNA methylation changes in glial cells of the normal-appearing white matter in Multiple Sclerosis patients. *Epigenetics* **17**, 1311-1330 (2022). https://doi.org/10.1080/15592294.2021.2020436
45 Jung, N., Dai, B., Gentles, A. J., Majeti, R. & Feinberg, A. P. An LSC epigenetic signature is largely mutation independent and implicates the HOXA cluster in AML pathogenesis. *Nat Commun* **6**, 8489 (2015). https://doi.org/10.1038/ncomms9489
46 de Witte, L. D. *et al.* Contribution of Age, Brain Region, Mood Disorder Pathology, and Interindividual Factors on the Methylome of Human Microglia. *Biol Psychiatry* **91**, 572-581 (2022). https://doi.org/10.1016/j.biopsych.2021.10.020
47 Moss, J. *et al.* Comprehensive human cell-type methylation atlas reveals origins of circulating cell-free DNA in health and disease. *Nat Commun* **9**, 5068 (2018). https://doi.org/10.1038/s41467-018-07466-6
48 Kozlenkov, A. *et al.* Differences in DNA methylation between human neuronal and glial cells are concentrated in enhancers and non-CpG sites. *Nucleic Acids Res* **42**, 109-127 (2014). https://doi.org/10.1093/nar/gkt838

49 Pai, S. *et al.* Differential methylation of enhancer at IGF2 is associated with abnormal dopamine synthesis in major psychosis. *Nat Commun* **10**, 2046 (2019). https://doi.org/10.1038/s41467-019-09786-7
50 Kennedy, D. W. *et al.* Critical evaluation of linear regression models for cell-subtype specific methylation signal from mixed blood cell DNA. *PLoS One* **13**, e0208915 (2018). https://doi.org/10.1371/journal.pone.0208915
51 Salas, L. A. *et al.* An optimized library for reference-based deconvolution of whole-blood biospecimens assayed using the Illumina HumanMethylationEPIC BeadArray. *Genome Biol* **19**, 64 (2018). https://doi.org/10.1186/s13059-018-1448-7
52 Johann, P. D., Jager, N., Pfister, S. M. & Sill, M. RF_Purify: a novel tool for comprehensive analysis of tumor-purity in methylation array data based on random forest regression. *BMC Bioinformatics* **20**, 428 (2019). https://doi.org/10.1186/s12859-019-3014-z
53 Chakravarthy, A. *et al.* Pan-cancer deconvolution of tumour composition using DNA methylation. *Nat Commun* **9**, 3220 (2018). https://doi.org/10.1038/s41467-018-05570-1
54 Greenacre, M. J. *Compositional Data Analysis*. (2021).
55 K. Gerald van den Boogaart, R. T. D. *Analysing compositional Data with R, UseR!* . (Springer 2013).
56 Filzmoser, P., Hron, K., Templ, M. *Applied compositional data analysis. With worked examples in R*. (Springer Cham., 2018).
57 Sahm, F. *et al.* Next-generation sequencing in routine brain tumor diagnostics enables an integrated diagnosis and identifies actionable targets. *Acta Neuropathol* **131**, 903-910 (2016). https://doi.org/10.1007/s00401-015-1519-8
58 van Buuren, S., Groothuis-Oudshoorn, K. mice: Multivariate Imputation by Chained Equations in R. *Journal of Statistical Software* **45**, 1.67 (2011). https://doi.org/doi:10.18637/jss.v045.i03
59 Grambsch, P., Therneau, T. Proportional hazards tests and diagnostics based on weighted residuals. *Biometrika*, 515-526 (1994).
