# Supplementary material for: Distinct molecular subgroups in pediatric and young-onset meningiomas require age-adapted risk stratification
Source: Nat Commun. 2026 Jul 14;17:6188. doi: 10.1038/s41467-026-75357-2 (PMC13369989; doi:10.1038/s41467-026-75357-2)
Supplement: Supplementary file 2 — Reporting Summary [file 41467_2026_75357_MOESM2_ESM.pdf]

nature portfolio

Corresponding author(s): Philipp Sievers
Last updated by author(s): Jun 23, 2026

Reporting Summary

Nature Portfolio wishes to improve the reproducibility of the work that we publish. This form provides structure for consistency and transparency in reporting. For further information on Nature Portfolio policies, see our Editorial Policies and the Editorial Policy Checklist.

Statistics

For all statistical analyses, confirm that the following items are present in the figure legend, table legend, main text, or Methods section.

n/a	Confirmed	
☐	☒	The exact sample size (n) for each experimental group/condition, given as a discrete number and unit of measurement
☐	☒	A statement on whether measurements were taken from distinct samples or whether the same sample was measured repeatedly
☐	☒	The statistical test(s) used AND whether they are one- or two-sided<br>*Only common tests should be described solely by name; describe more complex techniques in the Methods section.*
☐	☒	A description of all covariates tested
☐	☒	A description of any assumptions or corrections, such as tests of normality and adjustment for multiple comparisons
☐	☒	A full description of the statistical parameters including central tendency (e.g. means) or other basic estimates (e.g. regression coefficient) AND variation (e.g. standard deviation) or associated estimates of uncertainty (e.g. confidence intervals)
☐	☒	For null hypothesis testing, the test statistic (e.g. F, t, r) with confidence intervals, effect sizes, degrees of freedom and P value noted<br>*Give P values as exact values whenever suitable.*
☒	☐	For Bayesian analysis, information on the choice of priors and Markov chain Monte Carlo settings
☐	☒	For hierarchical and complex designs, identification of the appropriate level for tests and full reporting of outcomes
☐	☒	Estimates of effect sizes (e.g. Cohen's d, Pearson's r), indicating how they were calculated

Our web collection on statistics for biologists contains articles on many of the points above.

Software and code

Policy information about availability of computer code

Data collection	Data were collected using a custom-designed database to securely manage patient and molecular profiling information across collaborating centers. Publicly available DNA methylation data from the Gene Expression Omnibus (GEO) were also incorporated for deconvolution analysis.
Data analysis	Data analysis was performed using R and Python. Statistical tests (Fisher's exact, Wilcoxon, Kaplan-Meier, Cox regression) assessed clinical and molecular differences. For genomic and epigenomic analysis, conumee was used for CNV analysis, while MethylCybersort was employed for methylation deconvolution. We used publicly available methylation datasets (e.g., GSE184269, GSE167998) to construct a cell-type reference matrix for deconvolution. T-SNE analysis was carried out using the Python openTSNE package (v1.0.2). MIKAYA was used for the assessment of the H Score for the CD68 staining. For the evaluation of nuclear progesterone receptor (PR) expression, a semi-automated custom cell-detection workflow was employed. DNA sequence data were aligned to the human reference genome GRCh37 (hg19) using the Burrows–Wheeler Aligner (BWA). For RNA sequencing, demultiplexed FASTQ files were generated using Illumina bclconvert (v3.10.5), and reads were aligned to the Gencode GRCh37 primary assembly using STAR (v2.7.7a). Gene fusion events were identified using Arriba (v2.4.0), focusing on high-confidence fusion calls.

For manuscripts utilizing custom algorithms or software that are central to the research but not yet described in published literature, software must be made available to editors and reviewers. We strongly encourage code deposition in a community repository (e.g. GitHub). See the Nature Portfolio guidelines for submitting code & software for further information.

Data

Policy information about availability of data

All manuscripts must include a data availability statement. This statement should provide the following information, where applicable:

- Accession codes, unique identifiers, or web links for publicly available datasets
- A description of any restrictions on data availability
- For clinical datasets or third party data, please ensure that the statement adheres to our policy

DNA methylation data generated in this study have been deposited in the NCBI Gene Expression Omnibus (GEO) under accession code GSE317578 (https://www.ncbi.nlm.nih.gov/geo/query/acc.cgi?acc=GSE317578). Public DNA methylation datasets used for deconvolution analyses were obtained from GEO under accession codes GSE50798 (https://www.ncbi.nlm.nih.gov/geo/query/acc.cgi?acc=GSE50798), GSE56581(https://www.ncbi.nlm.nih.gov/geo/query/acc.cgi?acc=GSE56581), GSE63409 (https://www.ncbi.nlm.nih.gov/geo/query/acc.cgi?acc=GSE63409), GSE74877 (https://www.ncbi.nlm.nih.gov/geo/query/acc.cgi?acc=GSE74877), GSE82234 (https://www.ncbi.nlm.nih.gov/geo/query/acc.cgi?acc=GSE82234), GSE84395 (https://www.ncbi.nlm.nih.gov/geo/query/acc.cgi?acc=GSE84395), GSE88824 (https://www.ncbi.nlm.nih.gov/geo/query/acc.cgi?acc=GSE88824), GSE110554 (https://www.ncbi.nlm.nih.gov/geo/query/acc.cgi?acc=GSE110554), GSE110555 (https://www.ncbi.nlm.nih.gov/geo/query/acc.cgi?acc=GSE110555), GSE112179 (https://www.ncbi.nlm.nih.gov/geo/query/acc.cgi?acc=GSE112179), GSE122126 (https://www.ncbi.nlm.nih.gov/geo/query/acc.cgi?acc=GSE122126), GSE140295 (https://www.ncbi.nlm.nih.gov/geo/query/acc.cgi?acc=GSE140295), GSE166207 (https://www.ncbi.nlm.nih.gov/geo/query/acc.cgi?acc=GSE166207), GSE167998 (https://www.ncbi.nlm.nih.gov/geo/query/acc.cgi?acc=GSE167998), GSE184269 (https://www.ncbi.nlm.nih.gov/geo/query/acc.cgi?acc=GSE184269) and GSE191200 (https://www.ncbi.nlm.nih.gov/geo/query/acc.cgi?acc=GSE191200).

Raw targeted DNA and RNA sequencing data contain potentially identifiable human genetic information and are therefore subject to the conditions of the ethics approvals granted by the Ethics Committee of the Medical Faculty Heidelberg, Heidelberg University (S-318/2022 and S-224/2024), as well as the requirements of the European General Data Protection Regulation (GDPR). The informed consent framework for this retrospective study does not permit unrestricted public sharing of individual-level genomic data. Consequently, raw sequencing data are not publicly available. Filtered sequencing data will be made available for non-commercial research purposes upon approval of a data access request and completion of a data transfer agreement with the corresponding author. These data will remain available for at least 10 years following publication. Requests will be acknowledged within 14 days. Source data are provided with this paper. All other data supporting the findings of this study are available within the Article and Supplementary Information.

Research involving human participants, their data, or biological material

Policy information about studies with human participants or human data. See also policy information about sex, gender (identity/presentation), and sexual orientation and race, ethnicity and racism.

Reporting on sex and gender	Information on sex (biological attribute) was collected during the study. No information on gender was collected for the patients.
Reporting on race, ethnicity, or other socially relevant groupings	No information on race, ethnicity, or other socially relevant groupings was collected.
Population characteristics	Sample selection was based on an age cutoff of ≤39 years, documentation of patient sex, and the availability of DNA sequencing and DNA methylation data with a meningioma score ≥0.8 in the Heidelberg DNA methylation classifier (V12.8). Additional considerations for subgroup analyses included histopathological subtype, WHO grade, and tumor location.
Recruitment	Patients included in this study were retrospectively identified from multiple collaborating centers across Germany, the Netherlands, France, Italy, Turkey, Spain, and the United States. All patients had meningiomas diagnosed before the age of 40. Recruitment was based on the availability of clinical data, including age, sex, and tumor sample suitable for DNA sequencing and DNA methylation profiling. Data from these patients were collected and analyzed from existing tumor registries or clinical databases at the collaborating centers.
Ethics oversight	The study was conducted in accordance with the ethics approval of the University of Heidelberg (S-318/2022; S-224/2024). Due to the retrospective design of this study, informed consent was waived, and all patient data were pseudonymized before analysis.

Note that full information on the approval of the study protocol must also be provided in the manuscript.

Field-specific reporting

Please select the one below that is the best fit for your research. If you are not sure, read the appropriate sections before making your selection.

☒ Life sciences ☐ Behavioural & social sciences ☐ Ecological, evolutionary & environmental sciences

For a reference copy of the document with all sections, see nature.com/documents/nr-reporting-summary-flat.pdf

Life sciences study design

All studies must disclose on these points even when the disclosure is negative.

Sample size	N=293
Data exclusions	Data not fullfilling the QC criteria were excluded.

Replication	The cohort comprisesd samples from multiple international centers to ensure robustness.
Randomization	This study was retrospective and did not involve randomization.
Blinding	Blinding was not relevant to the genomic data and histopathological analysis, as investigator bias would not affect the data output.

Reporting for specific materials, systems and methods

We require information from authors about some types of materials, experimental systems and methods used in many studies. Here, indicate whether each material, system or method listed is relevant to your study. If you are not sure if a list item applies to your research, read the appropriate section before selecting a response.

Materials & experimental systems

n/a	Involved in the study
☐	☒ Antibodies
☒	☐ Eukaryotic cell lines
☒	☐ Palaeontology and archaeology
☒	☐ Animals and other organisms
☒	☐ Clinical data
☒	☐ Dual use research of concern
☒	☐ Plants

Methods

n/a	Involved in the study
☒	☐ ChIP-seq
☒	☐ Flow cytometry
☒	☐ MRI-based neuroimaging

Antibodies

Antibodies used	CD68 antibody (Dako M0876, clone PG-M1), PR antibody (Biogenex Laboratories, clone PR88), and ER antibody (Thermo Scientific, clone SP1).
Validation	All primary antibodies used in this study were validated by the manufacture.

Plants

Seed stocks	*Report on the source of all seed stocks or other plant material used. If applicable, state the seed stock centre and catalogue number. If plant specimens were collected from the field, describe the collection location, date and sampling procedures.*
Novel plant genotypes	*Describe the methods by which all novel plant genotypes were produced. This includes those generated by transgenic approaches, gene editing, chemical/radiation-based mutagenesis and hybridization. For transgenic lines, describe the transformation method, the number of independent lines analyzed and the generation upon which experiments were performed. For gene-edited lines, describe the editor used, the endogenous sequence targeted for editing, the targeting guide RNA sequence (if applicable) and how the editor was applied.*
Authentication	*Describe any authentication procedures for each seed stock used or novel genotype generated. Describe any experiments used to assess the effect of a mutation and, where applicable, how potential secondary effects (e.g. second site T-DNA insertions, mosiacism, off-target gene editing) were examined.*
