# Supplementary material for: Distinct molecular subgroups in pediatric and young-onset meningiomas require age-adapted risk stratification
Source: Nat Commun. 2026 Jul 14;17:6188. doi: 10.1038/s41467-026-75357-2 (PMC13369989; doi:10.1038/s41467-026-75357-2)
Supplement: Supplementary file 3 — Transparent Peer Review file [file 41467_2026_75357_MOESM3_ESM.pdf]

nature portfolio

Peer Review File

Distinct molecular subgroups in pediatric and young-onset meningiomas require age-adapted risk stratification

Corresponding Author: Dr Philipp Sievers

This file contains all reviewer reports in order by version, followed by all author rebuttals in order by version.

Version 0:

Reviewer comments:

Reviewer #1

(Remarks to the Author)
Extent of resection (EOR) definition is under-specified.
“GTR/STR” is used as a prognostic factor, yet the manuscript does not provide a reproducible operational definition (e.g., Simpson grade I–III vs >III, early postoperative MRI criteria, surgeon report). Given the known strong association of EOR with recurrence/progression in meningioma, lack of a clear definition (and handling of discordant Simpson vs MRI) undermines any downstream claims of “independent” molecular prognostic value.
Adjuvant radiotherapy is not clearly defined and may be misclassified.
Radiotherapy should be explicitly classified as adjuvant (pre-progression) versus salvage (post-recurrence) and timing should be stated (e.g., within 3–6 months after index surgery). Without intent/timing, RT becomes confounded by indication and can distort survival associations.
The survival modeling framework appears incomplete for causal/prognostic statements.
The current analysis relies predominantly on univariable associations (Kaplan–Meier/log-rank and univariable Cox). If the manuscript aims to state that molecular features are prognostic markers, it should demonstrate independence from established clinical determinants (at minimum: WHO grade, age, EOR, and RT intent) using multivariable Cox, with proportional hazards checks and attention to events-per-variable to avoid overfitting. If multivariable modeling is not feasible due to limited events/missingness, conclusions must be downgraded to exploratory and phrased accordingly.
Over-strong language / focus imbalance.
The discussion currently foregrounds molecular items as prognostic markers while the evaluation of classical clinical predictors (EOR, RT) is comparatively weak. Given the clinical reality that EOR (and treatment selection for RT) often dominate progression risk, the manuscript should either (a) strengthen and transparently define/adjust these clinical variables, or (b) explicitly acknowledge that molecular findings are hypothesis-generating and potentially confounded by treatment and surgical factors.

Reviewer #2

(Remarks to the Author)
This is a high-quality, conceptually impactful study that significantly advances understanding of pediatric and AYA meningioma biology. The depth of multi-omic integration and the consistency of the findings convincingly establish young-onset meningiomas as a biologically distinct disease entity requiring age-adapted risk stratification. The work has clear implications for future WHO classifications, diagnostic frameworks, and clinical management, and constitutes a substantial contribution to the field. The minor limitations inherent to retrospective, multi-institutional studies and small numbers in rare subgroups. Overall, this manuscript represents a significant contribution to the field and is highly suitable for publication.

Manuscript Strengths
1. The study represents one of the largest and best-curated cohorts of pediatric and AYA meningiomas, providing robust and generalizable conclusions.
2. The manuscript convincingly shows that adult prognostic frameworks (WHO grade, methylation class, integrated risk scores, chr1p loss) fail in patients ≤21 years, addressing a major unmet need.
3. Integration of histopathology, methylation profiling, CNV analysis, and sequencing reveals a distinct, gain-dominated CNV landscape (notably chr17q gain) and enables clinically meaningful, driver-based subgrouping.
4. The consistent prognostic importance of extent of resection across molecular subgroups and the proposed age-adapted

risk stratification model provide clear, practice-relevant guidance.

Minor Limitations
1. Progression-free survival and germline status are available only for subsets of patients, limiting power in some subgroup analyses.
2. Low case numbers for BAP1-altered and YAP1-fusion tumors restrict definitive prognostic conclusions.
3. While adult methylation classes show limited utility, a fully validated pediatric-specific classifier is not yet proposed.

Reviewer #3

(Remarks to the Author)
This study provides a valuable large-scale integrated characterization of youth-onset meningiomas. However, key conclusions regarding the biological distinctness of tumors ≤21 years and the proposed hierarchical risk model require more consistent age stratification, additional analyses, and stronger statistical support before acceptance. I suggest major revision.
Here are my major and minor concerns:

Major Comments:
1. Inconsistent and shifting age stratification
While the manuscript initially defines two stratification approaches (0–14 vs 15–39 and ≤21 vs >21), the analyses and figures inconsistently switch between these groupings, making it difficult to follow and weakening the claim that ≤21 years represents a distinct biological entity:
• Figure 1B–D: analysis restricted to 0–21 years
• Figure 1E: entire 0–39 cohort
• Figure 1F: restricted to 0–14 years
• Figure 5: conclusions based primarily on 0–21 years
• Figure 5B: age group definition not clearly stated
If the central conclusion is that tumors arising ≤21 years constitute a biologically distinct entity, then:
• Key CNV and survival analyses should be consistently shown for the 0–21 group.
• Figure 1F CNV landscape should be shown for 0–21, not only 0–14.
o and possibly for 21-39 to see/show that they are “more similar” to adult cohort.
• Figure 1E (chr1p survival) should be repeated specifically for 0–21.
• The 21–39 group must be analyzed directly rather than inferred.

The 0-21 age threshold appears data-driven but insufficiently justified analytically (specifically for 21-39 samples).

Additionally, the title: “Integrated, hierarchical risk stratification reveals distinct prognostic subgroups in pediatric and AYA meningiomas” is inconsistent with the fact that the stratification model applies only to ≤21 and AYA refers to 15-39.
The authors should clarify whether the model applies to:
• PED only?
• PED + AYA?
• ≤21 only?

2. The 21–39 age group is not characterized; it is not clear how they conclude that >21 converges to adult cohort.
The Discussion proposes that tumors in the 21–39 age group progressively resemble adult disease. However:
• There is no focused molecular or survival analysis specifically on 21–39.
• Figure 4 and the hierarchical model apply only to ≤21.
• It remains unclear whether adult prognostic frameworks perform adequately in 21–39.

3. Germline status requires more rigorous integration
Germline status of this cohort is a very important factor and should be clarified/addressed earlier, and many (if not all) of the analysis should be performed based on germline status, either by excluding the cases with known predisposing germline variants, or by correcting for it. Here are a few related questions/concerns:
• Why are only germline NF2 mutations considered?
• Should analyses be repeated excluding known predisposition cases?
• Do germline NF2 tumors cluster together in methylation space?

4. TME deconvolution analysis needs further stratification
The deconvolution analysis (Figure 2C, 3B) shows differences between youth and adult tumors, and between driver-defined groups.
However:
• Analyses comparing PED vs AYA should be repeated within methylation clusters to determine whether differences are age-driven or cluster-driven.
• Driver-based TME comparisons (NF2 vs SMARCE1 vs BAP1) should be repeated in >40 patients where possible to determine whether differences are age-specific or driver-specific.
• Statistical metrics (adjusted p-values) should be clearly annotated on Figures 2C and 3B.
Currently, this analysis seems immature and can be further evaluated.

5. Several statements lack statistical support
For example:
“Cluster 4 consisted of SMARCE1-altered meningiomas and cluster 3 contained BAP1-altered meningiomas. Cluster 1 and area B were enriched for NF2-mutated meningiomas.”
Please provide enrichment statistics (Fisher’s exact p-values).
Similarly, several descriptive claims regarding methylation clustering and driver enrichment should include formal statistical support.
Many of the statistical tests heavily on univariable analysis. Multivariable models should be used to test for test age, driver, CNV, EOR together?

Minor Comments
1. The first sentence of “Driver alterations influence the epigenetic clustering” section is unclear and could be revised.
2. The term “reference cohort” for adult meningiomas is confusing, especially in methylation analysis. Consider replacing with “adult comparison cohort.”
3. In Figure 1A, inclusion of mutational status (NF2, BAP1, SMARCE1, YAP1, germline) and CNV status (chr17p, 1p etc.) would strengthen integration of molecular findings.
4. Several figures lack explicit definition of age groups in legends.

Version 1:

Reviewer comments:

Reviewer #1

(Remarks to the Author)
Thank you for the careful revision and detailed rebuttal. The manuscript is substantially improved and now clinically much more convincing. In particular, you clearly define EOR using surgeon-reported Simpson grading (I–III vs IV–V) , strengthen the survival analysis with an adjusted Cox model (including Firth correction, imputation and PH checks) , and appropriately present EOR as the dominant prognostic determinant in patients ≤21 years . The radiotherapy section is also handled cautiously with transparent acknowledgement of confounding by indication .

I am happy to recommend acceptance, with only minor editorial polishing to ensure the abstract/discussion consistently frame molecular stratification as risk refinement in the context of strong clinical determinants (especially EOR).

Reviewer #2

(Remarks to the Author)
The authors have provided a detailed and satisfactory point-by-point response to the reviewers’ concerns. Most of the major issues raised during the previous round of review have been appropriately addressed, including clarification of clinical variables, refinement of the analytical framework, and suitable tempering of the conclusions.

In my opinion, the revised manuscript is substantially improved and now meets the standards required for publication. I therefore recommend acceptance of the manuscript.

Reviewer #3

(Remarks to the Author)
I commend the authors for their thorough and detailed responses. They have fully addressed my concerns. I only have a few minor suggestions/questions:
1. “homozygote deletions” in line 292 should be updated to “homozygous deletions”
2. Progesterone receptor (PR) staining H-score is initially mentioned to be significantly different between female and male cases in 0-21 age group. Later, in “Driver alterations shape tumor composition” section, authors indicate that NF2 vs SMARCE1 group also showed a difference in PR staining. It is not clear whether this analysis was performed by correcting for gender.

Open Access This Peer Review File is licensed under a Creative Commons Attribution 4.0 International License, which permits use, sharing, adaptation, distribution and reproduction in any medium or format, as long as you give appropriate credit to the original author(s) and the source, provide a link to the Creative Commons license, and indicate if changes were made.
In cases where reviewers are anonymous, credit should be given to 'Anonymous Referee' and the source.
The images or other third party material in this Peer Review File are included in the article's Creative Commons license, unless indicated otherwise in a credit line to the material. If material is not included in the article's Creative Commons license and your intended use is not permitted by statutory regulation or exceeds the permitted use, you will need to obtain permission directly from the copyright holder.
To view a copy of this license, visit https://creativecommons.org/licenses/by/4.0/

Point-by-point response to the reviewers' comments:

Manuscript: "Distinct molecular subgroups in pediatric and young-onset meningiomas require age-adapted risk stratification"

<u>Reviewer #1</u>

Extent of resection (EOR) definition is under-specified.
"GTR/STR" is used as a prognostic factor, yet the manuscript does not provide a reproducible operational definition (e.g., Simpson grade I–III vs >III, early postoperative MRI criteria, surgeon report). Given the known strong association of EOR with recurrence/progression in meningioma, lack of a clear definition (and handling of discordant Simpson vs MRI) undermines any downstream claims of "independent" molecular prognostic value.

<u>Response:</u> We sincerely thank the reviewer for this important and constructive comment. In the revised manuscript, we have explicitly defined the extent of resection (EOR) using the Simpson grading system as the primary classification framework (GTR: Simpson I–III vs STR: Simpson IV–V). Due to the multicenter retrospective design, systematic early postoperative MRI data were not consistently available; therefore, EOR classification was based on surgeon-reported Simpson grade, as now clearly specified in the Methods section.

<u>Addition / Modifications:</u>

- We included a new paragraph in the Methods section (subheadline: "Patient samples and ethical approval"):
  "Extent of resection (EOR) was defined using the surgeon-reported Simpson grading system, with gross total resection (GTR) classified as Simpson grades I–III and subtotal resection (STR) as Simpson grades IV–V."
- We restructured the results part to highlight the impact of clinical variables (EOR and RT) and introduced included a new chapter under the subheadline: "*Surgical extent as primary prognostic factor within an age-adapted stratification framework*"
- We adjusted our risk model for 0-21-year-old patients for EOR (as well as for sex and WHO grade) and Intermediate risk remained a significant risk factor (results section, subheadline: "Integrated, hierarchical risk stratification reveals distinct prognostic subgroups in pediatric and AYA meningiomas"):
  "When applying the risk stratification displayed in Figure 5A to the presented cohort of 0-21 years of age patients, only low (n = 48) and intermediate (n = 48) cases can be compared since there are no BAP1 altered cases in this group. In a multivariable Cox PH model including risk stratification, extent of resection, WHO grade and sex, only intermediate risk (HR: 2.5, 95%CI 1.2-5.2, p = 0.02) and extent of resection (Simpson grade 4-5, HR: 2.9, 95%CI 1.3-6.1, p = 0.001) remained significant risk factors (SI Table 8)."
- We adapted the Discussion and now state:
  "However, within the pediatric-associated subgroup (0-<21 years), extent of resection remained the strongest predictor of progression across molecular categories. This observation emphasizes that classical clinical factors, particularly extent of resection, remain the dominant determinants of outcome in this cohort and must be considered when interpreting molecular associations." [...]
  "In addition, the retrospective design and variability in clinical management - including surgical approach and use of radiotherapy - introduce potential confounding that cannot be fully addressed without prospective validation, particularly given incomplete data on radiotherapy timing and intent and the limited event numbers available for multivariable modeling. Independent cohorts and prospective studies will therefore be important to confirm the prognostic relevance of the molecular patterns identified here." [...]

“While the extent of resection remains a principal determinant of outcome in tumors arising in younger patients, our findings suggest that molecular stratification can provide additional biological context that refine risk assessment in younger patients”

- We included Figure panel 5 B to show the impact of the EOR in the driver groups “*NF2*-altered" and “other”
- We adapted the Figure panel 5 C and D to include Simpson grade 1-3 vs 4-5 instead of GTR and STR

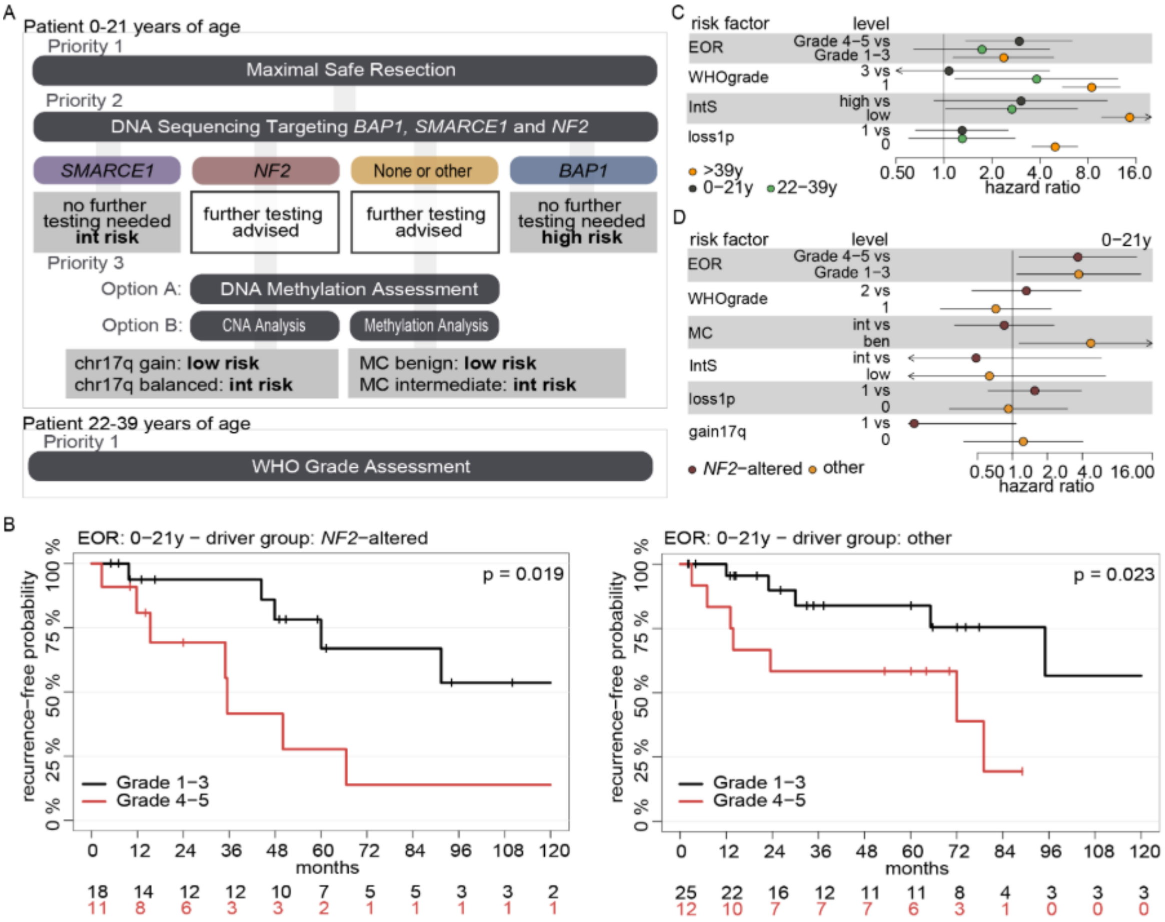

Figure 5. (A) Workflow for efficient meningioma evaluation in patients aged 0–21 years in diagnostic routine. Maximal safe resection remains the highest priority for reducing recurrence risk, and detailed documentation of the extent of resection is essential for accurately assessing and defining the individual risk of recurrence. As a second priority, DNA panel sequencing should be performed to detect alterations in *SMARCE1, BAP1* and *NF2*. Meningiomas harboring *SMARCE1* or *BAP1* alterations do not require additional molecular testing and can be stratified as intermediate-risk and high-risk for recurrence, respectively. For *NF2*-altered meningiomas and meningiomas without alterations in *SMARCE1, BAP1* or *NF2*, further molecular workup can follow two alternative paths: Option A (feasible for both subgroups): Methylation data can be generated using, for example, nanopore sequencing or methylation array profiling, allowing the simultaneous assessment of copy-number variation and methylation class. Option B (subgroup-specific): For *NF2*-altered cases, targeted analysis of chromosome 17q copy-number status (e.g., via FISH) can be performed, while for *NF2*-wild-type / other cases, methylation profiling alone is sufficient to enable subsequent stratification into low- and intermediate-risk groups. CNA: copy number alteration, in = intermediate, MC = methylation class, chr = chromosome. For patients aged 22–39 years, the WHO graduation has the highest priority. (B) Kaplan–Meier analysis for the patient group 0-21 years of age for the driver groups “*NF2*-altered” and “other”, stratified for extent of resection Simpson grade 1-3 and 4-5. EOR = extend of resection, y = years, The p-values were calculated using the Log-rank test. (C) Summary of risk factors and their corresponding hazard ratios for the age groups 0-21 years, 22-39 years and >39 years. EOR = extend of resection based on Simpson Grading, IntS = Integrated meningioma risk score by Maas et al. (2021), y = years. (D) Summary of risk factors and their corresponding hazard ratios in *NF2* altered meningiomas and “other” meningioma (0-21 years). The “other subgroup also contains meningiomas with unknown *BAP1* status. EOR = extend of resection based on Simpson Grading, MC = methylation class, ben = benign, int = intermediate, mal = malignant, IntS = Integrated meningioma risk score, y = years.

- We included EOR based on Simpson Grading in Figure 4 C

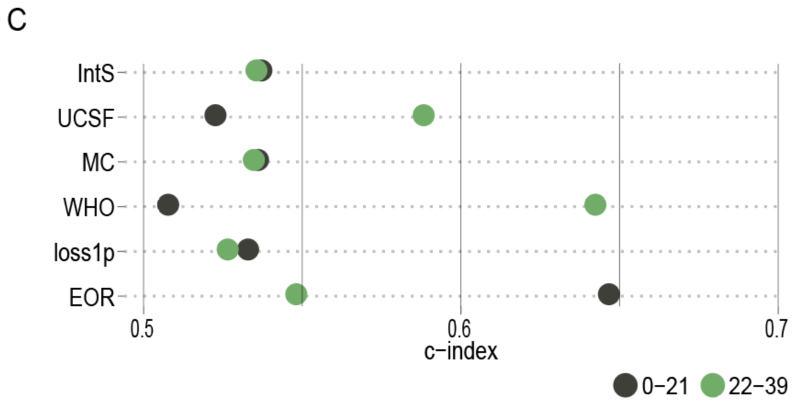

Figure 4 [...] (C) Comparison of the c-index among the patient groups 0-21-years-ols and 22-39-years-old across different stratification systems. IntS = Integrated meningioma risk score, UCSF = UCSF methylation classes, MC = Heidelberg methylation classes, WHO = WHO grades, loss1p = loss of chromosome 1p, EOR = Extend of resection based on Simpson Grading. [...]

- We included EOR based on Simpson Grading in SI Figure 4 A-D

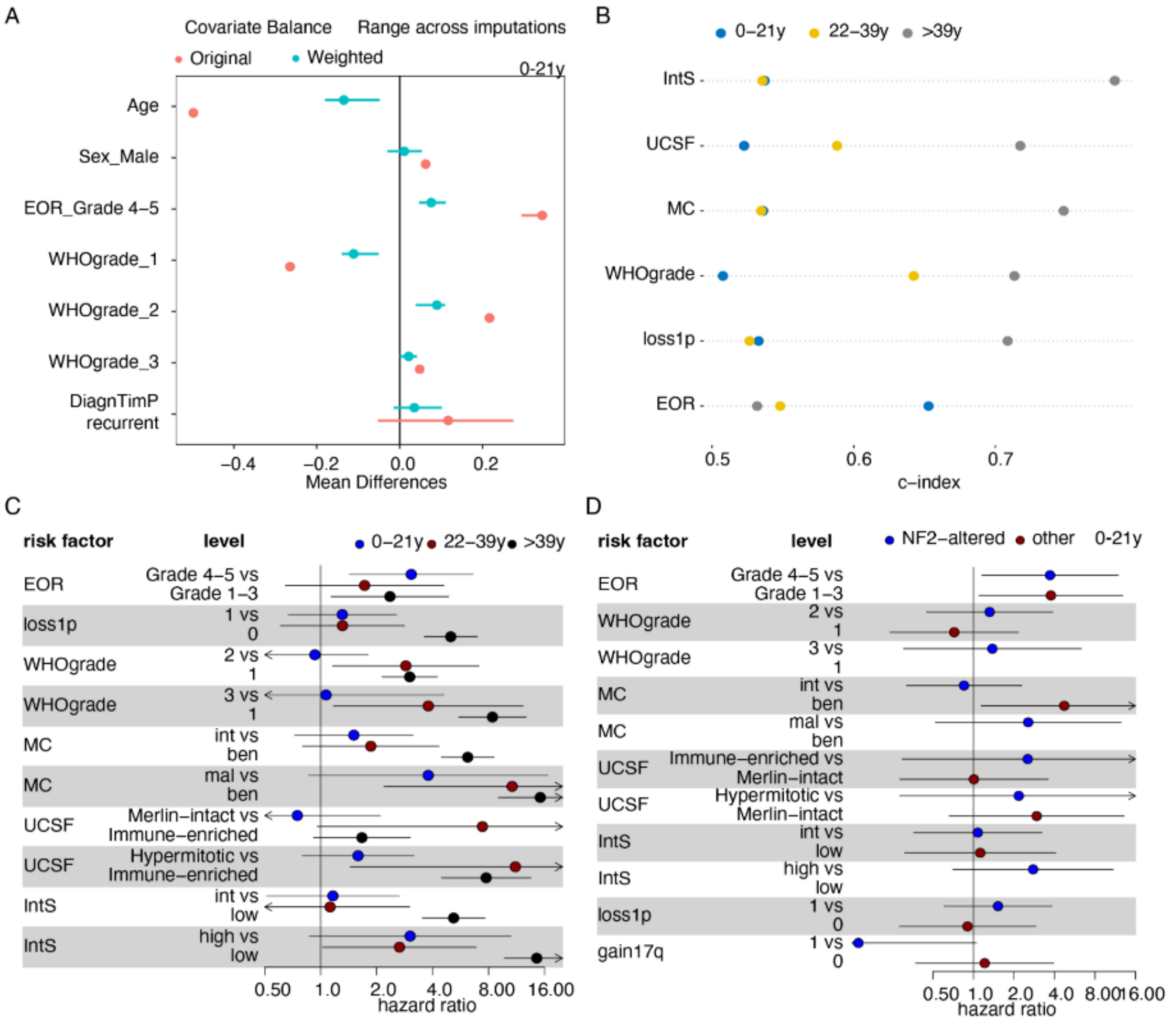

SI Figure 4. (A) ‘love’ plot showing the covariate balance between patients receiving/not receiving radiotherapy before and after propensity score (PS) weighting across imputed data sets in patients 0-21years. EOR = extend of resection based on Simpson grading. DiagnTimP = Timepoint of Diagnosis. (B) Comparison of the c-index among the patient groups 0-21-years-ols and 22-39-years-old and patients > 39 years of age across different stratification systems. IntS = Integrated meningioma risk score, UCSF = UCSF methylation classes, MC =

Heidelberg methylation classes, WHO = WHO grades, loss1p = loss of chromosome 1p, EOR = Extend of resection based on Simpson Grading. (C) Summary of risk factors and their corresponding hazard ratios between patients 0-21, 22-39 and >39 years of age. GTR = gross total resection, STR = subtotal resection, MC = methylation class, ben = benign, int = intermediate, mal = malignant, CNA = copy number alterations, IntS = Integrated meningioma risk score. (D) Summary of risk factors and their corresponding hazard ratios between meningiomas from patients 0-21 years of age with *NF2* alterations and the "other" subgroup, including or excluding cases presenting solely a loss of chromosome 22q. EOR = extend of resection, MC = methylation class, int = intermediate, mal = malignant, CNA = copy number alterations, IntS = Integrated meningioma risk score.

- We included EOR based on Simpson Grading in SI Table 1

SI Table 1. Overview and comparison of cohort characteristics across different age groups. [...]

	0-21y, n = 212	22-39y, n = 81	>39y, n = 747
[…]			
Simpson Grade 1-3	69 (69.0%)	46 (80.7%)	335 (93.6%)
Simpson Grade 4-5	31 (31.0%)	11 (19.3%)	23 (6.4%)
Simpson Grade Missing	112	24	389
[…]			

- We newly introduced SI Table 7, indicating the patients with available information on EOR based on Simpson Grading, radiotherapy and WHO grade

SI Table 7. Information on available radiotherapy data in the age groups 0-21 years and 22-39 years. EOR = extend of resection according to Simpson grading. Adj. = adjuvant, RT = radiotherapy.

	0-21 years	0-21 years	22-39 years	22-39 years
	No (n=26)	Adj. RT (n=16)	No (n=30)	Adj. RT (n=11)
EOR				
Grade1-3	19 (73.1%)	6 (40.0%)	28 (93.3%)	4 (36.4%)
Grade4-5	7 (26.9%)	9 (60.0%)	2 (6.7%)	7 (63.6%)
Missing	0	1	0	0
WHO grade				
1	15 (57.7%)	5 (31.3%)	21 (70.0%)	3 (27.3%)
2	9 (34.6%)	9 (56.3%)	9 (30.0%)	7 (63.6%)
3	2 (7.7%)	2 (12.5%)	0	1 (9.1%)

Adjuvant radiotherapy is not clearly defined and may be misclassified.
Radiotherapy should be explicitly classified as adjuvant (pre-progression) versus salvage (post-recurrence) and timing should be stated (e.g., within 3–6 months after index surgery). Without intent/timing, RT becomes confounded by indication and can distort survival associations.

Response: We thank the reviewer for this important comment and agree that clear definition of radiotherapy timing and intent is essential. In the revised manuscript, radiotherapy was defined as adjuvant treatment administered prior to documented progression following index surgery. Due to the retrospective multicenter design, additional details on timing (e.g., a fixed postoperative interval) and information on salvage radiotherapy were not consistently available. This has been clarified in the Methods section. We further acknowledge that patients receiving adjuvant radiotherapy more frequently presented with higher WHO grade and less complete resections (higher Simpson grade), as expected. After adjustment for these confounders, adjuvant radiotherapy remained associated with a non-significantly increased risk in both multivariable and propensity score–weighted analyses. This likely reflects residual

confounding and selection bias that cannot be fully accounted for in this retrospective cohort. In addition, sample sizes were insufficient for meaningful subgroup analyses.
Addition /Modifications:

- We newly included a paragraph in the Methods section:
  "Radiotherapy was defined as adjuvant treatment administered prior to documented progression following index surgery, based on available clinical documentation. Owing to the retrospective multicenter design, systematic early postoperative MRI data for independent verification of EOR, as well as detailed information on radiotherapy timing (e.g., fixed postoperative intervals) and salvage treatment, were not consistently available."
- We restructured the results part to highlight the impact of clinical variables (EOR and RT) and introduced included a new chapter under the subheadline:
  "*Surgical extent as primary prognostic factor within an age-adapted stratification framework*"
  including the following paragraph:
  "Besides extent of resection, radiotherapy is an important prognostic factor; however, detailed information on its timing and intent was inconsistently available (Supplementary Table 7). Patients receiving adjuvant radiotherapy more frequently had unfavorable tumor characteristics, including higher WHO grade and Simpson grade, indicating confounding by indication. After adjustment for these factors and diagnosis timepoint, adjuvant radiotherapy remained associated with a non-significant increase in risk in both multivariable and propensity score–weighted analyses (SI Fig. 4A). This likely reflects residual confounding and selection bias rather than a causal effect."
- We adapted the discussion and included the following paragraphs:
  "The role of adjuvant radiotherapy in this retrospective cohort is more difficult to disentangle because treatment decisions are inherently influenced by tumor grade, residual disease, and clinical judgment, introducing potential confounding by indication." [...]
  "In addition, the retrospective design and variability in clinical management - including surgical approach and use of radiotherapy - introduce potential confounding that cannot be fully addressed without prospective validation, particularly given incomplete data on radiotherapy timing and intent and the limited event numbers available for multivariable modeling. Independent cohorts and prospective studies will therefore be important to confirm the prognostic relevance of the molecular patterns identified here."
- We newly introduced SI Table 7, indicating the patients with available information on EOR based on Simpson Grading, radiotherapy and WHO grade

SI Table 7. Information on available radiotherapy data in the age groups 0-21 years and 22-39 years. EOR = extend of resection according to Simpson grading. Adj. = adjuvant, RT = radiotherapy.

	0-21 years	0-21 years	22-39 years	22-39 years
	No (n=26)	Adj. RT (n=16)	No (n=30)	Adj. RT (n=11)
EOR				
Grade1-3	19 (73.1%)	6 (40.0%)	28 (93.3%)	4 (36.4%)
Grade4-5	7 (26.9%)	9 (60.0%)	2 (6.7%)	7 (63.6%)
Missing	0	1	0	0
WHO grade				
1	15 (57.7%)	5 (31.3%)	21 (70.0%)	3 (27.3%)
2	9 (34.6%)	9 (56.3%)	9 (30.0%)	7 (63.6%)
3	2 (7.7%)	2 (12.5%)	0	1 (9.1%)

- We included a figure for the Propensity score analysis in 0-21-year-old patients with missing value imputation and diagnosis time point as confounder in SI Figure 4 A

A

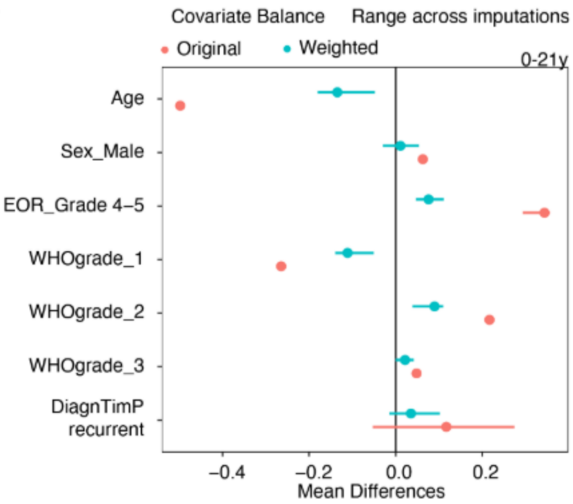

SI Figure 4. (A) Propensity score (PS) analysis in patients 0-21years and the impact of radiotherapy with missing value imputation and diagnosis time point as confounder. EOR = extend of resection based on Simpson grading. DiagnTimP = Timepoint of Diagnosis. [...]

The survival modeling framework appears incomplete for causal/prognostic statements. The current analysis relies predominantly on univariable associations (Kaplan–Meier/log-rank and univariable Cox). If the manuscript aims to state that molecular features are prognostic markers, it should demonstrate independence from established clinical determinants (at minimum: WHO grade, age, EOR, and RT intent) using multivariable Cox, with proportional hazards checks and attention to events-per-variable to avoid overfitting. If multivariable modeling is not feasible due to limited events/missingness, conclusions must be downgraded to exploratory and phrased accordingly.

Response: We thank the reviewer for this important comment. In the revised manuscript, we strengthened the survival analysis by applying a multivariable Cox proportional hazards model to estimate the effect of the proposed risk stratification in patients 0-21y after adjusting for EOR, WHO grade and sex. A Cox model with Firth correction was used to reduce potential bias due to the low events-per-variable ratio (EPV). With 5 parameters to be estimated and 39 events, the event per variable (EPV) ratio of 7.8 was low but acceptable. Missing data were handled using multiple imputation by chained equations (MICE; 50 imputations). The proportional hazards assumption was verified using scaled Schoenfeld residuals and showed no violations. In this subset, risk stratification (low vs intermediate risk, as no BAP1 cases were present) remained significant after adjustment for sex, WHO grade, and extent of resection.

Addition / Modifications:

- We applied the multivariable model and updated the results section (subheadline: “Integrated, hierarchical risk stratification reveals distinct prognostic subgroups in pediatric and AYA meningiomas”
  “When applying the risk stratification displayed in Figure 5A to the presented cohort of 0-21 years of age patients, only low (n = 48) and intermediate (n = 48) cases can be compared since there are no BAP1 altered cases in this group. In a multivariable Cox PH model including risk stratification, extent of resection, WHO grade and sex, only intermediate risk (HR: 2.5, 95%CI 1.2-5.2, p = 0.02) and extent of resection (Simpson grade 4-5, HR: 2.9, 95%CI 1.3-6.1, p = 0.001) remained significant risk factors (SI Table 8).”
- We updated the methods section (subheadline: statistics):

"For the newly established risk model (Fig 5A) for patients 0-21 years of age, a multivariable Cox PH model with Firth correction was fitted to address the potential bias due to the low events per variable ratio. With 5 parameters to be estimated and 39 events, the event per variable (EPV) ratio of 7.8 was low but acceptable. Missing value imputation was done using MICE[46], based on 50 imputations runs. Proportional hazard assumptions were verified based on the scaled Schoenfeld residual approach[47]. The c-index was used to assess prognostic discrimination of different risk stratifications. Propensity score weighted Cox regression based on multiply imputed data sets was performed using R packages MatchThem, cobalt and Weightit. The propensity score weights were estimated with a logistic regression model using age, sex, EOR, WHO grade and diagnosis timepoint as predictors."

- We updated Figure 4

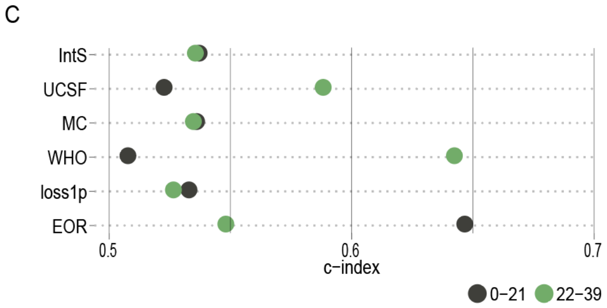

Figure 4 [...] (C) Comparison of the c-index among the patient groups 0-21-years-ols and 22-39-years-old across different stratification systems. IntS = Integrated meningioma risk score, UCSF = UCSF methylation classes, MC = Heidelberg methylation classes, WHO = WHO grades, loss1p = loss of chromosome 1p, EOR = Extend of resection based on Simpson Grading. [...]

- We updated Figure 5 C

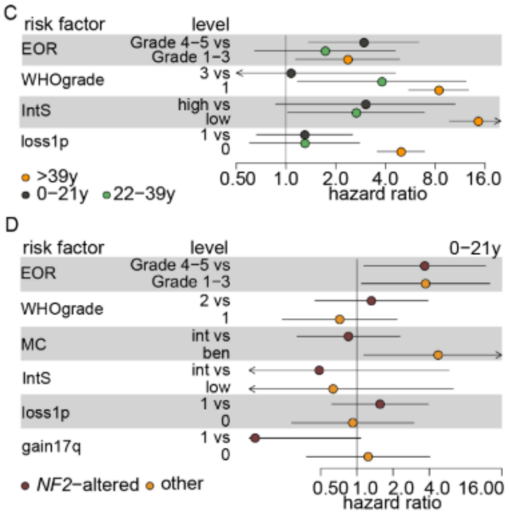

Figure 5 [...] (C) (C) Summary of risk factors and their corresponding hazard ratios for the age groups 0-21 years, 22-39 years and >39 years. EOR = extend of resection based on Simpson Grading, IntS = Integrated meningioma risk score by Maas et al. (2021), y = years. (D) Summary of risk factors and their corresponding hazard ratios in NF2 altered meningiomas and "other" meningioma (0-21 years). The "other subgroup also contains meningiomas with unknown *BAP1* status. EOR = extend of resection based on Simpson Grading, MC = methylation class, ben = benign, int = intermediate, mal = malignant, IntS = Integrated meningioma risk score, y = years.

- We updated SI Figure 4

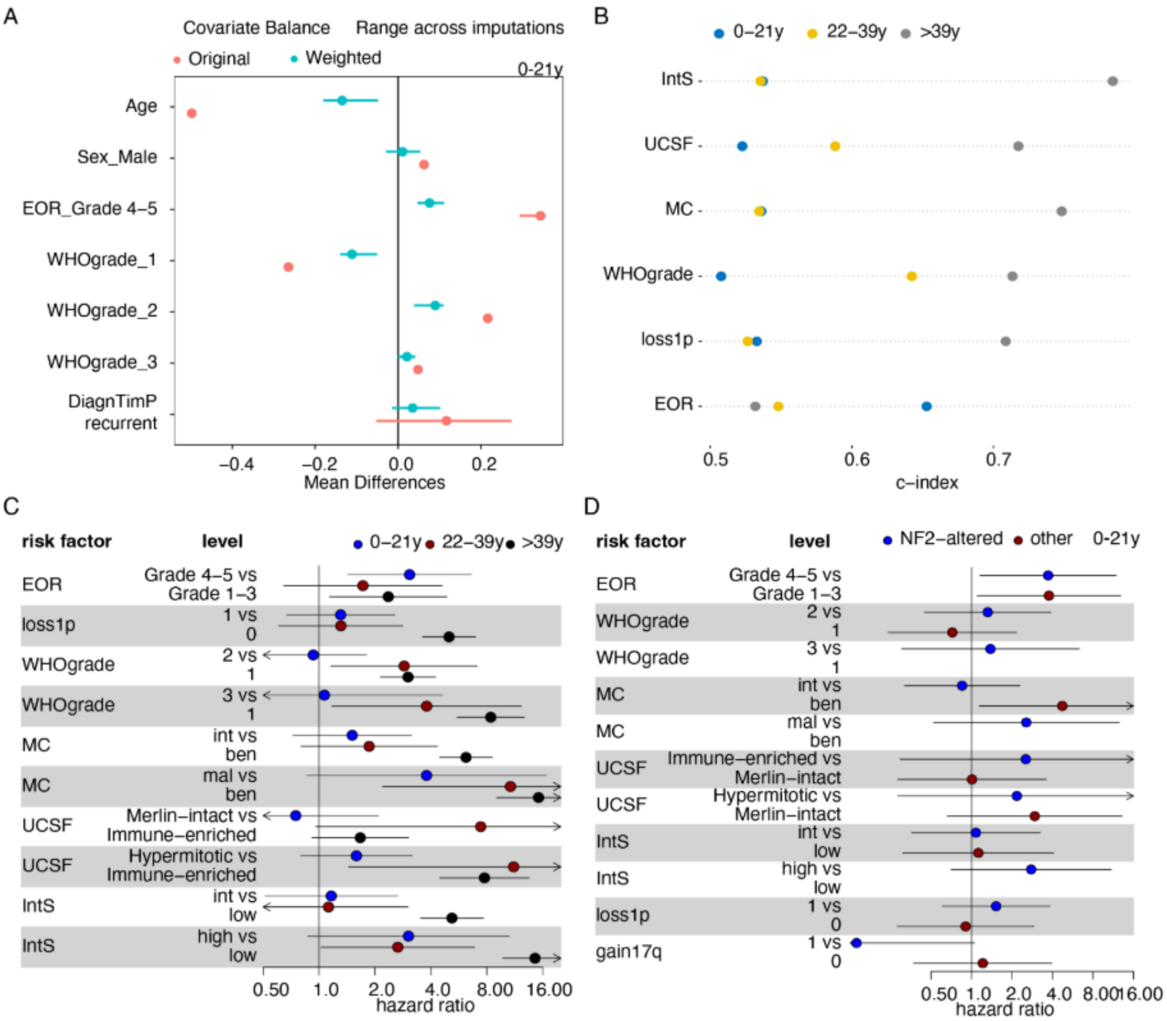

SI Figure 4. (A) ‘love’ plot showing the covariate balance between patients receiving/not receiving radiotherapy before and after propensity score (PS) weighting across imputed data sets in patients 0-21years. EOR = extend of resection based on Simpson grading. DiagnTimP = Timepoint of Diagnosis. (B) Comparison of the c-index among the patient groups 0-21-years-ols and 22-39-years-old and patients > 39 years of age across different stratification systems. IntS = Integrated meningioma risk score, UCSF = UCSF methylation classes, MC = Heidelberg methylation classes, WHO = WHO grades, loss1p = loss of chromosome 1p, EOR = Extend of resection based on Simpson Grading. (C) Summary of risk factors and their corresponding hazard ratios between patients 0-21, 22-39 and >39 years of age. GTR = gross total resection, STR = subtotal resection, MC = methylation class, ben = benign, int = intermediate, mal = malignant, CNA = copy number alterations, IntS = Integrated meningioma risk score. (D) Summary of risk factors and their corresponding hazard ratios between meningiomas from patients 0-21 years of age with *NF2* alterations and the “other” subgroup, including or excluding cases presenting solely a loss of chromosome 22q. EOR = extend of resection, MC = methylation class, int = intermediate, mal = malignant, CNA = copy number alterations, IntS = Integrated meningioma risk score.

Over-strong language / focus imbalance.
The discussion currently foregrounds molecular items as prognostic markers while the evaluation of classical clinical predictors (EOR, RT) is comparatively weak. Given the clinical reality that EOR (and treatment selection for RT) often dominate progression risk, the manuscript should either (a) strengthen and transparently define/adjust these clinical variables, or (b) explicitly acknowledge that molecular findings are hypothesis-generating and potentially confounded by treatment and surgical factors.

<u>Response:</u> We thank the reviewer for this important comment regarding the balance between molecular and clinical factors. We agree that classical clinical determinants, particularly extent

of resection and treatment-related variables, are key drivers of outcome in this setting. In the revised manuscript, we addressed this in two ways. First, we strengthened the analytical framework by performing multivariable Cox regression analyses adjusting for established clinical confounders, where data availability permitted, to evaluate molecular features in the context of known clinical predictors. Second, we revised the Discussion to more explicitly emphasize the primacy of clinical factors, particularly extent of resection, and to highlight potential confounding by indication for radiotherapy. We now clearly state that molecular associations should be interpreted cautiously and are best considered in the context of these clinical determinants.

<u>Addition / Modifications:</u>

- We adapted the Discussion and included the following statements: [...]
  "At the same time, our analyses confirm that classical clinical determinants – most notably extent of resection in tumors arising ≤21 years – remain central to patient outcome, underscoring the importance of interpreting molecular findings in conjunction with surgical management." [...]
  "However, within the pediatric-associated subgroup (0-21 years), extent of resection remained the strongest predictor of progression across molecular categories. This observation emphasizes that classical clinical factors, particularly extent of resection, remain the dominant determinants of outcome in this cohort and must be considered when interpreting molecular associations." [...]
  "The role of adjuvant radiotherapy in this retrospective cohort is more difficult to disentangle because treatment decisions are inherently influenced by tumor grade, residual disease, and clinical judgment, introducing potential confounding by indication." [...]
  "Together, these observations support a hierarchical interpretation of molecular features in which driver alterations define primary biological groups and additional genomic features refine risk within those contexts." [...]
  "Although the integration of multiple molecular layers supports the robustness of these observations, outcome data and germline information were not uniformly available across the cohort, limiting statistical power in some subgroup analyses. In addition, the retrospective design and variability in clinical management - including surgical approach and use of radiotherapy - introduce potential confounding that cannot be fully addressed without prospective validation, particularly given incomplete data on radiotherapy timing and intent and the limited event numbers available for multivariable modeling. Independent cohorts and prospective studies will therefore be important to confirm the prognostic relevance of the molecular patterns identified here."

Reviewer #2

This is a high-quality, conceptually impactful study that significantly advances understanding of pediatric and AYA meningioma biology. The depth of multi-omic integration and the consistency of the findings convincingly establish young-onset meningiomas as a biologically distinct disease entity requiring age-adapted risk stratification. The work has clear implications for future WHO classifications, diagnostic frameworks, and clinical management, and constitutes a substantial contribution to the field. The minor limitations inherent to retrospective, multi-institutional studies and small numbers in rare subgroups. Overall, this manuscript represents a significant contribution to the field and is highly suitable for publication.

Manuscript Strengths

1. The study represents one of the largest and best-curated cohorts of pediatric and AYA meningiomas, providing robust and generalizable conclusions.
2. The manuscript convincingly shows that adult prognostic frameworks (WHO grade, methylation class, integrated risk scores, chr1p loss) fail in patients ≤21 years, addressing a major unmet need.
3. Integration of histopathology, methylation profiling, CNV analysis, and sequencing reveals a distinct, gain-dominated CNV landscape (notably chr17q gain) and enables clinically meaningful, driver-based subgrouping.
4. The consistent prognostic importance of extent of resection across molecular subgroups and the proposed age-adapted risk stratification model provide clear, practice-relevant guidance.

Response: We would like to thank the reviewer for the positive evaluation of our manuscript. We greatly appreciate the recognition of the study's strengths and its relevance for improving age-adapted risk stratification in meningioma.

Minor Limitations

1. Progression-free survival and germline status are available only for subsets of patients, limiting power in some subgroup analyses.

Response**:** We thank the reviewer for this important comment. We agree that progression-free survival and germline status are only available for subsets of patients, which limits statistical power in some subgroup analyses. We have addressed and further clarified these limitations throughout the manuscript where applicable (see also responses to Reviewer #1 and #3 Comments) and have discussed these constraints in the Discussion.

Addition / Modification:

- We included this limitation in the discussion:
  "Outcome data and germline information were not uniformly available across the entire cohort, limiting statistical power in some subgroup analyses."

2. Low case numbers for BAP1-altered and YAP1-fusion tumors restrict definitive prognostic conclusions.

Response**:** We thank the reviewer for this important comment. We agree that the low number of BAP1-altered and YAP1-fusion tumors limits definitive prognostic conclusions for these subgroups. This limitation has been explicitly added to the Discussion to ensure appropriate interpretation of these findings.

Addition / Modification:

- We included this limitation in the discussion:
  "Further, low case numbers for BAP1-altered and YAP1-fusion tumors restrict definitive prognostic conclusions."

3. While adult methylation classes show limited utility, a fully validated pediatric-specific classifier is not yet proposed.

Response**:** We thank the reviewer for this insightful comment. We agree that, while adult methylation-based classifiers show limited utility in this cohort, our data do not yet support a fully established pediatric-specific classification and prognostication system. Similar to other pediatric CNS tumor entities, young-onset meningiomas appear biologically heterogeneous, and a robust dedicated classifier will require further prospective validation in larger cohorts. Nevertheless, we believe our study provides strong evidence that adult-based frameworks are insufficient in this age group and should not be applied in this setting. Importantly, our findings also support feasible and biologically meaningful molecular stratification strategies that may form the basis for future age-adapted classification systems.

Reviewer #3

This study provides a valuable large-scale integrated characterization of youth-onset meningiomas. However, key conclusions regarding the biological distinctness of tumors ≤21 years and the proposed hierarchical risk model require more consistent age stratification, additional analyses, and stronger statistical support before acceptance. I suggest major revision. Here are my major and minor concerns:

Response: We sincerely thank Reviewer #3 for the careful evaluation of our manuscript and for the detailed and constructive comments. We particularly appreciate the reviewer's insights regarding age stratification, the characterization of the 22-39-year cohort, and the statistical support for our conclusions. We have revised the manuscript accordingly to address these points, and we provide detailed, point-by-point responses below.

Major Comments:

1. Inconsistent and shifting age stratification

While the manuscript initially defines two stratification approaches (0–14 vs 15–39 and ≤21 vs >21), the analyses and figures inconsistently switch between these groupings, making it difficult to follow and weakening the claim that ≤21 years represents a distinct biological entity:

• Figure 1B–D: analysis restricted to 0–21 years
• Figure 1E: entire 0–39 cohort
• Figure 1F: restricted to 0–14 years
• Figure 5: conclusions based primarily on 0–21 years
• Figure 5B: age group definition not clearly stated

If the central conclusion is that tumors arising ≤21 years constitute a biologically distinct entity, then:

• Key CNV and survival analyses should be consistently shown for the 0–21 group.
• Figure 1F CNV landscape should be shown for 0–21, not only 0–14.
• and possibly for 21-39 to see/show that they are "more similar" to adult cohort.
• Figure 1E (chr1p survival) should be repeated specifically for 0–21.
• The 21–39 group must be analyzed directly rather than inferred.

The 0-21 age threshold appears data-driven but insufficiently justified analytically (specifically for 21-39 samples).

Response: We thank the reviewer for this important comment and agree that consistent age stratification is critical for the interpretation of our findings. In response, we have revised the manuscript to apply a uniform analytical framework, with all key molecular and survival analyses now consistently presented for the 0-21-year cohort in the main figures.

In addition, we have directly analyzed the 22–39-year cohort, as suggested. In this group, established adult prognostic markers - including WHO grade, methylation class, and UCSF classification – provide prognostic stratification. However, the hazard ratios and the c-index are lower than in the patient group aged 39 and older, supporting the concept of a gradual transition toward adult-type biology. To maintain clarity of presentation, the main figures focus on the ≤21-year cohort, while analyses of the 22–39-year group are included where relevant and otherwise provided in the Supplementary Information, with explicit reference in the main text.

With regard to the ≤21-year cutoff, we agree that this threshold should not be interpreted as a strict biological boundary. Due to limited event numbers in intermediate age ranges, particularly between approximately 18 and 25 years, the available data does not support a robust data-driven determination of an optimal cutoff. Exploratory analyses across different age thresholds showed no clear superiority of alternative stratifications, and smaller cutoffs (e.g., 14–16 years) were not supported by the data. We have therefore retained the 0-21-year definition as a pragmatic and biologically informed grouping that captures the predominant pediatric-associated molecular profile while allowing comparison to older patients. Notably, the

principal molecular features defining the ≤21 group were consistently observed across this age range, supporting the biological coherence of this grouping despite the absence of a sharp threshold. We have addressed this in the discussion.

<u>Addition / Modifications:</u>

- We revised the complete introduction, results and discussion part to focus on the age groups 0-21 years and 22-39 years of age. The manuscript no longer discusses or presents any analyses for the PED and AYA stratification approach.
- We included a paragraph in the discussion to address the age-cut-off:
  "Due to the low incidence of meningiomas up to 39 years of age, it is challenging to define in a truly data-driven manner at which point the underlying biological or clinical systems reliably apply and when they cease to do so. This issue is further enhanced given the slow growth and long latency typical of meningiomas. This represents also a limitation of our study but at the same time, there is a lack of large multicenter datasets that would adequately address this question.
  Here, we introduce a model that demonstrates improved performance compared to previously applied approaches from adults. However, despite these advances, a degree of uncertainty remains, particularly for patients in their mid-twenties, regarding accurate classification and clinical interpretation."
- Figure 1 now shows the survival analysis of the 0-21 years of age group when stratifying for: WHO, methylation class and UCSF class (panel B-D), the loss of chr1p (panel E) and the copy number analysis (panel F)

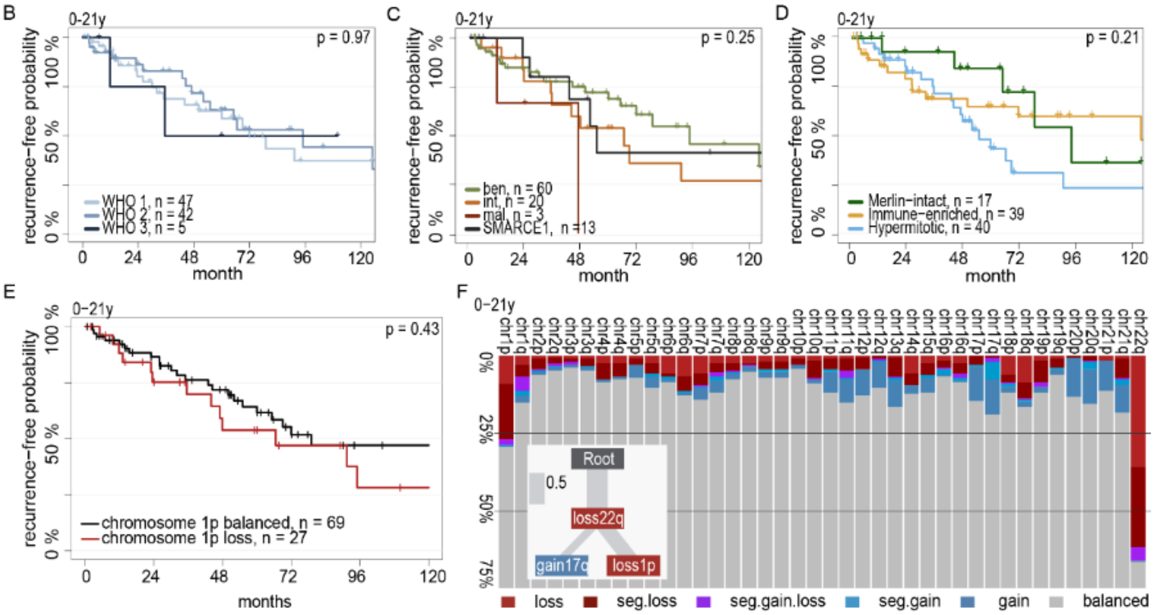

Figure 1. [...] (B) Kaplan-Meier analysis of risk of progression of the subgroup of patients 0–21 years, stratified according to WHO grade (C) methylation class (0-21 years) (D) and UCSF class (0-21 years). The p-values were calculated using the Log-rank test. (E) Kaplan-Meier analysis of risk of progression for patient 0-21 years of age, stratified according to the presence or absence of a chromosome 1p loss. The p-value was calculated using the Log-rank test. y = years (F) Overview of chromosomal alterations in the 0-21 years group, (n = 212), plotted across individual chromosomal arms. For the oncogenetic trees, only gains/losses with at least 10% prevalence within the respective age cohort were used and only the first two nodes are shown. seg. = segmental, chr = chromosome, y = years.

- Figure 2 C (deconvolution results) was updated to show the results for the age groups: 0-21 years, 22-39 years and >39 years

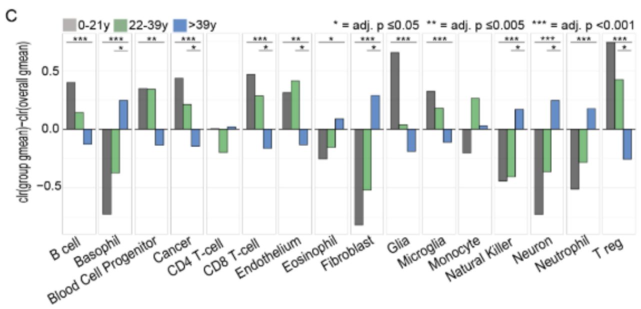

Figure 2. [...] (C) Compositional data analysis plot based on bulk methylation deconvolution data for meningiomas from patients 0-21 years, 22-39 years and adults (>39 years). clr = centered log ratio. P-values were retrieved from SI Table 2. [...]

- Figure 4 was updated to include a c-index comparison between the patient groups 0-21 years and 22-39 years of age (panel C) and a Kaplan-Meier plot for the age group 22-39 years stratified for WHO grade (highest c-index) (panel D)

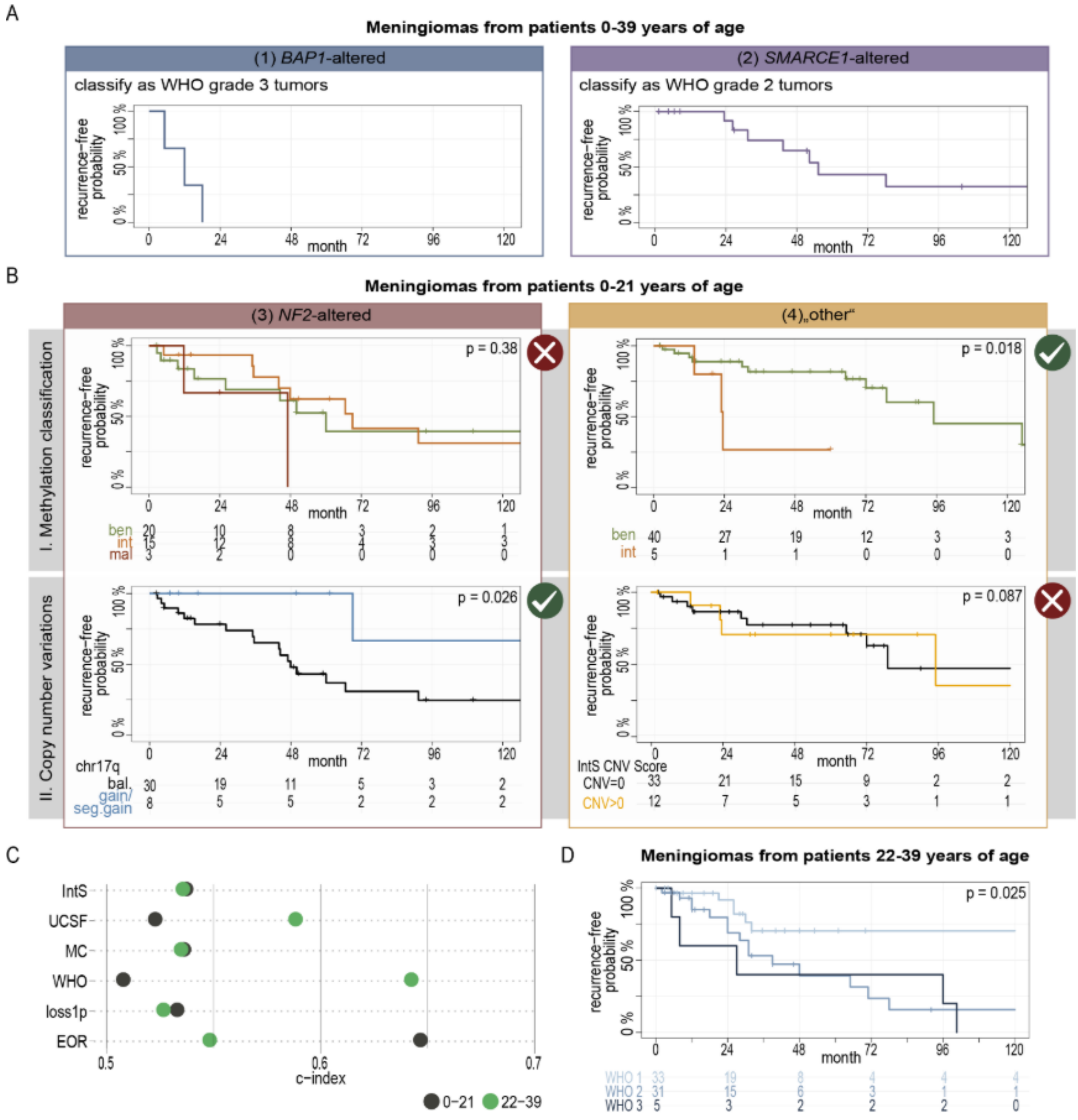

Figure 4. (A) (1) *BAP1*-altered meningiomas show a highly aggressive clinical behavior and should be classified as WHO grade 3 (Sievers et al. (2025)). The Kaplan–Meier analysis included all cases from 0–39 years. (2) *SMARCE1*-altered meningiomas, representing a distinct molecular entity and should be classified as WHO grade 2 (Sievers et al (2021)). The Kaplan–Meier analysis included patients from 0–39 years. (B) Patient group 0-21 years of age: (3) *NF2* altered meningiomas; and (4) tumors not falling into these categories, classified as "other". The "other subgroup also contains meningiomas with unknown *BAP1* status. The Kaplan–Meier analyses of the progression risk for the *NF2*-altered and "other" subgroups of patients 0-21 years of age, stratified by I. methylation class, ben = benign, int = intermediate, mal = malignant, II. by copy-number alterations: chromosome 17q gain for the *NF2* altered subgroup and the presence of high-risk copy number variations as defined by the Integrated Risk Score for meningiomas for the "other" subgroup. chr = chromosome, bal. = balanced, seg.gain = segmental gain, IntS = Integrated risk score, CNV = copy number variation. The p-values were calculated using the Log-rank test. (C) Comparison of the c-index among the patient groups 0-21-years-old and 22-39-years-old across different stratification systems. IntS = Integrated meningioma risk score by Maas et al. (2021), UCSF = UCSF methylation classes by Choudhury et al. (2022), MC = Heidelberg methylation classes by Sill et al. (2026), WHO = WHO grades, loss1p = loss of chromosome 1p, EOR = Extend of resection based on Simpson Grading. (D) Kaplan-Meier analysis of risk of progression of the subgroup of patients 22–39 years, stratified according to WHO grade. The p-values were calculated using the Log-rank test.

- Figure 5 A was revised to also include the patient group 22-39 years of age, panel B includes the hazard ratios for both age groups and panel C shows how hazard ratios for the driver groups in the 0-21 years of age cohort

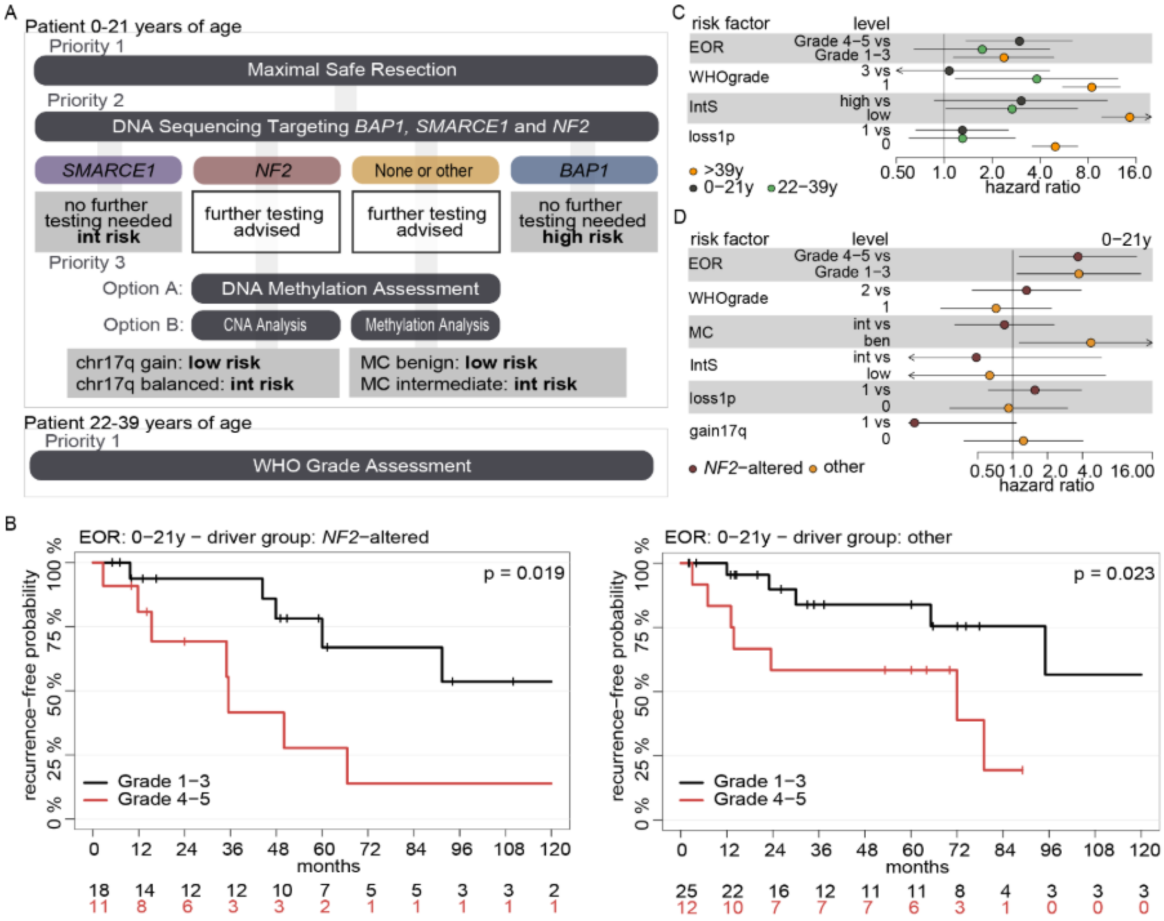

Figure 5. (A) Workflow for efficient meningioma evaluation in patients aged 0–21 years in diagnostic routine. Maximal safe resection remains the highest priority for reducing recurrence risk, and detailed documentation of the extent of resection is essential for accurately assessing and defining the individual risk of recurrence. As a second priority, DNA panel sequencing should be performed to detect alterations in *SMARCE1, BAP1* and *NF2*. Meningiomas harboring *SMARCE1* or *BAP1* alterations do not require additional molecular testing and can be stratified as intermediate-risk and high-risk for recurrence, respectively. For *NF2*-altered meningiomas and meningiomas without alterations in *SMARCE1, BAP1* or *NF2*, further molecular workup can follow two alternative paths: Option A (feasible for both subgroups): Methylation data can be generated using, for example, nanopore sequencing or methylation array profiling, allowing the simultaneous assessment of copy-number variation and methylation class. Option B (subgroup-specific): For *NF2*-altered cases, targeted analysis

of chromosome 17q copy-number status (e.g., via FISH) can be performed, while for *NF2*-wild-type / other cases, methylation profiling alone is sufficient to enable subsequent stratification into low- and intermediate-risk groups. CNA: copy number alteration, in = intermediate, MC = methylation class, chr = chromosome. For patients aged 22–39 years, the WHO graduation has the highest priority. (B) Kaplan–Meier analysis for the patient group 0-21 years of age for the driver groups "*NF2*-altered" and "other", stratified for extent of resection Simpson grade 1-3 and 4-5. EOR = extend of resection, y = years, The p-values were calculated using the Log-rank test. (C) Summary of risk factors and their corresponding hazard ratios for the age groups 0-21 years, 22-39 years and >39 years. EOR = extend of resection based on Simpson Grading, IntS = Integrated meningioma risk score by Maas et al. (2021), y = years. (D) Summary of risk factors and their corresponding hazard ratios in *NF2* altered meningiomas and "other" meningioma (0-21 years). The "other subgroup also contains meningiomas with unknown *BAP1* status. EOR = extend of resection based on Simpson Grading, MC = methylation class, ben = benign, int = intermediate, mal = malignant, IntS = Integrated meningioma risk score, y = years.

- SI Figure 1 now include survival analysis of the 22-39 years of age group when stratifying for: WHO, methylation class and UCSF class (panel B), loss of chromosome 1p (panel C) and the copy number plot (panel E) and for both age groups: the survival analysis when stratifying for the integrated risk score (panel D)

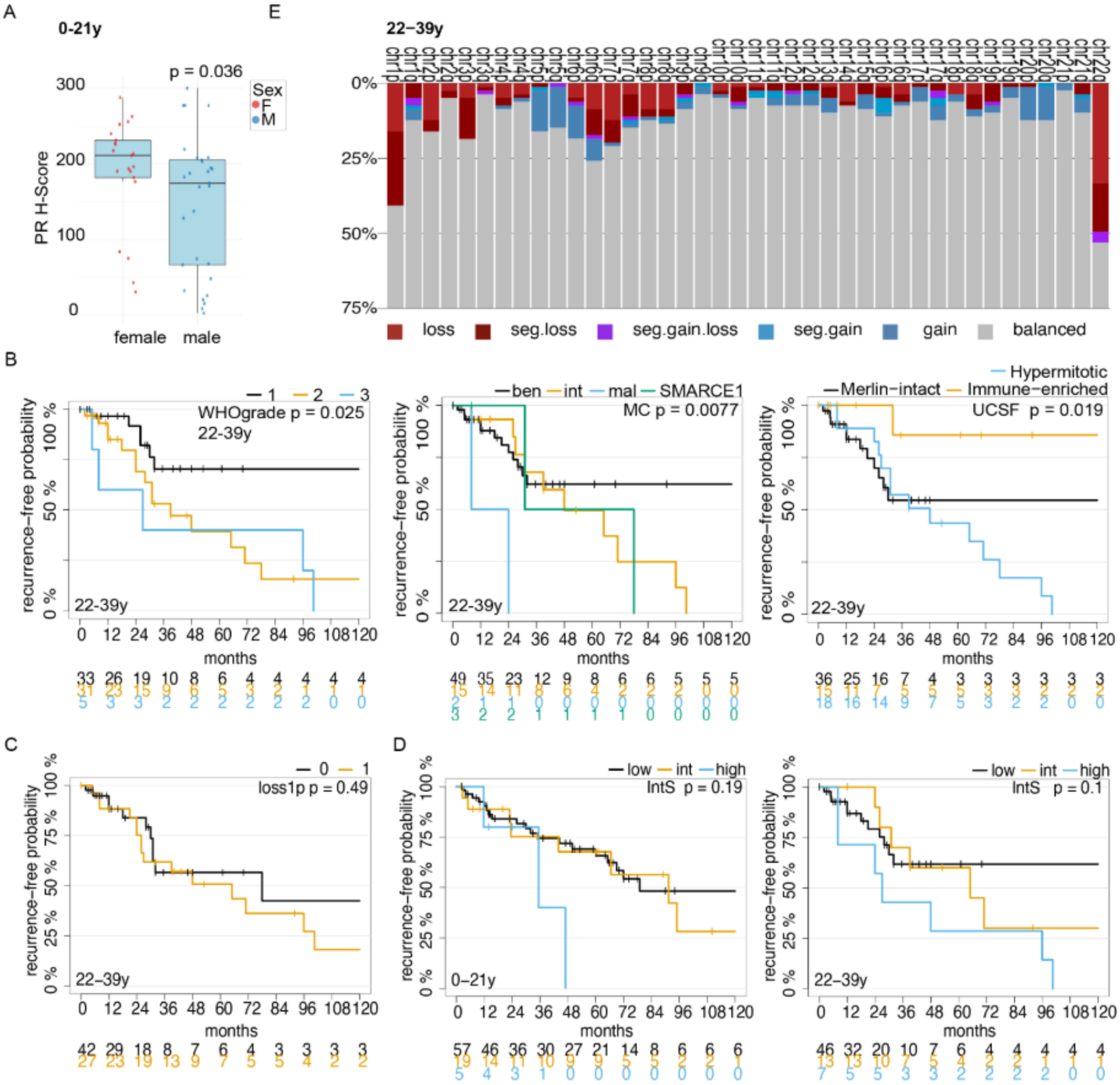

SI Figure 1. (A) Boxplot comparing progesterone receptor staining H-scores between meningiomas from female and male patients 0-21 years of age. The p-values were calculated using the Wilcoxon rank sum test. PR = progesterone receptor, F = female, M = male, y = years. (B) Kaplan-Meier analysis of risk of progression of the subgroup of patients 22-39 years of age stratified according to WHO grade, methylation class and UCSF class. The p-values were calculated using the Log-rank test. MC = methylation class, Ben = benign, int = intermediate, mal = malignant, y = years (D) Kaplan-Meier analysis of risk of progression of the subgroup of patients 22-39

years of age, stratified according to the presence or absence of a loss of chromosome 1p. The p-values were calculated using the Log-rank test. y = years (D) Kaplan-Meier analysis of risk of progression of the subgroup of patients 0-21 and 22-39 years of age, stratified according to integrated risk score. The p-values were calculated using the Log-rank test. (E) Overview of chromosomal alterations in meningiomas from 22-39-year-old patients. seg. = segmental, chr = chromosome, y = years.

- SI Figure 2 now includes the oncogenetic tree for the patient groups: 0-21 years, 22-39 years and >39 years.

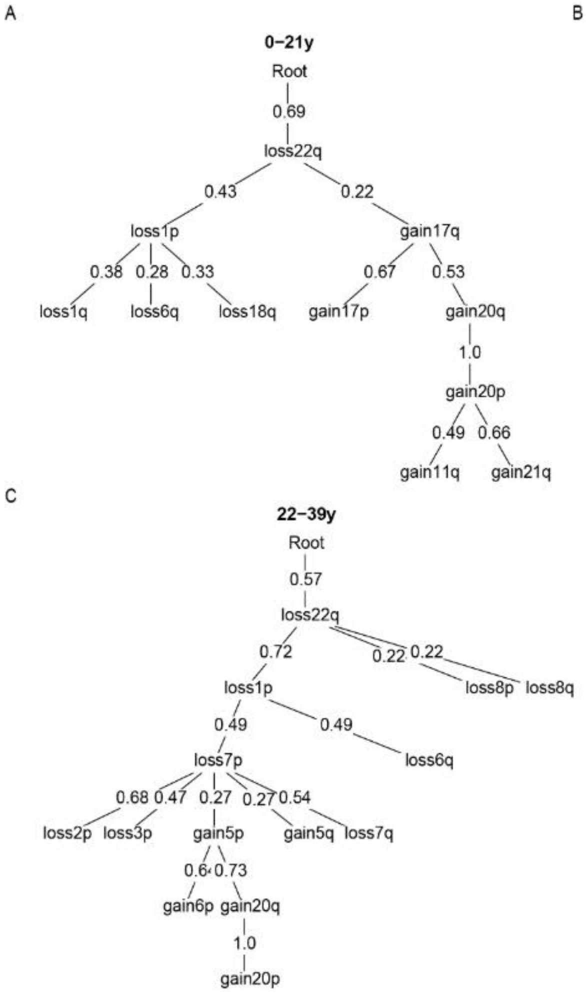

SI Figure 2. Oncogenetic trees for meningiomas in the age groups (A) 0-21 years, (B) 22-39 years and (D) >39 years. Only gains/losses with at least 10% prevalence within the respective age cohort were used. y = years

- SI Figure 3 C now included the survival analysis for the age group 22-39 years when stratifying for the microglia share

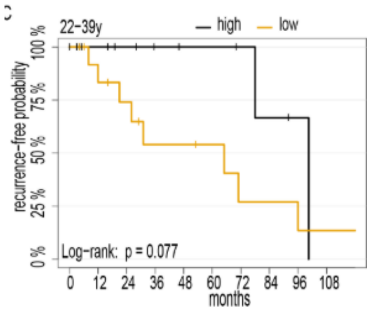

SI Figure 3 [...] (C) Kaplan-Meier analysis of risk of progression of the subgroup of patients 22–39 years stratified for the top and bottom 20% of microglia share based on the bulk methylation deconvolution data. The p-values were calculated using the Log-rank test. [...]

- SI Table 1, 2, 3 and 7 include data for the 0-21 and 22-39-year-old patient groups

Additionally, the title: “Integrated, hierarchical risk stratification reveals distinct prognostic subgroups in pediatric and AYA meningiomas” is inconsistent with the fact that the stratification model applies only to ≤21 and AYA refers to 15-39.
The authors should clarify whether the model applies to:
• PED only?
• PED + AYA?
• ≤21 only?
Response and Modification: We thank the reviewer for this comment and have adjusted the title accordingly to reflect the revised manuscript:
“Distinct molecular subgroups in pediatric and young-onset meningiomas require age-adapted risk stratification”

2. The 21–39 age group is not characterized; it is not clear how they conclude that >21 converges to adult cohort. The Discussion proposes that tumors in the 21–39 age group progressively resemble adult disease. However:
• There is no focused molecular or survival analysis specifically on 21–39.
• Figure 4 and the hierarchical model apply only to ≤21.
• It remains unclear whether adult prognostic frameworks perform adequately in 21–39.
Response: We thank the reviewer for this important comment and agree that the 22–39-year age group required clearer characterization to support the interpretation that this subgroup shows increasing similarity to adult meningioma. In response, we have revised the manuscript to more clearly distinguish between the ≤21-year and 22–39-year cohorts. These additional analyses show that, in the 22–39-year group, established adult-associated markers, including WHO grade, methylation class, and UCSF classification, retain stratification capacity, in contrast to the ≤21-year cohort. This supports the interpretation that tumors in patients aged 22–39 years show a transition toward adult-type biology. Furthermore, we have expanded the figures related to the proposed diagnostic/prognostic framework to include, for completeness, on which framework tumors in the 22–39-year group should be diagnosed. While the hierarchical model remains primarily derived from the ≤21-year cohort, the revised figures and text now make clear how the 22–39-year group relates to this framework and where adult prognostic systems appear to become informative.
Addition / Modifications:
We would like to point out that we have already listed the relevant changes in Comment 1 and included the figures/tables, so we will not include them again.

- We revised the complete results and discussion part to focus on the age groups 0-21 years and 22-39 years of age. The manuscript no longer discusses or presents any analyses for the PED and AYA stratification approach.
- Figure 1 now shows the survival analysis of the 0-21 years of age group when stratifying for: WHO, methylation class and UCSF class (Panel B-D), the loss of chromosome 1p (panel E) and the copy number analysis (F)
- SI Figure 1 no include survival analysis of the 22-39 years of age group when stratifying for: WHO, methylation class and UCSF class (Panel B), loss of chromosome 1p (C) and the copy number plot (E) and for both age groups: the survival analysis when stratifying for the integrated risk score (D)
- SI Figure 2 now includes the oncogenetic tree for the patient groups: 0-21 years, 22-39 years and >39 years.
- Figure 2 C (deconvolution results) was updated to show the results for the age groups: 0-21 years, 22-39 years and >39 years

- SI Figure 3C now included the survival analysis for the age group 22-39 years when stratifying for the microglia share
- Figure 4 was updated to include a c-index comparison between the patient groups 0-21 years and 22-39 years of age (panel C) and a Kaplan-Meier plot for the age group 22-39 years stratified for WHO grade (highest c-index) (panel D)
- Figure 5 A was revised to also include a priority for the patient group 22-39 years of age, panel B includes the hazard ratios for both age groups and panel C shows hazard ratios for the driver groups in the 0-21 years of age group
- SI Table 1 now includes data for 0-21 as well as the 22-39-year-old patient groups

3. Germline status requires more rigorous integration
Germline status of this cohort is a very important factor and should be clarified/addressed earlier, and many (if not all) of the analysis should be performed based on germline status, either by excluding the cases with known predisposing germline variants, or by correcting for it. Here are a few related questions/concerns:
• Why are only germline NF2 mutations considered?
• Should analyses be repeated excluding known predisposition cases?
• Do germline NF2 tumors cluster together in methylation space?

Response: We thank the reviewer for emphasizing the importance of germline predisposition in our cohort of early onset meningiomas. We fully agree that germline status represents a critical biological variable in this disease context. However, systematic germline assessment was not available across this retrospective, multi-institutional cohort, limiting our ability to uniformly incorporate germline status into all analyses. Among cases with available clinical annotation, *NF2* represented the only recurrent and a bit more consistently documented germline alteration, whereas other predisposition syndromes (e.g., *SMARCE1, BAP1*) were not systematically assessed or were available only in very small numbers.
Regarding DNA methylation-based clustering, previous studies have demonstrated that *BAP1- and SMARCE1*-driven meningiomas form highly distinct DNA methylation classes that are largely independent of whether the underlying alterations are of germline or somatic origin (PMID: 40249111, PMID: 33319313). In contrast, *NF2*-altered meningiomas do not appear to segregate in a similarly distinct manner. To address this point within our cohort, we have provided an overview of *NF2*-altered cases in a t-SNE representation, which shows that tumors with known germline *NF2* alterations do not form a separate cluster but are intermixed with non-germline *NF2* cases across methylation space. Importantly, given the limited number of cases with confirmed germline annotation, a systematic stratified analysis of DNA methylation clustering by germline status was not feasible. We therefore interpret these findings cautiously and emphasize that definitive conclusions regarding the contribution of germline status to methylation-based subgrouping cannot be drawn from the present cohort.
We have revised the manuscript to more clearly acknowledge these limitations and have incorporated these points into the Discussion to better contextualize the role of germline status and its current analytical constraints. Additionally, we highlight the need for future studies incorporating systematic germline characterization in uniformly profiled cohorts.
Addition / Modifications:

- We included a paragraph in the Methods section (subheading: “Targeted DNA and RNA sequencing“):
  “Systematic germline testing was not available for this retrospective cohort; therefore, germline status could not be comprehensively assessed across all cases.”
- We newly analyzed if *NF2* germline mutated meningiomas cluster together in a t-SNE projection based on their methylation profile (SI Figure 3 I) and included in the results section (subheading: “Driver alterations shape tumor composition”)

"The NF2-germline mutated tumors also did not cluster together in the t-SNE projection (SI Fig 3I)."

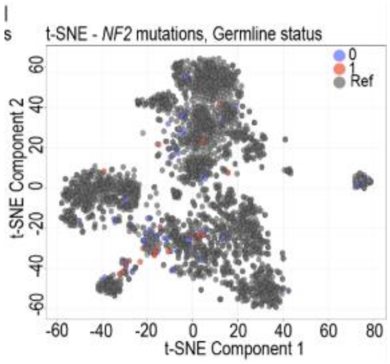

SI Figure 3 [...] (I) Methylation Clustering displayed as a t-SNE projection (20 perplexity, 1000 iterations, distance metric: weighted Pearson) of the PED/AYA cases of this study with 2,197 meningiomas retrieved from the database of the neuropathology department in Heidelberg with available information on age and sex of the patients and a methylation family score for meningioma >0.9. Cases were colored based on information on *NF2* mutation and respective germline status.

- We modified the discussion and included following paragraphs:
  "And, while the lack of systematically available information on germline status represents a limitation of this study, it in fact reflects current clinical reality. " [...]
  "Although our integration of multiple molecular layers supports the robustness of these observations. Outcome data and germline information were not uniformly available across the entire cohort, limiting statistical power in some subgroup analyses."

4. TME deconvolution analysis needs further stratification
The deconvolution analysis (Figure 2C, 3B) shows differences between youth and adult tumors, and between driver-defined groups.
Currently, this analysis seems immature and can be further evaluated.

Response: We thank the reviewer for this important suggestion and have revised the analysis accordingly. To generally improve this analysis, we added a multivariable analysis for estimating the effect of age group and driver mutation/MC on each TME component. EOR is not included because no effect on TME was seen in the subgroup of samples with EOR information and to avoid reduced sample size in multivariable models due to missing information of EOR.
Modification:

- The result section was expanded (subheading: "Patient age influence DNA methylation clustering of meningiomas and cellular composition"):
  "In direct comparison between the 0-21-year-old and 22-39-year-old groups, no significant differences were detected for any of the individual cellular components - across the complete cohorts and even when comparing age groups within the MC (Fig. 2C, SI Table 2, 3). Also, when fitting a multivariable linear model for each TME component and including age and MC or driver status as covariates, there was no significant difference in the comparison between the 0-21 and 22-39-years-of-age groups (SI Fig. 3A)."
- The results section was adapted (subheading: "Driver alterations shape tumor composition"):
  "This also holds true when fitting a multivariable model (SI Fig. 3E) and when comparing adult cases with either an NF2 or SMARCE1 alteration (p < 0.001), indicating rather a driver than age specific effect (SI Table 4 and 5)."

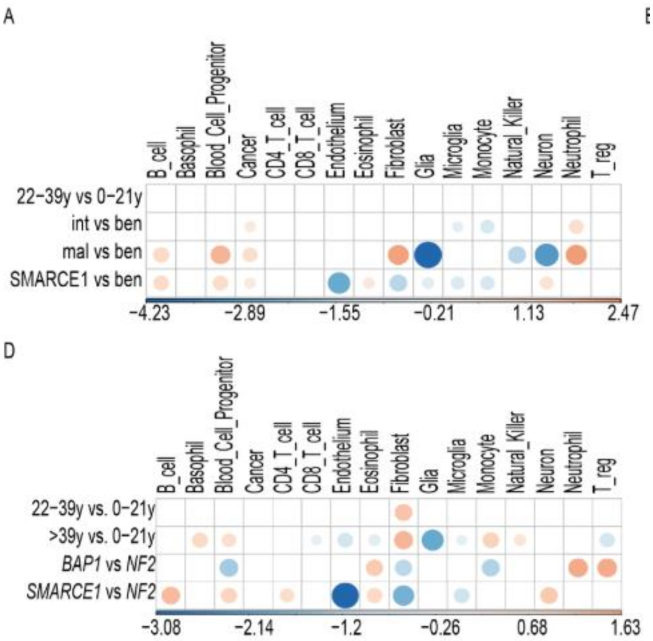

SI Figure 3 (A) multivariable analysis for tumor microenvironment for age cohort and methylation classes, patients 0-39 years: For each individual tumor microenvironment component, a multivariable linear model with the tumor microenvironment component (using PLR1 transformation) as outcome is fitted. I.e., ach column in the plot gives estimates from one multivariable model. Only significant effects are displayed. P-values are adjusted across tumor microenvironment components. Red/blue indicates a relative increase/decrease of a tumor microenvironment component associated with a certain factor. MC = methylation class. [...]
(D) multivariable analysis for tumor microenvironment for age cohort and driver group, all patients excluding driver group other (0-39 years, n = 267): For each individual tumor microenvironment component, a multivariable linear model with the TME component (using PLR1 transformation) as outcome is fitted. I.e., ach column in the plot gives estimates from one multivariable model. Only significant effects are displayed. P-values are adjusted across TME components. Red/blue indicates a relative increase/decrease of a TME component associated with a certain factor. MC = methylation class. [...]

Addressing the reviewer's other valuable comments: First, we repeated the PED vs. AYA comparisons within methylation families to disentangle age-driven from cluster-driven effects. Second, we extended the driver-based TME comparisons. BAP1 was not included in the pediatric comparisons due to limited case numbers. Third, we have annotated adjusted p-values directly in Figures 2C and 3B to improve clarity and transparency of the statistical evaluation. The modifications are listed under the respective points below

Analyses comparing PED vs AYA should be repeated within methylation clusters to determine whether differences are age-driven or cluster-driven.

Modifications:

- The result section was expanded to include the TME comparison between 0-21 and 22-39-year-old patients within methylation classes (subheading: "Patient age influence DNA methylation clustering of meningiomas and cellular composition"):
  "In direct comparison between the 0-21-year-old and 22-39-year-old groups, no significant differences were detected for any of the individual cellular components - across the complete cohorts and even when comparing age groups within the MC (Fig. 2C, SI Table 2, 3). Also, when fitting a multivariable linear model for each TME component and including age and MC or driver status as covariates, there was no significant difference in the comparison between the 0-21 and 22-39-years-of-age groups (SI Fig. 3A)."

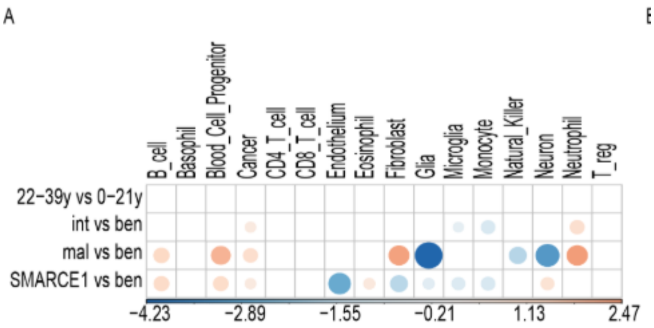

SI Figure 3 (A) multivariable analysis for tumor microenvironment for age cohort and methylation classes, patients 0-39 years: For each individual tumor microenvironment component, a multivariable linear model with the tumor microenvironment component (using PLR1 transformation) as outcome is fitted. I.e., ach column in the plot gives estimates from one multivariable model. Only significant effects are displayed. P-values are adjusted across tumor microenvironment components. Red/blue indicates a relative increase/decrease of a tumor microenvironment component associated with a certain factor. MC = methylation class. [...]

- SI table 3 was included to present the results from the deconvolution data when stratifying for the methylation classes

SI Table 3. Evaluation of deconvolution data with tests on individual logratios between age groups and within methylation classes. Adj.p. = adjusted p value, est = estimate, T reg = T regulator cell

MC	ben	ben	int	int	mal	mal
	0-21y vs 22-39y	0-21y vs 22-39y	0-21y vs 22-39y	0-21y vs 22-39y	0-21y vs 22-39y	0-21y vs 22-39y
	est.	adj.p.	est.	adj.p.	est.	adj.p.
B cell	0.19	0.696	0.30	0.569	-2.63	0.577
Basophil	-0.25	0.696	-0.96	0.569	1.39	0.603
Blood Cell Progenitor	0.24	0.696	-0.69	0.571	-4.00	0.465
Cancer	0.10	0.736	-0.09	0.937	0.58	0.577
CD4 T-cell	0.17	0.736	0.65	0.569	0.53	0.577
CD8 T-cell	0.35	0.696	0.06	0.937	-0.69	0.682
Endothelium	-0.07	0.844	0.17	0.932	0.55	0.577
Eosinophil	-0.28	0.696	-0.01	0.992	1.05	0.465
Fibroblast	0.07	0.861	-0.59	0.569	0.56	0.577
Glia	0.32	0.696	1.55	0.569	3.08	0.465
Microglia	0.31	0.696	0.10	0.937	-0.38	0.682
Monocyte	-0.10	0.844	-1.13	0.569	-1.56	0.577
Natural Killer	-0.23	0.696	0.77	0.569	0.53	0.577
Neuron	-0.70	0.506	-0.57	0.569	2.87	0.465
Neutrophil	-0.46	0.696	0.10	0.937	-0.48	0.682
T reg	0.35	0.696	0.37	0.932	-1.42	0.577

Driver-based TME comparisons (NF2 vs SMARCE1 vs BAP1) should be repeated in >40 patients where possible to determine whether differences are age-specific or driver-specific.

<u>Modifications:</u>

- The result section was expanded to include driver based TME comparisons with patients >39 years of age (subheading “Driver alterations shape tumor composition”):

“This also holds true when fitting a multivariable model (SI Fig. 3E) and when comparing adult cases with either an NF2 or SMARCE1 alteration (p < 0.001), indicating rather a driver than age specific effect (SI Table 4 and 5).”

SI Table 4. Evaluation of deconvolution data with tests on individual logratios between driver groups in patients >39 years of age. Adj.p. = adjusted p value, Est = estimate, T reg = T regulator cell

	NF2 vs YAP1	NF2 vs YAP1	SMARCE1 vs YAP1	SMARCE1 vs YAP1	SMARCE1 vs NF2	SMARCE1 vs NF2
	est.	adj.p.	est.	adj.p.	est.	adj.p.
B cell	0.86	0.021	2.12	<0.001	1.26	0.016
Basophil	1.72	0.339	1.74	0.412	0.02	0.972
Blood Cell Progenitor	1.48	<0.001	2.73	<0.001	1.24	0.099
Cancer	1.40	<0.001	1.78	<0.001	0.38	0.380
CD4 T-cell	-0.30	0.976	0.20	0.907	0.49	0.380
CD8 T-cell	0.28	0.976	-0.10	0.907	-0.38	0.434
Endothelium	0.81	0.039	-1.72	0.027	-2.53	0.006
Eosinophil	0.11	0.980	0.74	0.690	0.63	0.255
Fibroblast	-1.28	0.009	-3.02	<0.001	-1.74	0.016
Glia	-0.06	0.976	-0.56	0.412	-0.50	0.434
Microglia	-0.74	0.052	-1.50	0.004	-0.76	0.191
Monocyte	-2.74	0.002	-3.06	<0.001	-0.32	0.668
Natural Killer	-1.71	0.328	-1.32	0.427	0.39	0.477
Neuron	-1.43	0.018	-0.32	0.702	1.12	0.099
Neutrophil	1.57	0.339	1.93	0.294	0.36	0.576
T reg	0.03	0.983	0.36	0.907	0.33	0.667

Statistical metrics (adjusted p-values) should be clearly annotated on Figures 2C and 3B.

<u>Modifications:</u>

- Adjusted p-values were included in Figure 2C and 3B

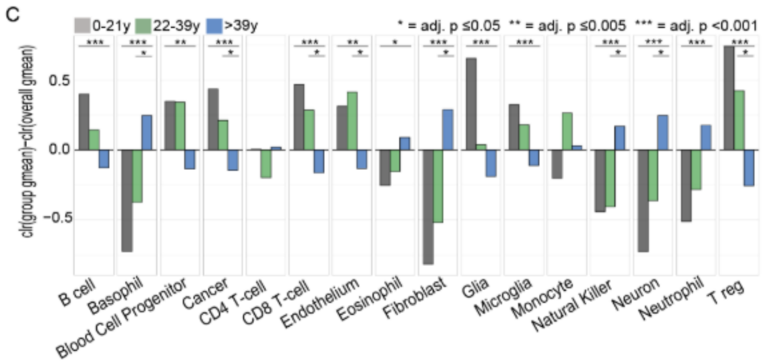

Figure 2. [...] Compositional data analysis plot based on bulk methylation deconvolution data for meningiomas from patients 0-21 years, 22-39 years and adults (>39 years). clr = centered log ratio. P-values were retrieved from SI Table 2. [...]

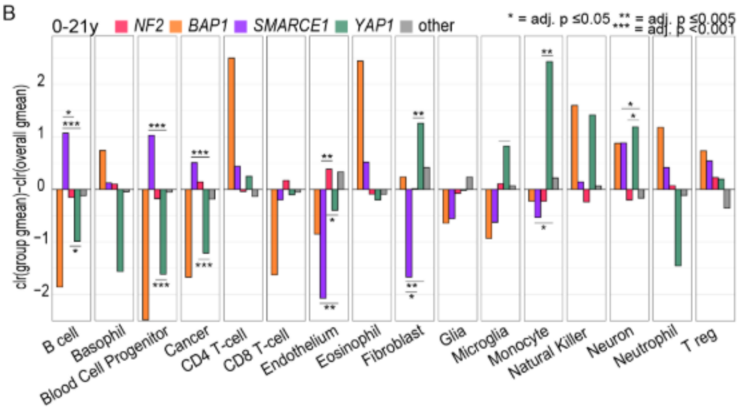

Figure 3. [...] Compositional data analysis plot based on bulk methylation deconvolution data for meningiomas from patients 0-21 years, stratified according to their driver mutation. The "other" subgroup also contains meningiomas with unknown BAP1 status. clr = centered log ratio. P-values retrieved from SI Table 4. [...]

5. Several statements lack statistical support
For example: "Cluster 4 consisted of SMARCE1-altered meningiomas and cluster 3 contained BAP1-altered meningiomas. Cluster 1 and area B were enriched for NF2-mutated meningiomas."
Please provide enrichment statistics (Fisher's exact p-values).
Similarly, several descriptive claims regarding methylation clustering and driver enrichment should include formal statistical support.

Response: We thank the reviewer for this important comment and agree that the original wording regarding cluster composition was potentially misleading and required clarification.
Specifically, *BAP1-* and *SMARCE1*-driven meningiomas form highly distinct methylation classes that are characterized by the presence of the respective alterations and are not observed across other clusters, consistent with previous reports (PMID: 40249111; PMID: 33319313). We have revised the manuscript to more accurately reflect this clear segregation.
In contrast, *NF2*-altered meningiomas do not exhibit such a distinct clustering pattern. As also described above (see also point 3), *NF2*-altered cases are distributed across multiple methylation classes and do not segregate into a single, well-defined cluster, consistent with prior studies. This heterogeneity is observed both in the context of germline and non-germline alterations. We have removed the statement.

Modifications:

- We have revised the results section (subheading: "Driver alterations influence the epigenetic clustering ")
  "Cluster 4 consisted of SMARCE1-altered meningiomas and cluster 3 contained the BAP1-altered meningiomas. Both clusters have already been described and were found to be significantly associated with younger patients[20,31]~~.~~

Many of the statistical tests heavily on univariable analysis. Multivariable models should be used to test for test age, driver, CNV, EOR together?

Response: We analyzed the prognostic value of established/competing risk stratifications/factors in univariable models to compare their impact between age cohorts and identify those being potentially relevant to patients 0-21 years. We added a multivariable Cox model for the newly proposed risk stratification in patients 0-21 years.
In addition, we also added a multivariable analysis for TME to assess the effect of age group and driver mutation/MC on individual TME components within one model. For each individual TME component, a multivariable linear model with the TME component (using PLR1 transformation) as outcome is fitted. I.e., each column in the plot gives estimates from one

multivariable model. Only significant effects are displayed. P-values are adjusted across TME components. Red/blue indicates a relative increase/decrease of a TME component associated with a certain factor. EOR is not included because no effect on TME was seen in the subgroup of samples with EOR information and to avoid reduced sample size in multivariable models due to missing information of EOR. We also included a multivariable model and propensity score weighted analysis to analyse the effect of radiotherapy

<u>Modifications: multivariable risk model:</u>

- We applied the multivariable risk model and updated the results section (subheadline: Integrated, hierarchical risk stratification reveals distinct prognostic subgroups in pediatric and AYA meningiomas”
  “When applying the risk stratification displayed in Figure 5A to the presented cohort of 0-21 years of age patients, only low (n = 48) and intermediate (n = 48) cases can be compared since there are no BAP1 altered cases in this group. In a multivariable Cox PH model including risk stratification, extent of resection, WHO grade and sex, only intermediate risk (HR: 2.5, 95%CI 1.2-5.2, p = 0.02) and extent of resection (Simpson grade 4-5, HR: 2.9, 95%CI 1.3-6.1, p = 0.001) remained significant risk factors (SI Table 8).”
- We updated the methods section (subheadline: statistics):
  “For the newly established risk model (Fig 5A) for patients 0-21 years of age, a multivariable Cox PH model with Firth correction was fitted to address the potential bias due to the low events per variable ratio. With 5 parameters to be estimated and 39 events, the event per variable (EPV) ratio of 7.8 was low but acceptable. Missing value imputation was done using MICE[46], based on 50 imputations runs. Proportional hazard assumptions were verified based on the scaled Schoenfeld residual approach[47]. The c-index was used to assess prognostic discrimination of different risk stratifications. Propensity score weighted Cox regression based on multiply imputed data sets was performed using R packages MatchThem, cobalt and Weightit. The propensity score weights were estimated with a logistic regression model using age, sex, EOR, WHO grade and diagnosis timepoint as predictors.”
- We updated Figure 4 and Figure 5 in concordance with the new risk model

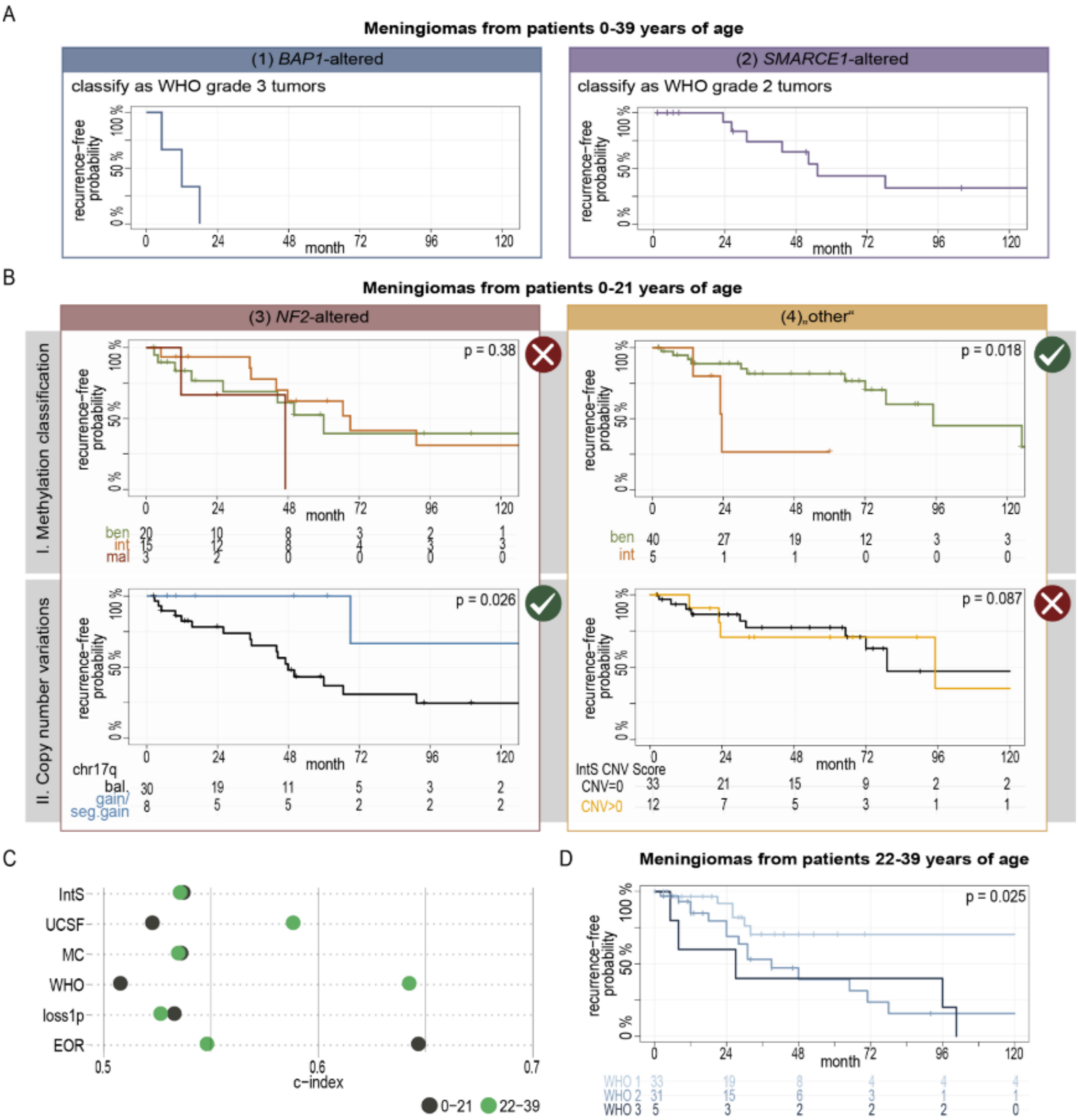

Figure 4. (A) (1) *BAP1*-altered meningiomas show a highly aggressive clinical behavior and should be classified as WHO grade 3 (Sievers et al. (2025)). The Kaplan–Meier analysis included all cases from 0–39 years. (2) *SMARCE1*-altered meningiomas, representing a distinct molecular entity and should be classified as WHO grade 2 (Sievers et al (2021)). The Kaplan–Meier analysis included patients from 0–39 years. (B) Patient group 0-21 years of age: (3) *NF2* altered meningiomas; and (4) tumors not falling into these categories, classified as “other”. The “other subgroup also contains meningiomas with unknown *BAP1* status. The Kaplan–Meier analyses of the progression risk for the *NF2*-altered and “other” subgroups of patients 0-21 years of age, stratified by I. methylation class, ben = benign, int = intermediate, mal = malignant, II. by copy-number alterations: chromosome 17q gain for the *NF2* altered subgroup and the presence of high-risk copy number variations as defined by the Integrated Risk Score for meningiomas for the “other” subgroup. chr = chromosome, bal. = balanced, seg.gain = segmental gain, IntS = Integrated risk score, CNV = copy number variation. The p-values were calculated using the Log-rank test. (C) Comparison of the c-index among the patient groups 0-21-years-old and 22-39-years-old across different stratification systems. IntS = Integrated meningioma risk score by Maas et al. (2021), UCSF = UCSF methylation classes by Choudhury et al. (2022), MC = Heidelberg methylation classes by Sill et al. (2026), WHO = WHO grades, loss1p = loss of chromosome 1p, EOR = Extend of resection based on Simpson Grading. (D) Kaplan-Meier analysis of risk of progression of the subgroup of patients 22–39 years, stratified according to WHO grade. The p-values were calculated using the Log-rank test.

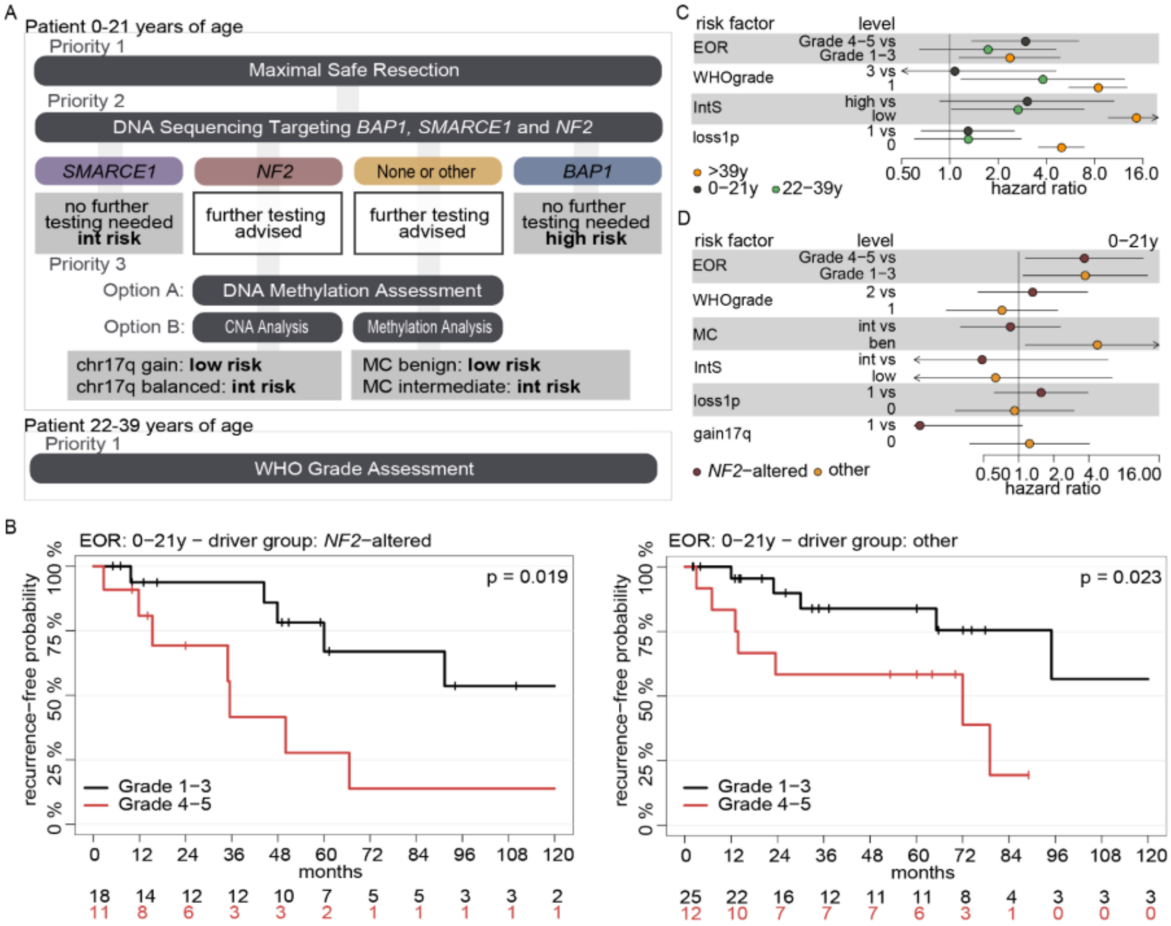

Figure 5. (A) Workflow for efficient meningioma evaluation in patients aged 0–21 years in diagnostic routine. Maximal safe resection remains the highest priority for reducing recurrence risk, and detailed documentation of the extent of resection is essential for accurately assessing and defining the individual risk of recurrence. As a second priority, DNA panel sequencing should be performed to detect alterations in *SMARCE1, BAP1* and *NF2*. Meningiomas harboring *SMARCE1* or *BAP1* alterations do not require additional molecular testing and can be stratified as intermediate-risk and high-risk for recurrence, respectively. For *NF2*-altered meningiomas and meningiomas without alterations in *SMARCE1, BAP1* or *NF2*, further molecular workup can follow two alternative paths: Option A (feasible for both subgroups): Methylation data can be generated using, for example, nanopore sequencing or methylation array profiling, allowing the simultaneous assessment of copy-number variation and methylation class. Option B (subgroup-specific): For *NF2*-altered cases, targeted analysis of chromosome 17q copy-number status (e.g., via FISH) can be performed, while for *NF2*-wild-type / other cases, methylation profiling alone is sufficient to enable subsequent stratification into low- and intermediate-risk groups. CNA: copy number alteration, in = intermediate, MC = methylation class, chr = chromosome. For patients aged 22–39 years, the WHO graduation has the highest priority. (B) Kaplan–Meier analysis for the patient group 0-21 years of age for the driver groups "*NF2*-altered" and "other", stratified for extent of resection Simpson grade 1-3 and 4-5. EOR = extend of resection, y = years, The p-values were calculated using the Log-rank test. (C) Summary of risk factors and their corresponding hazard ratios for the age groups 0-21 years, 22-39 years and >39 years. EOR = extend of resection based on Simpson Grading, IntS = Integrated meningioma risk score by Maas et al. (2021), y = years. (D) Summary of risk factors and their corresponding hazard ratios in *NF2* altered meningiomas and "other" meningioma (0-21 years). The "other subgroup also contains meningiomas with unknown *BAP1* status. EOR = extend of resection based on Simpson Grading, MC = methylation class, ben = benign, int = intermediate, mal = malignant, IntS = Integrated meningioma risk score, y = years.

Modifications: multivariable TME model:

- We revised the results section to include the results from the multivariable TME model: *"Also, when fitting a multivariable linear model for each TME component and including age and MC or driver status as covariates, there was no significant difference in the comparison between the 0-21 and 22-39-years-of-age groups (SI Fig. 3A)."* (subheading: "Patient age influence DNA methylation clustering of meningiomas and cellular composition")
  "This also holds true when fitting a multivariable model (SI Fig. 3E) and when comparing adult cases with either an NF2 or SMARCE1 alteration (p < 0.001), indicating rather a driver

than age specific effect (SI Table 4 and 5).“ (subheading: “Driver alterations shape tumor composition”):

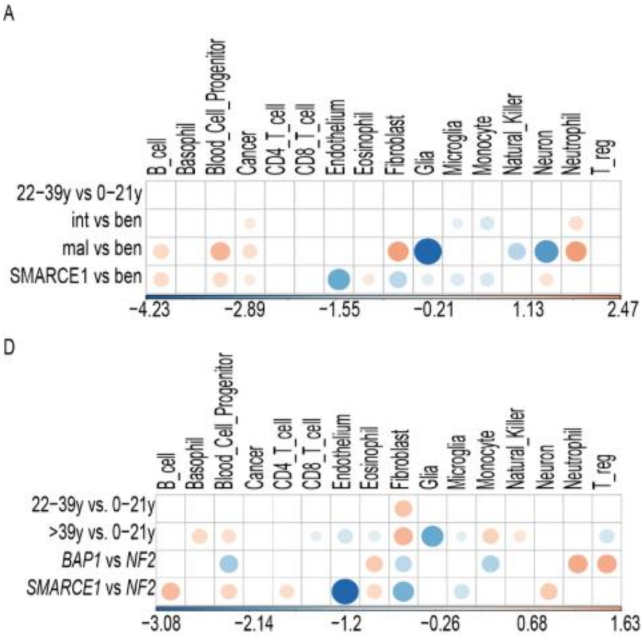

SI Figure 3 (A) multivariable analysis for tumor microenvironment for age cohort and methylation classes, patients 0-39 years: For each individual tumor microenvironment component, a multivariable linear model with the tumor microenvironment component (using PLR1 transformation) as outcome is fitted. I.e., ach column in the plot gives estimates from one multivariable model. Only significant effects are displayed. P-values are adjusted across tumor microenvironment components. Red/blue indicates a relative increase/decrease of a tumor microenvironment component associated with a certain factor. MC = methylation class. [...]
(D) multivariable analysis for tumor microenvironment for age cohort and driver group, all patients excluding driver group other (0-39 years, n = 267): For each individual tumor microenvironment component, a multivariable linear model with the TME component (using PLR1 transformation) as outcome is fitted. I.e., ach column in the plot gives estimates from one multivariable model. Only significant effects are displayed. P-values are adjusted across tumor microenvironment components. Red/blue indicates a relative increase/decrease of a tumor microenvironment component associated with a certain factor. MC = methylation class. [...]

Modifications: models for RT evaluation:

- We revised the results section to include the advanced analysis concerning the impact of radiotherapy (subheading: “Surgical Extent as Primary Prognostic Driver Within an Age-Adapted Stratification Framework”):

  “Besides extent of resection, radiotherapy is an important prognostic factor; however, detailed information on its timing and intent was inconsistently available (Supplementary Table 6). Patients receiving adjuvant radiotherapy more frequently had unfavorable tumor characteristics, including higher WHO grade and Simpson grade, indicating confounding by indication. After adjustment for these factors and diagnosis timepoint, adjuvant radiotherapy remained associated with a non-significant increase in risk in both multivariable and propensity score–weighted analyses (SI Fig. 4A). This likely reflects residual confounding and selection bias rather than a causal effect.”
- We included a figure for the Propensity score analysis in 0-21-year-old patients with missing value imputation and diagnosis time point as confounder in SI Figure 4 A

A

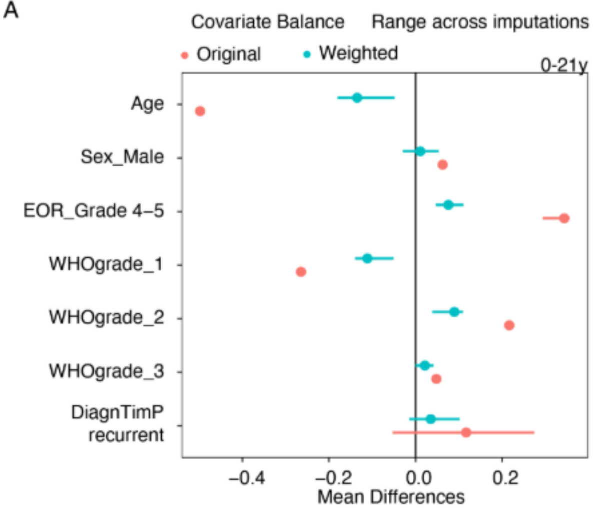

SI Figure 4. (A) 'love' plot showing the covariate balance between patients receiving/not receiving radiotherapy before and after propensity score (PS) weighting across imputed data sets in patients 0-21years. EOR = extend of resection based on Simpson grading. DiagnTimP = Timepoint of Diagnosis. [...]

Minor Comments

1. The first sentence of "Driver alterations influence the epigenetic clustering" section is unclear and could be revised.

Response and Modification: We thank the reviewer for the note and have revised the sentence:

"Known high-risk alterations such as homozygous CDKN2A/B deletions (n = 1, age: 36 years) and TERT promotor mutations (n = 2, age: 33 and 38 years) were absent in meningiomas from 0-21-year-old patients."

2. The term "reference cohort" for adult meningiomas is confusing, especially in methylation analysis. Consider replacing with "adult comparison cohort."

Response and Modification: We thank the reviewer for the comment and have changed it accordingly.

- *"In the adult comparison cohort, consisting of already published data from patients >39 years (n = 747)[23], the median age was 60.8 years (SD: 11.3 years)."* (results section, subheadline: "Age-associated differences in clinical and histopathological features of meningiomas")
- *"As previously described[2,24,25], age-related differences in the female-to-male ratios were observed: the 0-21-year-old group showed a female-to-male ratio of 0.9, which increased to 3.1 in the 22-39-year-old cohort and to 2.1 in the adult comparison cohort (0-21 years vs. 22-39 years p = 0.006; 0-21 years vs. >39 years p <0.001)."* (results section, subheadline: "Age-associated differences in clinical and histopathological features of meningiomas")
- *"While in the adult comparison cohort the loss of chr22q was followed by loss of chr1p, the oncogenetic tree for the 0-21 years of age group showed an additional gain of chr17q at this position (18.4%, >39 years: 9.8%, p <0.001), for the 22-39 years of age group a loss of chr8p or chr8q was indicated (22-39 years: 13.6%, >39 years: 9.8%, p = 0.3; SI Fig. 2A-C)."* (results section, subheadline: "Established adult risk stratification systems lack prognostic value in younger patients")
- *"This contrasts with the adult comparison cohort cases, in which high versus low microglial content shows clear associations with clinical outcomes (Fig. 2E)."* (results section,

subheadline: “Patient age influence DNA methylation clustering of meningiomas and cellular composition”)

3. In Figure 1A, inclusion of mutational status (NF2, BAP1, SMARCE1, YAP1, germline) and CNV status (chr17p, 1p etc.) would strengthen integration of molecular findings.

Response and Modification: We thank the reviewer for the remark and have included the mutation and chromosome status in the figure accordingly.

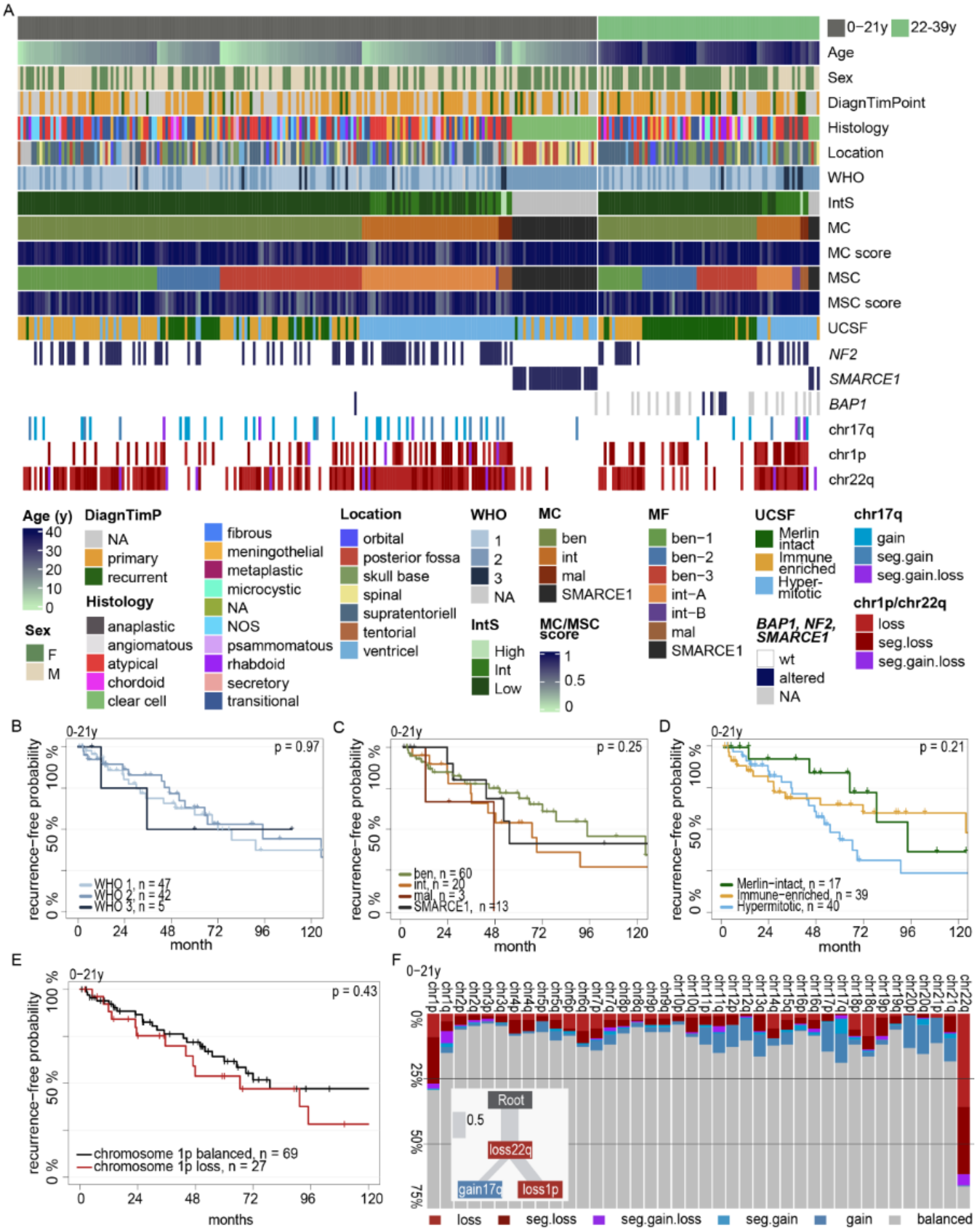

Figure 1. (A) Oncoprint representation of 293 meningioma samples from patients 0-39 years of age. y = year, DiagnTimPoint = Timepoint of Diagnosis, IntS = Integrated Risk Score system proposed by Maas et al. (2021), MC

= methylation class by Sill et al. (2026), MSC = methylation subclass, F = female, M = male, NA = not available, NOS = not otherwise specified, ben = benign, int = intermediate, mal = malignant, UCSF = meningioma classification system proposed by Choudhury et al. (2022), chr = chromosome, seg = segmental, wt = wildtype, seg. = segmental [...]

4. Several figures lack explicit definition of age groups in legends.

Response and Modification: We thank the reviewer for the comment and have added the necessary information to the captions and figures accordingly.

Point-by-point response to the reviewers' comments – 2:

Manuscript: "Distinct molecular subgroups in pediatric and young-onset meningiomas require age-adapted risk stratification"

Reviewer #1

Thank you for the careful revision and detailed rebuttal. The manuscript is substantially improved and now clinically much more convincing. In particular, you clearly define EOR using surgeon-reported Simpson grading (I–III vs IV–V) , strengthen the survival analysis with an adjusted Cox model (including Firth correction, imputation and PH checks) , and appropriately present EOR as the dominant prognostic determinant in patients ≤21 years . The radiotherapy section is also handled cautiously with transparent acknowledgement of confounding by indication .
I am happy to recommend acceptance, with only minor editorial polishing to ensure the abstract/discussion consistently frame molecular stratification as risk refinement in the context of strong clinical determinants (especially EOR).

Response: We sincerely thank the reviewer for their positive assessment of our revised manuscript and for acknowledging the improvements made during the revision. We appreciate the constructive feedback and have further polished the abstract and discussion to consistently frame molecular stratification as risk refinement in the context of strong clinical determinants, particularly extent of resection.

Addition / Modifications:

- we have modified the abstract to more consistently frame molecular stratification as risk refinement in the context of strong clinical determinants: *"In patients ≤21 years, extent of resection is the dominant predictor of outcome, while molecular features further refine risk assessment. These findings define pediatric and young adult meningiomas as a distinct molecular entity and support age-adapted risk refinement that integrates molecular features with strong clinical determinants."*
- we have revised the discussion to clarify that molecular stratification should be understood as risk refinement within the context of strong clinical determinants: *"While extent of resection remains the principal determinant of outcome in tumors arising in younger patients, our findings suggest that molecular stratification provides additional biological context and may refine risk assessment when integrated with established clinical determinants."*

Reviewer #2

The authors have provided a detailed and satisfactory point-by-point response to the reviewers' concerns. Most of the major issues raised during the previous round of review have been appropriately addressed, including clarification of clinical variables, refinement of the analytical framework, and suitable tempering of the conclusions.
In my opinion, the revised manuscript is substantially improved and now meets the standards required for publication. I therefore recommend acceptance of the manuscript.

Response: We sincerely thank the reviewer for their positive assessment and constructive feedback. We are pleased that the revisions and point-by-point responses have adequately addressed the concerns raised during the previous round of review.

Reviewer #3

I commend the authors for their thorough and detailed responses. They have fully addressed my concerns. I only have a few minor suggestions/questions:
1. "homozygote deletions" in line 292 should be updated to "homozygous deletions"

Response / Modifications: We thank the reviewer for this careful correction. We have corrected the terminology by replacing “homozygote deletions” with “homozygous deletions.” (chapter: “Driver alterations influence the epigenetic clustering”)

2. Progesterone receptor (PR) staining H-score is initially mentioned to be significantly different between female and male cases in 0-21 age group. Later, in “Driver alterations shape tumor composition” section, authors indicate that NF2 vs SMARCE1 group also showed a difference in PR staining. It is not clear whether this analysis was performed by correcting for gender.

Response: We thank the reviewer for pointing out this important potential confounder. We color-coded the individual data points in the figure according to sex. In addition, we have added a statement to the manuscript clarifying that, due to the limited number of cases, a separate sex-stratified analysis or formal correction for sex could not be performed.

Addition / Modifications: We included a sentence in the chapter “Driver alterations shape tumor composition”, to address this limitation: *”However, due to the limited number of cases, separate sex-stratified statistical analyses could not be performed.”*
